# TUBB4A interacts with MYH9 to protect the nucleus during cell migration and promotes prostate cancer via GSK3β/β-catenin signalling

**DOI:** 10.1038/s41467-022-30409-1

**Published:** 2022-05-19

**Authors:** Song Gao, Shuaibin Wang, Zhiying Zhao, Chao Zhang, Zhicao Liu, Ping Ye, Zhifang Xu, Baozhu Yi, Kai Jiao, Gurudatta A. Naik, Shi Wei, Soroush Rais-Bahrami, Sejong Bae, Wei-Hsiung Yang, Guru Sonpavde, Runhua Liu, Lizhong Wang

**Affiliations:** 1 Department of Genetics, University of Alabama at Birmingham, Birmingham, AL USA; 2 Department of O’Neal Comprehensive Cancer Center, University of Alabama at Birmingham, Birmingham, AL USA; 3 Department of Pathology, University of Alabama at Birmingham, Birmingham, AL USA; 4 Department of Urology, University of Alabama at Birmingham, Birmingham, AL USA; 5 Department of Radiology, University of Alabama at Birmingham, Birmingham, AL USA; 6 Department of Medicine, University of Alabama at Birmingham, Birmingham, AL USA; 7 Department of Biomedical Sciences, Mercer University School of Medicine, Savannah, GA USA; 8 Dana Farber Cancer Institute, Boston, MA USA

**Keywords:** Prostate cancer, Prostate, Metastasis

## Abstract

Human tubulin beta class IVa (TUBB4A) is a member of the β-tubulin family. In most normal tissues, expression of TUBB4A is little to none, but it is highly expressed in human prostate cancer. Here we show that high expression levels of TUBB4A are associated with aggressive prostate cancers and poor patient survival, especially for African-American men. Additionally, in prostate cancer cells, *TUBB4A* knockout (KO) reduces cell growth and migration but induces DNA damage through increased γH2AX and 53BP1. Furthermore, during constricted cell migration, TUBB4A interacts with MYH9 to protect the nucleus, but either *TUBB4A* KO or *MYH9* knockdown leads to severe DNA damage and reduces the NF-κB signaling response. Also, *TUBB4A* KO retards tumor growth and metastasis. Functional analysis reveals that TUBB4A/GSK3β binds to the N-terminal of MYH9, and that *TUBB4A* KO reduces MYH9-mediated GSK3β ubiquitination and degradation, leading to decreased activation of β-catenin signaling and its relevant epithelial-mesenchymal transition. Likewise, prostate-specific deletion of *Tubb4a* reduces spontaneous tumor growth and metastasis via inhibition of NF-κB, cyclin D1, and c-MYC signaling activation. Our results suggest an oncogenic role of TUBB4A and provide a potentially actionable therapeutic target for prostate cancers with TUBB4A overexpression.

## Abstract (web summary)

The β-tubulin family protein TUBB4A is highly expressed in cancer but it’s molecular role is unclear. Here, the authors show that TUBB4A is required to protect the nucleus from genomic instability during migration and that it’s over expression promotes cancer progression.

## Introduction

There are more than 3.6 million men diagnosed with prostate cancer in the United States, and an estimated ~248,530 new cases will be diagnosed in 2021^1^. Prostate cancer accounts for nearly 26% of all cancer cases and is the second leading cause of death in American men^1^. While prostate-specific antigen screening offers a benefit of reduced prostate cancer mortality, there is, since 2010, an increase in advanced-stage diagnoses across age and race^1^. Most prostate cancers are discovered in the local or regional stages with ~100% 5-year relative survival rate, but the 5-year relative survival for metastatic disease declines to 30%^2^. African American (AA) men have the highest rate of prostate cancer among any ethnic groups^3,4^. They also have a more than 2-fold higher rate of death related in part to more advanced stages^3,4^. However, except for metastasis, causes of the higher mortality rate among AA patients with prostate cancer are largely unknown.

Cell migration is involved in the metastasis of malignant tumors. During migration, tumor cells must pass through a complex and dense tissue three-dimensional (3D) tumor microenvironment (TME). To invade dense tissues, tumor cells reconstruct the TME by protease degradation of the extracellular matrix (ECM). Otherwise, without degrading the ECM, tumor cells squeeze through tissue gaps smaller than their diameter by self-contraction^5^. Likewise, cytoskeleton proteins, such as microtubules, participate in the process of restricted cell migration and play a key role in nuclear protection during cell migration. Microtubules, formed by α- and β-tubulin heterodimers, are involved in numerous cell processes, mainly related to cell division, flagella and cilia movement, and intracellular transport. The kinetics of microtubules changes cancer cell division, chromosomal instability, and aneuploidy^6^. A subtype of β-tubulin participates in molecular movement, leading to stable kinetics of microtubules in cancer cells^7^. The stability of microtubules is related to regulation of cell migration and the epithelial-mesenchymal transition (EMT). Increased levels of α-tubulin acetylation stabilize the cytoskeleton^8^. These reports suggest that, in tumor cells, the subtype of tubulin may be involved in the function of microtubule-based cell movement and the cytoskeleton.

In humans, microtubules are composed of 8 α-tubulin isotypes and 7 β-tubulin isotypes (β1, β2, β3, β4a, β4b, β5, and β6)^9^. The members of the β-tubulin family share a high degree of structural homology and are distinguished from one another by highly divergent sequences at the C-terminal tail^10,11^. Human *tubulin beta class IVa* (*TUBB4A*), a member of the β-tubulin family, encodes the TUBB4A protein. In most normal tissues, expression of TUBB4A protein is little to none, but it is predominantly expressed in the brain, with moderate amounts in testes and adrenal glands. However, *TUBB4A* is highly expressed in human cancer cell lines, including those of the prostate (e.g., PC3), breast (e.g., AN3-CA), and lung (e.g., A549) (Human Protein Atlas). As determined by analysis of The Cancer Genome Atlas (TCGA) dataset, expression of *TUBB4A* is abnormally elevated in prostate, lung, kidney, and uterine endometrial cancers (Fig. S1). *TUBB4A* mutations often occur in neurological diseases, such as myelinopenia, basal ganglia and cerebellar atrophy-related diseases, hereditary spastic paraplegia, and leukodystrophy, resulting in a reduction of growth of neuronal processes^12^. Recent proteome and functional analyses reveal that, in human cancers, including prostate cancer, TUBB4A is involved with cell structural and tubulin-binding processes and resistance to chemo- and radio-therapy^13–16^. Indeed, elevated expression of TUBB4A is associated with a poor response to paclitaxel for patients with ovarian cancer^17^, suggesting a role of TUBB4A in resistance to chemotherapy. These data suggest a role for TUBB4A in chemotherapy resistance of malignant tumors. However, the role of TUBB4A in regulating cell migration as well as tumor metastasis remains elusive. In this work, we address the role of TUBB4A in growth and metastasis of prostate cancer in vitro and in vivo, analyze the TUBB4A-interacting proteins and signaling pathways, and explore the mechanisms underlying TUBB4A-mediated tumor growth and metastasis and disparities. Our results suggest an oncogenic role of TUBB4A and provide a potentially actionable therapeutic target for prostate cancers with TUBB4A overexpression.

## Results

### Characterization of TUBB4A expression profiling in human primary prostate cancer

**Fig. 1:**
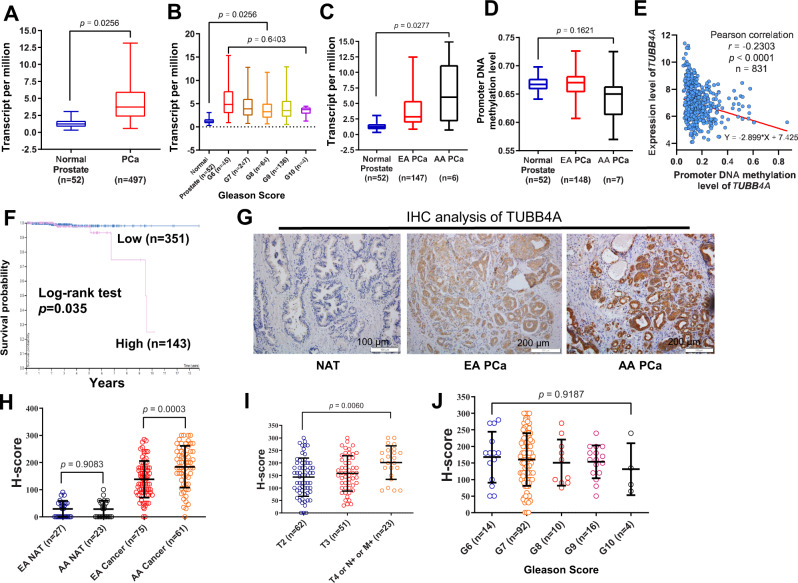
TUBB4A expression in normal prostate and prostate cancer tissues. Differential expression of *TUBB4A* **A** between normal prostate and prostate cancer tissues, **B** between tumor tissues with different Gleason scores, and **C** between AA and EA tumor tissues from TCGA dataset. Numbers in parentheses indicate the sample size. **D** DNA methylation of *TUBB4A* promoter (TCGA dataset). **E** Correlation of mRNA expression levels with DNA methylation levels in the CpG island of *TUBB4A* in prostate cancer samples (TCGA dataset). **F** Kaplan–Meier survival curves for *TUBB4A* expression with low and high levels in TCGA prostate cancer patients. **G** Representative IHC staining in normal prostate and prostate cancer tissues with anti-human TUBB4A mAb (Abcam, ab11315) from UAB prostate cancer specimens. Scale bars, 100 μm or 200 μm. This experiment was repeated two times. **H**–**J** Quantitative H-scores for prostate cancer samples from European and African American ancestry and tumor stages and Gleason scores. Data are presented as the medians and interquartile ranges as determined with a Kruskal–Wallis (**B**–**D**) or Mann–Whitney *U* (**A**–**D**) test. Data are presented as the means and standard deviation (SD) as determined with an ANOVA followed by Tukey’s post hoc *t* test (**H**–**J**). Source data are provided as a Source data file. AA: African-American; EA: European-American; NAT: Normal prostate tissue adjacent to the tumor.

To evaluate the clinical relevance of TUBB4A in human prostate cancer, we conducted a bioinformatics analysis of public datasets from The Cancer Genome Atlas (TCGA). For prostate adenocarcinoma, expression of *TUBB4A* was higher in tumor samples compared with that in normal prostate samples (Fig. 1A) but did not change with Gleason score (Fig. 1B). Of note, higher expression of *TUBB4A* was evident in AA samples relative to European-American (EA) samples (Fig. 1C). Furthermore, using the UCSC Genome Browser, we identified a prominent 5’CpG island (321-bp) of the *TUBB4A* promoter (Fig. S1B). Our further analysis identified higher expression levels of *TUBB4A* mRNA but lower levels of DNA methylation in this CpG island in AA samples compared to EA samples or normal prostate samples (Fig. 1C, D). Of note, average methylation levels in the CpG island negatively correlated with the expression of *TUBB4A* in tumor samples (*r* = −0.29, *p* < 0.001) (Fig. 1E). Survival analysis indicated that patients with high expression of *TUBB4A* had a shorter life than those with low expression of *TUBB4A* (Fig. 1F).

Immunohistochemical (IHC) analysis in The Human Protein Atlas showed that, in most prostate cancer samples (11/12 cases), TUBB4A has strong staining within tumor areas, but low or no staining in normal prostate samples (Fig. S1C). Furthermore, we evaluated, by IHC, the protein expression of TUBB4A in 136 primary prostate adenocarcinoma samples, along with 50 samples of tumor-adjacent normal prostate. As shown in Fig. 1G–J and Table S1, H-Score quantitative analysis showed that TUBB4A was not or minimally expressed in normal prostate tissues adjacent to the tumor (NAT) but highly expressed in tumor samples. Of note, there was higher expression of TUBB4A in AA tumor samples than in EA tumor samples (Fig. 1H). Also, higher expression of TUBB4A was evident in samples of aggressive prostate adenocarcinomas than in localized samples (Fig. 1I), but expression was not significantly associated with Gleason score (Fig. 1J).

In addition, we performed a bioinformatics analysis assessing genetic alterations of *TUBB4A* in primary prostate cancer samples using various datasets from 6 studies, including TCGA dataset. There were genetic alterations of *TUBB4A*, including amplification, deletion, and mutation, in 1.1% of all cases (*n* = 6819). Of note, the *TUBB4A* gene was amplified in neuroendocrine prostate cancers, reaching 12–15% cases and in castration-resistant prostate cancers, reaching 8.57% (Fig. S1D). For localized prostate cancer, only 0.21–3.08% showed amplification. In all samples, mutations and deletions of *TUBB4A* were rare (Fig. S1D).

### *TUBB4A* knockout reduces cell growth and migration in human prostate cancer cells

**Fig. 2:**
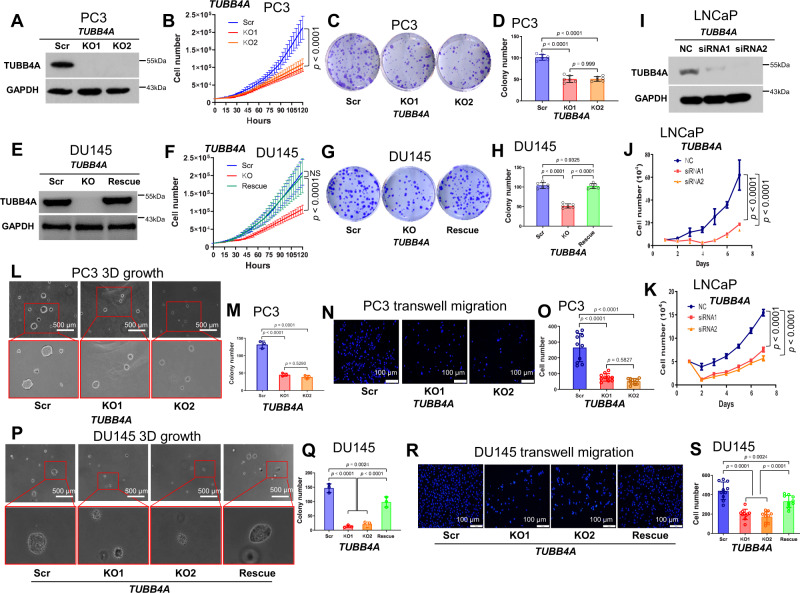
*TUBB4A* KO reduces proliferation and migration of prostate cancer cells. **A**–**D** Confirmation of *TUBB4A* KO, cell growth, clone formation, and statistical analysis of PC3 cells. Cell growth was measured by live cell kinetic imaging with walk-away automation using a Lionheart™ FX cell imager. A colony was considered to be 50 cells or more as determined microscopically. **E** confirmation of *TUBB4A* KO and rescue of DU145 cells. **F**–**H** Cell growth, clone formation, and statistical analysis of DU145 cells. **I**, **J** Confirmation of *TUBB4A* knockdown (KD) and cell growth of LNCaP cells. **K** Cell growth assay of LNCaP cells transfected with *TUBB4A* siRNAs in androgen-depleted culture medium. **L**, **M** 3D soft-agar colony formation and statistical analysis of PC3 cells. A 3D colony was counted based on the capacity of single cells to grow to colonies consisting of at least 50 cells. Scale bar, 500 μm. **N**, **O** Transwell assay and statistical analysis of PC3 cells. Scale bar, 100 μm. **P**, **Q** 3D soft-agar colony formation and statistical analysis of DU145 cells. Scale bar, 500 μm. **R**, **S** Transwell assay and statistical analysis of DU145 cells. Scale bar, 100 μm. Data are replicated 3 (**J**, **K**, **M**, **Q**), 4 (**B**, **F**), 6 (**D**, **H**), and 10 (**O**, **S**) times. Data are presented as the means and SD with a repeated measures ANOVA (**B**, **F**, **J**, **K**) or an ANOVA followed by Tukey’s post hoc *t* test (**D**, **H**, **M**, **O**, **Q**, **S**). Source data are provided as a Source data file. Scr, scramble; KO, knockout; NC, negative control siRNA; siRNA, small interfering RNA; NS, no significant difference.

We first addressed the expression levels of TUBB4A in human prostate cancer cells, including two androgen-independent cell lines, DU145 and PC3, and one androgen-dependent cell line, LNCaP. Immunoblotting analysis showed high expression of TUBB4A in three cell lines (Fig. S2A). Next, we used CRISPR/Cas9 genome editing to knockout (KO) *TUBB4A* in DU145 and PC3 cell lines. Two single guide RNAs (sgRNAs) were designed at the second exon of *TUBB4A* (Fig. S2B), and KO cells were confirmed by Sanger DNA sequencing (Figs. S2C and S2D). For PC3 KO cells, one base (A, adenine) was inserted into each of the two alleles of *TUBB4A*. For DU145 KO cells, both alleles of *TUBB4A* were deleted by 53 nucleotides. The loss of TUBB4A protein was validated in the KO cells by Western blots (Figs. 2A and S2E). Finally, two sgRNAs were accessed using the off-target searching tool (Cas-OFFinder, Daejeon, South Korea, http://www.rgenome.net/cas-offinder)^18^. Although potential off-target regions were identified, no off-target changes were found in these regions using PCR analysis of the KO cells (Figs. S2F, S2G).

Since microtubules are involved in regulation of cell growth and movement^19^, we tested the effect of TUBB4A on these properties. For PC3 cells, decreased cell growth was evident in two *TUBB4A* KO cells compared to scrambled cells using cell growth assays (Fig. 2B) and colony formation assays (Fig. 2C, D). This observation was validated with a 3D cell culture viability assay (Fig. 2L, M). Furthermore, cell migration was reduced in two *TUBB4A* KO PC3 cells compared to scrambled cells as determined with Transwell migration assays (Fig. 2N, O). In particular, movement and migration were slower for *TUBB4A* KO PC3 and DU145 cells than for scrambled cells, as observed with automated live-cell 2D random migration assays (Figs. S3A and S3B and Supplementary Videos 1–4). Likewise, there were similar results for DU145 cells using a scratch migration assay measured by live-cell kinetic imaging with walk-away automation (Figs. S3C and S3D, and supplementary Videos 5–7). Next, we rescued the expression of TUBB4A in KO DU145 and PC3 cells (Figs. 2E and S4A). For the rescued cells, growth and migration were increased by the ectopic expression of TUBB4A (Figs. 2F–H, P–S and S4B–H). In addition, there were similar results for LNCaP cells after *TUBB4A* knockdown (KD) by small interfering RNAs (siRNAs) (Fig. 2I, J). However, TUBB4A-mediated cell growth of LNCaP cells was not blocked by androgen depletion (Figs. 2K and S4I), suggesting that this growth is androgen-independent.

### *TUBB4A* knockout changes cytoskeleton and increases DNA damage during migration of prostate cancer cells

**Fig. 3:**
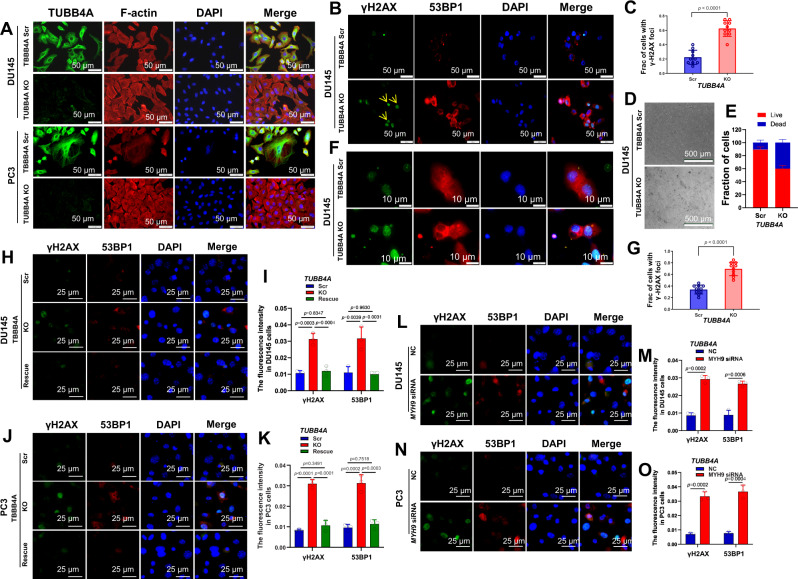
*TUBB4A* KO and MYH9 KD increase DNA damage and cell death during migration of prostate cancer cells. **A** Immunofluorescence (IF) staining of TUBB4A and F-actin in DU145 and PC3 cells with or without *TUBB4A*. Scale bar, 50 μm. **B**, **C** IF staining of γH2AX and 53BP1 in DU145 cells with or without *TUBB4A* during migration in 3D extracellular matrix. Yellow arrows indicate cells with DNA damage. Scale bar, 50 μm. **D**, **E** Death rate of DU145 cells with or without *TUBB4A* after migration in 3D collagen gels. Scale bar, 500 μm. **F**, **G** IF staining of γH2AX and 53BP1 in DU145 cells with or without *TUBB4A* after migration in 3D collagen gels. Scale bar, 10 μm. **H**–**K** IF staining of γH2AX and 53BP1 in scrambled, *TUBB4A* KO, and rescued DU145 and PC3 cells during migration in a 3D extracellular matrix. Scale bar, 25 μm. **L**–**O** IF staining of γH2AX and 53BP1 in scramble control and *MYH9* KD DU145 and PC3 cells during migration in a 3D extracellular matrix. Scale bar, 25 μm. Data are replicated 3 (**I**, **K**, **M**, **O**) and 10 (**C**, **G**) times. Data are presented as the means and SD with a two-tailed *t* test (**C**, **G**, **M**, **O**) or an ANOVA followed by Tukey’s post hoc *t* test (**I**–**K**). Source data are provided as a Source data file. Scr, scramble; KO, knockout; NC, negative control siRNA; siRNA, small interfering RNA; KD, knockdown; NS, no significant difference.

Microfilaments (actin filaments) and microtubules (α- and β-tubulin) combine to form the cytoskeletons of cells^20^. To determine the effect of TUBB4A on the actin cytoskeleton, we monitored, for DU145 and PC3 cells during migration, actin polymerization through F-actin staining upon *TUBB4A* KO. Immunofluorescence (IF) analysis showed a difference in the F-actin distribution between scrambled and *TUBB4A* KO cells (Fig. 3A). For scrambled and *TUBB4A* KO DU145 and PC3 cells, we quantified the cell areas and intensities and distributions of F-actin. As shown in Figs. S5A–J, the cell areas and F-actin intensities were elevated in the KO cells compared to scrambled cells. Of note, the intensity of F-actin over the nucleus was higher in the KO cells compared to scrambled cells. To test whether TUBB4A has a protective effect on the nucleus, we allowed scrambled and *TUBB4A* KO DU145 cells to migrate through an 8-μm 3D ECM Transwell membrane. The migrated cells were evaluated by IF staining for DNA damage proteins, γH2AX and 53BP1. DNA damage was more extensive in *TUBB4A* KO cells than in scrambled cells (Fig. 3B, C, see yellow arrows), suggesting a protective effect of TUBB4A on the nucleus during migration. Since actin stress fiber organization promotes cell stiffening and proliferation of pre-invasive cancer cells^21^, the TUBB4A defects likely resulted in an increased accumulation of F-actin around the nucleus to protect it. This may be feedback from the cells to protect the nucleus by adding F-actin as a replacement for the lack of TUBB4A during cell migration.

To confirm this observation, we used a 3D collagen ECM assay to measure cell survival during migration. After cells penetrated the collagen for 20 h, the percentage of cell death was higher for *TUBB4A* KO cells than for scrambled cells (Fig. 3D, E). Likewise, during 3D collagen migration, increased DNA damage was evident in *TUBB4A* KO cells but not scrambled cells (Fig. 3F, G). Likewise, with the scrambled and *TUBB4A* KO DU145 and PC3 cells, we performed rescue experiments for DNA damage. Quantitative analysis showed that depletion of TUBB4A increased DNA damage during constricted migration of DU145 and PC3 cells (Fig. 3H–K). However, in *TUBB4A* KO cells, exogenous expression of TUBB4A decreased DNA damage (Fig. 3H–K). To test the effect of TUBB4A on α-tubulin, we monitored the cell morphology through α-tubulin staining upon *TUBB4A* KO during cell migration. However, no obvious changes were determined in expression levels and distribution of α-tubulin between KO and scrambled cells (Fig. S6A). In addition, lamin proteins contribute to protection of nuclei^22^ and are involved in nuclear stability, chromatin structure, and DNA damage^23^. Likewise, expression levels and distribution of lamin A/C were not changed during cell migration (Fig. S6B). Thus, the effect of TUBB4A on the cytoskeleton and nuclei may be independent of α-tubulin and lamin.

### TUBB4A interacts with MYH9 to protect the nucleus during migration of prostate cancer cells

**Fig. 4:**
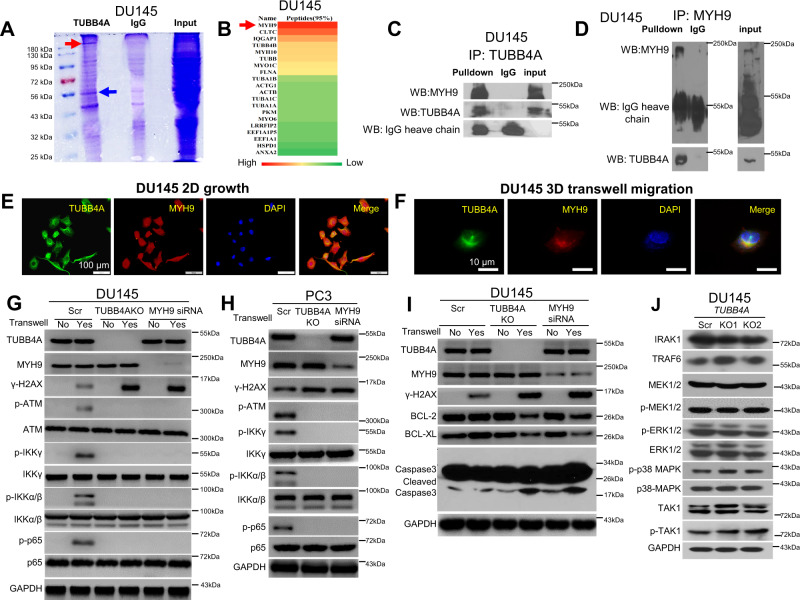
TUBB4A interacts with MYH9 and their relevant DNA damage response and signaling pathways in prostate cancer cells. **A** Bands on an SDS-PAGE gel after TUBB4A protein pull-down in DU145 cells. Red and blue arrows indicate the bands presumed to be MYH9 and TUBB4A, respectively, in the pulldown. **B** TUBB4A-interacting proteins as determined by mass spectrometry analysis. Red arrow indicates MYH9 and its potential site on SDS-PAGE gel. **C**, **D** Co-immunoprecipitation (IP) assay of TUBB4A and MYH9. The input was diluted as 1/10. **E** Co-localization of TUBB4A and MYH9 by IF staining. Scale bar, 100 μm. **F** Co-localization of TUBB4A and MYH9 at the forefront of the nucleus during migration in 3D collagen gels determined by IF staining. Scale bar, 10 μm. **G**, **H** Western blotting showing changes in proteins of DNA damage response and proteins of the NF-κB p65 and IKK complex in DU145 and PC3 cells with or without *TUBB4A* and *MYH9*. **I** Western blotting showing changes in proteins of DNA damage response and proteins of the intrinsic apoptosis pathway in DU145 cells with or without *TUBB4A* and *MYH9*. **J** Western blotting showing changes in proteins of NF-kB-relevant signaling pathways in DU145 cells with or without *TUBB4A* and *MYH9*. Source data are provided as a Source data file. Scr, scramble; KO, knockout; NC, negative control siRNA; siRNA, small interfering RNA; NS, no significant difference.

As shown in Fig. 4A, B and Table S2, mass spectrometry analysis of TUBB4A protein complexes identified TUBB4A-related proteins in DU145 cells. MYH9 (myosin heavy chain 9) had the most binding peptide segments with TUBB4A. MYH9 function is manifested in conventional actin-related processes^24^, requiring translocation of the actin cytoskeleton^25^. Next, we assessed the interaction between TUBB4A and MYH9 by immunoprecipitation (Fig. 4C, D). The co-localization of TUBB4A and MYH9 was confirmed by IF staining (Fig. 4E). In 3D collagen migration, TUBB4A and MYH9 were co-localized around the nucleus and accumulated at the forefront of the nucleus on cytosol (Fig. 4F). TUBB4A, a member of the microtubule family, may participate in a structural framework to protect the nucleus in cell movement. Our data support the interaction of TUBB4A and MYH9 and their mediated protection of the nucleus during cell migration. In addition, we knocked down *MYH9* by siRNA in DU145 and PC3 cells and assessed the *MYH9* KD-induced DNA damage during constricted cell migration. As shown in Fig. 3L–O, *MYH9* KD-induced DNA damage was present during constricted cell migration. These data indicate that, during constricted cell migration, TUBB4A/MYH9 and their interaction protects cells against DNA damage. Cell migration may be a source of DNA damage, and *TUBB4A* KO or *MYH9* KD makes it more extensive.

### *TUBB4A* knockout leads to increased DNA damage but decreased NF-κB signaling during migration of prostate cancer cells

To determine the TUBB4A-related gene network and potential signaling pathways, we performed integrated bioinformatics analysis using PAGER2^26^. As shown in Fig. S7A, there were 22 genes as shared interactors between the two seed genes, *MYH9* and *TUBB4A*. Further, we identified the top 10 significantly enriched NCI-pathways ordered by *p*-values; the most significantly enriched was the canonical NF-κB signaling pathway (false discovery rate (FDR) *p*-value = 1.01e−28, Fig. S7B). In the top canonical NF-κB signaling pathway, 23 genes and 113 interactions regulated the gene network (Fig. S7C); these may contribute to TUBB4A/MYH9-mediated DNA damage.

Next, with DU145 and PC3 cells, we performed analyses to elucidate the mechanistic link involving TUBB4A/MYH9 with NF-kB signaling and DNA damage. As shown in Fig. 4G, H, for *TUBB4A* or *MYH9* scramble control cells, Transwell cell migration resulted in DNA damage (γH2AX) in migrated cells. However, migration caused more severe DNA damage (γH2AX) in *TUBB4A* KO or *MYH9* KD cells than in scrambled cells. Furthermore, in migrated scramble control cells, DNA damage led to an NF-kB signaling response and activation, including increased phosphorylation of ATM, the IKK complex (IKKα/β/γ), and p65 (Fig. 4G, H). However, in migrated *TUBB4A* KO or *MYH9* KD cells, there was no NF-kB signaling response and activation, suggesting that *TUBB4A* KO or *MYH9* KD-mediated DNA damage disrupts the ATM/IKK/p65 signaling response and activation. In addition, in *TUBB4A* KO or *MYH9* KD cells compared with scramble control cells after migration, there was increased DNA damage and cleaved caspase-3 but decreased anti-apoptotic proteins, including BCL2 and BCL-XL (Fig. 4I). With DU145 cells, we assessed the effect of *TUBB4A* KO on NF-kB up-stream proteins and other NF-kB-relevant pathways, including the mitogen-activated protein kinases/extracellular signal-regulated kinase (MAPK/ERK) and mitogen-activated protein kinase kinase 7 (MAP3K7) signaling pathways. However, the expressions of IRAK1, TRAF6, and total and phosphorylated MEK, ERK1/2, p38MAPK, and MAP3K7 were not changed after *TUBB4A* KO (Fig. 4J). These data suggest that TUBB4A/MYH9 are unlikely to regulate the NF-kB pathway directly. Regulation is most likely to be a subsequent response, and, during cell migration, *TUBB4A* KO or *MYH9* KD may disrupt DNA damage-induced NF-kB signaling response and activation. Thus, constricted cell migration-mediated DNA damage may be a link involving TUBB4A/MYH9 with NF-kB signaling (Fig. S7E).

### TUBB4A facilitates MYH9-mediated GSK3β/β-catenin signaling activation in prostate cancer cells

There is direct binding of MYH9 to GSK3β, and silencing of *MYH9* blocks GSK3β ubiquitination and degradation and β-catenin activation, leading to EMT signals^27–30^. Our bioinformatics analysis from TCGA dataset showed, for prostate cancer tissues, a positive correlation of mRNA expression of *MYH9* with mRNA expression of *GSK3β*, *β-catenin*, *cyclin D1*, *c-MYC*, *vimentin*, and *N-cadherin* (Figs. S8A–F). To determine the role of endogenous MYH9 in growth of prostate cancer cells, we performed proliferation assays for DU145, PC3, and LNCaP scrambled and *MYH9* KD cells. As shown in Figs. S8G–L, cell growth was slower for *MYH9* KD cells compared to scramble control cells, suggesting an oncogenic role of MYH9 in prostate cancer cells.

**Fig. 5:**
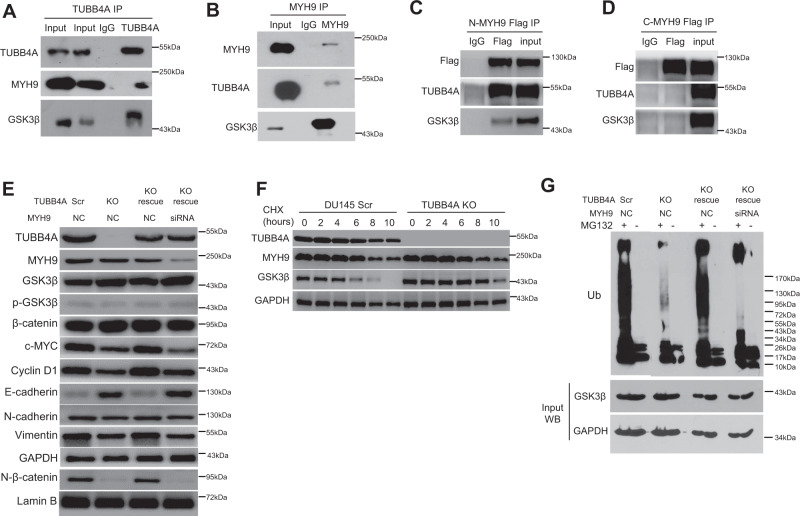
TUBB4A/MYH9-regulated GSK3β/β-catenin signaling pathway in prostate cancer cells. **A**, **B** Co-IP assay of TUBB4A and MYH9 in DU145 cells. The input was diluted at 1/10. **C**, **D** Co-IP assay of TUBB4A and N-terminal (N-MYH9) or C-terminal (C-MYH9) of MYH9-Flag in DU145 cells. **E** Western blotting for changes in proteins of the GSK3β/β-catenin signaling pathway and EMT markers in DU145 cells with or without *TUBB4A* and *MYH9*. **F** Western blotting showing the effect of *TUBB4A* KO on MYH9 and GSK3β stability in DU145 cells incubated with cycloheximide (CHX) at the indicated time points. **G** Ubiquitination of GSK3β in DU145 cells with or without *TUBB4A* and *MYH9*. Cells were either untreated or treated with proteasome inhibitor MG132 (10 μM) for 12 h. Equal aliquots of the cellular lysates were immunoprecipitated with anti-GSK3β antibodies. Source data are provided as a Source data file. Scr, scramble; KO, knockout; NC, negative control siRNA; siRNA, small interfering RNA.

To elucidate the molecular mechanism of TUBB4A/MYH9-mediated tumor progression, we assessed, with DU145 cells, the role of TUBB4A/MYH9 on the GSK3β/β-catenin signaling pathway by immunoblotting analysis. As shown in Fig. 5A–D, TUBB4A/GSK3β bound to the N-terminal of MYH9. In DU145 cells, *TUBB4A* KO or *MYH9* KD increased the expression of GSK3β but decreased the expression of nuclear β-catenin and its downstream targets, cyclin D1 and c-MYC (Fig. 5E), suggesting TUBB4A/MYH9-mediated GSK3β/β-catenin signaling in human cancer cells (Fig. S7E). *TUBB4A* KO reduced GSK3β ubiquitination and degradation (Fig. 5F, G). In addition, we assessed the effect of *TUBB4A* KO on the EMT in DU145 cells. After *TUBB4A* KO, the expression of E-cadherin increased, whereas the expressions of N-cadherin and vimentin decreased (Fig. 5E). Likewise, in DU145, PC3, and LNCaP cells, the transcription levels of β-catenin-targeted genes, *c-MYC, CCND1*, and *VIM*, were downregulated by *TUBB4A* KO or KD (Fig. S7D). These data indicate that TUBB4A interacts with MYH9 to activate GSK3β/β-catenin signaling, which contributes to TUBB4A-mediated tumorigenic properties.

### *TUBB4A* knockout retards xenograft tumor growth and metastasis of prostate cancer cells in vivo

**Fig. 6:**
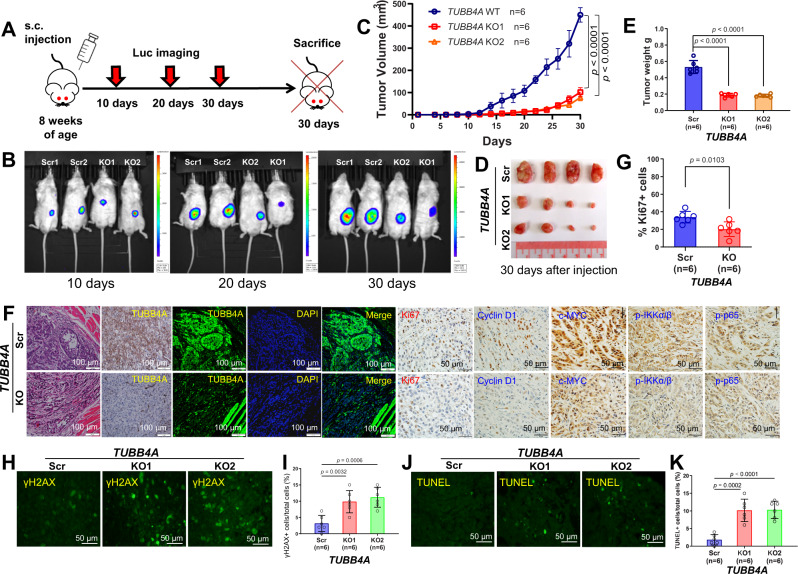
*TUBB4A* KO reduces xenograft tumor growth in NSG mice. **A** Schematic diagram of S.C. xenograft tumors in NSG mice followed up to 30 days. **B** Luciferase imaging of xenografts after implantation. **C** Tumor volumes after injection (*n* = 6/group). **D**, **E** Representative tumor masses and mean tumor weights on day 30. **F** H/E, IHC, and IF staining of xenograft tumors by specific antibodies to TUBB4A, Ki67, cyclin D1, c-MYC, p-IKKα/β, and p-p65. Scale bar, 50 μm or 100 μm. **G** Statistical analysis of Ki67+ cells for scrambled and KO groups. **H**, **I** IF staining and statistical analysis of xenograft tumors by the DNA damage response marker γH2AX. Scale bar, 50 μm. **J**, **K** IF staining and statistical analysis of xenograft tumors by the apoptotic marker, TUNEL. Scale bar, 50 μm. Data are replicated 6 times (**C**, **E**, **G**, **I**, **K**). Data are presented as the means and SD with a repeated measures ANOVA (**C**) or an ANOVA followed by Tukey’s post hoc *t* test (**E**, **I**, **K**) or a two-tailed *t* test (**G**). Source data are provided as a Source data file. Scr, scramble; KO, knockout; s.c., subcutaneous injection; luc, luciferase.

**Fig. 7:**
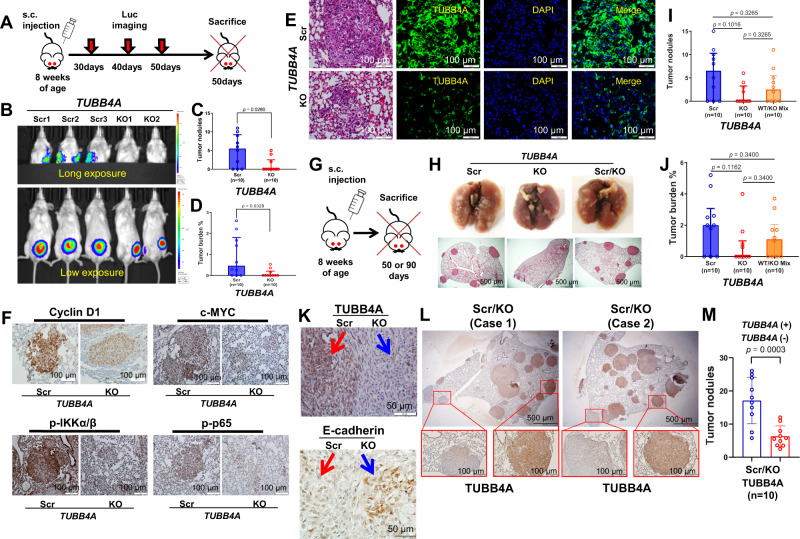
*TUBB4A* KO decreases tumor metastasis in the lungs of NSG mice. **A** Schematic diagram of S.C. xenograft tumors followed for up to 50 days in NSG mice. **B** Luciferase imaging of xenograft tumors and metastases after implantation. **C**, **D** Statistical analysis of tumor nodules and burdens of lung metastasis at day 50. **E** H/E and IF staining of metastatic tumors. Scale bar, 100 μm. **F** IHC staining of c-MYC, p-IKKα/β, and NF-κB (p-p65) of lung metastatic tumors in DU145 xenografts. Scale bar, 100 μm. **G** Schematic diagram of S.C. xenograft tumors followed for up to 90 days in NSG mice. **H** Representative tumor metastases in the lung at day 50. Scale bar, 500 μm. **I**, **J** Statistical analysis of tumor nodules and burdens of lung metastasis at day 50. **K** IHC staining of TUBB4A and E-cadherin in scrambled/KO mixed S.C. xenografts. Scale bar, 50 μm. **L** IHC staining of TUBB4A in scrambled/KO mixed lung metastatic tumors at day 90. Scale bar, 100 μm or 500 μm. **M** Statistical analysis of tumor nodules of lung metastasis at day 90. Data are replicated 10 times (**C**, **D**, **I**, **J**, **M**). Data are presented as the medians and interquartile ranges with a Mann–Whitney *U* (**C**, **D**) or Kruskal–Wallis followed by a Benjamini–Hochberg false discovery rate test for multiple comparisons (**I**, **J**). Data are presented as the means and SD with a two-tailed *t* test (**M**). Source data are provided as a Source data file. Scr, scramble; KO, knockout; s.c., subcutaneous injection; luc, luciferase.

To determine the effect of TUBB4A on tumor growth in mice, we subcutaneously (S.C.) injected scrambled or *TUBB4A* KO DU145-luciferase (luc) cells into immunodeficient NOD-scid IL2rg^null^ (NSG) male mice (Fig. 6A). Luc imaging analysis showed that, for up to 30 days after injection, xenograft tumor growth was slower in mice injected with *TUBB4A* KO cells than for mice injected with scrambled cells (Fig. 6B–E). Hematoxylin/eosin (H/E) and IHC analyses revealed more extensive cell death and expression of E-cadherin but lower expression of Ki67, cyclin D1, c-MYC, p-IKKα/β, p-p65, and vimentin in KO xenograft tumors compared to scrambled xenograft tumors (Figs. 6F, G and S9A). IHC and IF analyses confirmed *TUBB4A* KO in xenograft tumor cells (Fig. 7F). IF analysis validated more extensive DNA damage (γH2AX) and apoptosis (TUNEL) in KO xenograft tumors compared to scrambled xenograft tumors (Fig. 6H–K).

The dissemination of cancer cells from primary tumors and their subsequent seeding of tumor colonies in distant tissues involves a multi-step process known as the invasion-metastasis cascade^31^, including primary tumor growth, angiogenesis, migration and invasion, transport and dissemination, adhesion, extravasation, and colonization. To determine the role of TUBB4A in tumor metastasis, we extended our observation for up to 50 days after S.C. injection (Fig. 7A). Tumors in male mice (7/10) injected with scrambled cells progressed to distant metastases in the lung, but fewer mice (3/10) injected with KO cells had lung metastases (Fig. 7B). Likewise, tumor nodules and burdens in the lung were lower in mice injected with KO cells *versus* those injected with scrambled cells (Fig. 7C, D). *TUBB4A* KO in metastatic tumor cells of the lung was confirmed by IF analysis (Fig. 7E). Of note, IHC analysis showed lower expressions of cyclin D1, c-MYC, p-IKKα/β, p-p65, and vimentin but higher expression of E-cadherin in *TUBB4A* KO metastatic tumors compared to scrambled metastatic tumors (Figs. 7F and S9B). Likewise, IHC and IF analyses confirmed increased DNA damage (γH2AX) and apoptosis (TUNEL) in *TUBB4A* KO metastatic tumors compared to scrambled metastatic tumors (Figs. S9C–E).

To confirm the role of TUBB4A in lung metastasis, we injected scrambled, *TUBB4A* KO, or scrambled/KO-mixed DU145 cells S.C. into NSG male mice (Fig. 7G). At 50 days after injection, there were >3-fold more tumor nodules and burdens in the mice with injected scrambled cells *versus* KO cells, and ~2-fold more in the mice with scrambled/KO-mixed cells *versus* KO cells (Fig. 7H–J), although no statistical significance was observed. Of note, elevated expression of E-cadherin was evident in KO xenograft tumors compared to scrambled xenograft tumors (Fig. 7K, bottom panel). In mice injected with scrambled/*TUBB4A* KO mixed cells, at 90 days after injection, most lung metastases were derived from scrambled cells as compared to KO cells (Fig. 7L, M).

To substantiate the effect of TUBB4A on the tumor invasion-metastasis cascade and survival in the lung, we intravenously injected the scrambled and *TUBB4A* KO DU145 cells, respectively, into NSG male mice (Fig. S10A). At 30 days after injection, decreases of the tumor number and burden in the lung were evident in the mice with KO cells compared to scrambled cells (Figs. S10B–E). The *TUBB4A* KO in tumor cells of the lung was confirmed by IF analysis (Fig. S10F). Likewise, expression of γH2AX was increased in KO xenograft tumors compared to scrambled xenograft tumors, indicating more extensive DNA damage after *TUBB4A* KO (Fig. S10F). Thus, in the lung, TUBB4A may be necessary for cell colonization and survival, facilitating the formation of lung metastases.

### Prostate-specific deletion of *Tubb4a* delays tumor development and metastasis in spontaneous prostate cancer mouse models

**Fig. 8:**
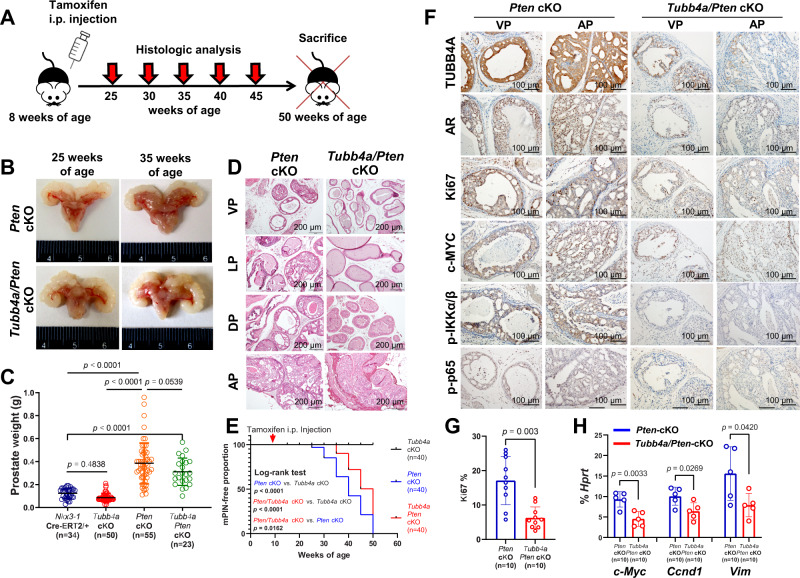
Prostate-specific deletions of *Tubb4a* and *Pten* and tumor progression in mouse prostate. **A** Schematic diagram of spontaneous developed prostate tumors followed for up to 50 weeks of age in genetically engineered mouse models. **B** Representative mouse prostates at 25 and 35 weeks of age. **C** Weights of prostates in the mice at 35 weeks of age. **D** Representative H/E staining of mouse prostates at 35 weeks of age. Scale bar, 200 µm. **E** Kaplan–Meier curves of mPIN incidences for up to 50 weeks of age. At 20, 25, 30, 35, 40, 45, and 50 weeks of age, 5 mice/per time point were sacrificed for pathologic analysis. **F** Representative immunostaining for TUBB4A, AR, Ki67, c-MYC, p-IKK, and p-p65 in the prostates of mice at 35 weeks of age. Scale bar, 100 µm. **G** The percentage of Ki67 cells as an indicator of proliferating cells among the mouse prostate or tumor tissues. **H** Relative mRNA levels of c-*Myc*, *Ccnd1*, and *Vim* genes as a percentage of *Hprt* expression in microdissected prostate epithelial cells as determined by qPCR at 35 weeks of age. Data are replicated 5 (**H**), 10 (**G**), 23–55 (**C**), and 40 (**E**) times. Data are presented as the means and SD with a two-tailed *t* test (**G**, **H**) or an ANOVA followed by Tukey’s post hoc *t* test (**C**). Source data are provided as a Source data file. AP, anterior prostate; DP, dorsal prostate; LP, lateral prostate; VP, ventral prostate; cKO, prostate conditional knockout; mPIN, mouse prostatic intraepithelial neoplasia; AR, androgen receptor; i.p., intraperitoneal injection.

Recent evidence shows an association between elevated β3-tubulin expression and *PTEN* deletions in prostate cancer, suggesting that changes in the levels of β3-tubulin result from PTEN-mediated genetic reprogramming^32^. To elucidate the role of *Tubb4a* in spontaneous prostate cancer models, we crossed *Nkx3-1*^*CreERT2*^ knock-in mice with *Tubb4a* floxed mice and/or *Pten* floxed mice to create prostate conditional *Tubb4a* single prostate conditional KO (cKO) (*Tubb4a-*cKO), *Pten* single cKO (*Pten-*cKO), and *Tubb4a* and *Pten* double cKOs (*Tubb4a/Pten-*cKO) mouse models on a C57BL/6 background (Figs. S11A–C). All mice were treated with tamoxifen at 8 weeks of age for induction of knock-in Cre (CreERT2) at the *Nkx3.1* locus in mouse prostate epithelial cells^33^. In *Tubb4a-*cKO mice, ~90% deletions of the *Tubb4a* locus were identified in microdissected prostate epithelial tissues at 8 weeks after tamoxifen injection (Fig. S11B). Thus, the deletion of *Tubb4a* was sufficient in the mouse prostate epithelial tissues. However, no *Tubb4a-*cKO mice developed prostate cancer, and no histologic changes were found in *Tubb4a-*cKO prostate versus *Nkx3-1*^*CreERT2/+*^ control prostate for up to 50 weeks of age. Approximately 100% of *Pten-*cKO mice developed high-grade mouse prostatic neoplasia hyperplasia (mPIN) but no carcinoma and metastasis for up to 12 months of age^33^. Furthermore, compared to *Pten-*cKO mice, prostate size and weight were not changed at 25 weeks of age but reduced at 35 weeks of age in *Tubb4a/Pten-*cKO mice (Fig. 8A–C), suggesting that *Tubb4a/Pten-*cKO mice experienced a slower prostate growth than *Pten-*cKO mice. Histologic assessment revealed delayed formation of mPIN in *Tubb4a/Pten-*cKO *versus Pten-*cKO mice, but no evidence of invasion through the basement membrane was evident in mice for up to 50 weeks of age. (Fig. 8D, E). Further, for *Pten/Tubb4a* cKO mice, we assessed the incidence of mPIN lesions in lobes of ventral prostate (VP), lateral prostate (LP), dorsal prostate (DP), and anterior prostate (AP) using the mPIN-free Kaplan–Meier analysis (Fig. S11D). The incidence of mPIN lesions in VP and DP lobes was more delayed than in LP and AP lobes, but the result was not statistically significant (*p* = 0.079, Fig. S11D). In addition, mPIN lesions in all mice were AR-positive, but AR expression was reduced after *Tubb4a* cKO (Fig. 8F). However, less expressions of Ki67^+^, c-MYC, p-IKKα/β, and p-p65 were present in *Tubb4a/Pten-*cKO *versus Pten-*cKO mPIN lesions (Fig. 8F, G). Since expressions of c-MYC, cyclin D1, and vimentin were reduced after *TUBB4A* KO (Figs. 5E and S7D), we analyzed the mRNA expression of β-catenin*-*targeted genes c-*Myc*, *Ccnd1*, and *Vim* in microdissected tumor cells. As shown in Fig. 8H, expression levels of c-*Myc*, *Ccnd1*, and *Vim* were lower in *Tubb4a/Pten-*cKO *versus Pten-*cKO tumor cells, supporting indirect transcription regulation of these β-catenin targeted genes by TUBB4A.

**Fig. 9:**
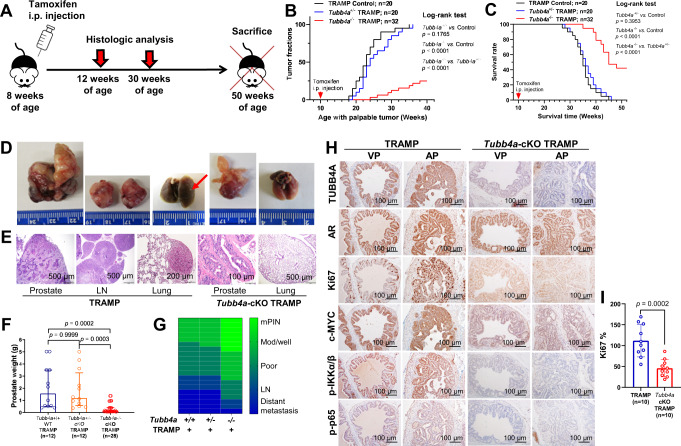
Prostate-specific deletions of *Tubb4a* and prostate tumor progression and metastasis in TRAMP models. **A** Schematic diagram of spontaneously developed prostate tumors followed for up to 50 weeks of age in genetically engineered mouse models. **B**, **C** Kaplan–Meier curves of palpable prostate tumors and survival for up to 40 and 50 weeks of age, respectively. **D**, **E** Representative prostate tumor growth and metastasis and H/E staining at 50 weeks of age. Scale bar, 100 μm, 200 μm, or 500 μm. **F** Weights of prostates at 30 weeks of age. **G** Heatmap of prostate tumor progression and metastasis at 30 weeks of age. **H** Representative immunostaining for TUBB4A, AR, c-MYC, p-IKK, and p-p65 in the prostates of mice at 30 weeks of age. Scale bar, 100 µm. **I** Statistical analysis of Ki67+ cells for TRAMP and *Tubb4a*-cKO TRAMP groups. Data are replicated 10 (**I**), 20–32 (**B**, **C**), and 12–28 (**F**) times. Data are presented as the medians and interquartile ranges with a Kruskal–Wallis followed by a Dunn’s post hoc test (**F**). Data are presented as the means and SD with a two-tailed *t* test (**I**). Source data are provided as a Source data file. AP, anterior prostate; VP, ventral prostate; cKO, prostate conditional knockout; mPIN, mouse prostatic intraepithelial neoplasia; Mod/well, moderately/well-differentiation grades; LN, lymph node; AR, androgen receptor; i.p., intraperitoneal injection; LN, lymph nodes.

To determine the role of *Tubb4a* in spontaneous tumor metastasis, we crossed *Tubb4a* cKO alleles into transgenic adenocarcinoma of the mouse prostate (TRAMP) mice on a C57BL/6 background (Figs. S11E). TRAMP mice express *SV40* large T antigen, a potent oncogene, under the control of a prostate-specific rat probasin (PB) promoter^34^, and develop spontaneous prostate cancers with tumor metastasis^34^. As shown in Fig. 9A–E, the onset of prostate tumor and mouse death were delayed in homozygous *Tubb4a-*cKO (also *Tubb4a*-cKO) TRAMP mice compared to heterozygous *Tubb4a-*cKO or *Tubb4a* wild-type (WT) TRAMP mice. Likewise, at 30 weeks of age, prostate weights were decreased in homozygous *Tubb4a-*cKO mice versus heterozygous *Tubb4a-*cKO or *Tubb4a* WT TRAMP mice (Fig. 9F). The TRAMP mice developed lymphatic and lung metastases starting at 25 weeks of age^35^. As summarized in Fig. 9G, most cancers in *Tubb4a* WT or heterozygous *Tubb4a-*cKO TRAMP mice had progressed to poorly differentiated carcinomas (14~15/20) and lymphatic (8/20) and lung (5/20) metastases at 30 weeks of age. In contrast, the homozygous *Tubb4a-*cKO TRAMP mice developed poorly differentiated carcinomas (7/20), and few mice had lymphatic (3/20) and lung (1/20) metastases. At 35 weeks, IHC analysis showed AR^+^ prostate tumors in all mice, but expressions of Ki67^+^, c-MYC, p-IKKα/β, and p-p65 were lower in *Tubb4a*-cKO TRAMP *versus* WT TRAMP prostate tumors (Fig. 9H, I). Of note, at 12 weeks of age, low expressions of c-MYC, p-IKKα/β, and p-p65 were evident in the pre-malignant stage of tumor initiation (Fig. S12A). CK5 is a prostate basal cell marker, and its absence of basal cells supports the diagnosis of human prostate cancer^36^. Our IHC analysis showed a reduced expression of CK5 in the *Tubb4a/Pten-*cKO *versus Pten-*cKO mPIN lesions (Fig. S12B) but no change between *Tubb4a*-cKO TRAMP and WT TRAMP prostate tumors (Fig. S12C). These data support that prostate-specific inactivation of both alleles of *Tubb4a* reduces the malignancy of prostate cancer and alleviates tumor progression and metastasis, which may be involved in regulation of NF-κB and/or β-catenin/c-MYC signaling.

## Discussion

In the present study, we identified, in human prostate cancers, tumor-specific high expression of *TUBB4A* associated with poor overall survival. Further, there was, in prostate cancers, a negative correlation between the expression and promoter DNA methylation of *TUBB4A*, suggesting that DNA demethylation contributes to the expression of *TUBB4A*. *TUBB4A* KO reduced cell growth and migration, but the ectopic expression of *TUBB4A* rescued this phenotype. Functional analyses suggested that, during constricted migration of prostate cancer cells, *TUBB4A* KO changed the actin cytoskeleton, induced DNA damage, and disrupted the NF-κB signaling response and activation. Of note, during constricted cell migration, TUBB4A interacted with MYH9 to protect the nucleus and cell shape and cytokinesis. Functional analyses revealed that TUBB4A/GSK3β bind to the N-terminal of MYH9, and that *TUBB4A* KO reduces MYH9-mediated GSK3β ubiquitination and degradation, leading to decreased activation of GSK3β/β-catenin signaling and the EMT. These data suggest that, in prostate cancer cells, MYH9-induced GSK3β/β-catenin signaling contributes to TUBB4A-mediated tumorigenic properties. In addition, in xenograft mouse models, *TUBB4A* KO retarded tumor growth and metastasis of prostate cancers. In spontaneous prostate cancer mouse models, prostate-specific deletion of *Tubb4a* delayed tumor growth and metastasis. Of note, increased DNA damage but decreased NF-κB, cyclin D1, and c-MYC signaling activation were evident in prostate tumors with a *TUBB4A* defect. These data support an oncogenic role of *TUBB4A* in growth and metastasis of prostate cancer cells.

*TUBB4A* is amplified in 14% of neuroendocrine prostate cancers and 8% of castration-resistant prostate cancers but not in primary prostate adenocarcinomas, suggesting that genetic alteration of *TUBB4A* contributes to development of neuroendocrine and castration-resistant prostate cancers. Overexpression of TUBB4A was evident in last-stage and metastatic prostate adenocarcinomas and was associated with poor patient survival, but there was no genetic alteration of *TUBB4A* in most of these cancers, suggesting that epigenetic regulation of *TUBB4A*, such as DNA demethylation, may contribute to its overexpression. In particular, expression of TUBB4A was higher in prostate adenocarcinomas of AAs than those of EAs, suggesting an ancestral difference of TUBB4A expression for patients with prostate cancer. Since the prostate cancer mortality is high for AA men (4.0%) compared with EA men (2.2%)^37^, TUBB4A may be associated with the mortality disparity for patients with prostate cancer. However, further studies with additional, independent sample cohorts are required for validation.

For cell migration, the movement and positioning of the nucleus and nucleus–cytoskeleton interactions are required^38^. During cell migration and invasion, cells pass through barriers, such as the ECM or neighboring cells. For cells to pass through these pores, their plasma membrane, cytoplasm, and small organelles are easily adjustable, but the nucleus, due to its size and stiffness, is the main restricting component^39,40^. During migration, cytoskeleton components such as microtubules may be involved in nuclear positioning, but the mechanism remains to be elucidated. Although microtubule dynamics appear to be dispensable for nuclear movement in two-dimensional (2D) cell migration^41^, nuclear movement with multiple confinements may be associated with microtubule dynamics in 3D cell migration^42,43^. Of note, the size and stiffness of the nucleus constitute a limitation for 3D cell migration. Live imaging of migrating cells through tight spaces shows that nuclei are pushed, pulled, and deformed to progress^39,40^. Nuclear lamina-cytoskeleton interactions regulate nuclear movement. Perinuclear Arp2/3-driven actin polymerization enables nuclear deformation, and actin nucleation promotes lamin A/C perturbation, facilitating nuclear deformability and increasing the capacity of cells to migrate through narrow constrictions^44^. Actin aggregates on the surface of the nuclear membrane to drive deformation of the nucleus, which helps it squeeze through micropores in the TME^44^. In the present study, in 3D cell migration, TUBB4A aggregated around the nucleus, whereas *TUBB4A* KO resulted in nuclear DNA damage^45^. Further, TUBB4A physically interacted with MYH9 in the cytoplasm. When the nucleus led the way during 3D migration, TUBB4A and MYH9 aggregated together at the forefront of the nucleus. MYH9, also known as heavy chain of non-muscle myosin IIA, is a widely expressed cytoplasmic myosin that participates in a variety of processes requiring translocation of the actin cytoskeleton^25^. However, during migration, there was no effect of *TUBB4A* KO on cytosol α-tubulin and nuclear lamina. These data suggest that, during migration of prostate cancer cells, TUBB4A interacts with MYH9 and actin to protect the nucleus and enhance cell survival.

Microtubules influence homeostatic mechanisms and cell stress responses by regulating intracellular trafficking through cytoskeletal remodeling and modulating the induction of cell death pathways^46^. Microtubules regulate MAPK signaling, including the ERK and p38 families^47,48^. Tubulins and microtubule-associated proteins may be involved in a range of cellular stress responses, thus conferring a survival advantage to cancer cells^19^. In the present study, *TUBB4A* KO did not change the phosphorylations of MEK, ERK, and p38MAPK, but, during constricted cell migration, it induced severe DNA damage and disrupted the NF-κB signaling response and activation. Our data suggest that, during migration of prostate cancer cells, TUBB4A confers a survival advantage through protection of the nucleus and an NF-κB signaling response to DNA damage. Furthermore, tubulins interact with microtubule-associated proteins, such as microtubule-associated protein tau (MAPT)^49^ and microtubule-associated protein 2 (MAP2)^50^, to influence microtubule stability and dynamics, chemotherapy sensitivity, and growth of various cancers^51^. A recent study identified an interaction of TUBB4A with GLUT1 (Glucose transporter 1). In human glioblastomas, silencing of *TUBB4*A or *GLUT1* reduces glioblastoma stem cell tumor sphere formation, self-renewal, and proliferation^52^. In the present study of prostate cancer cells, TUBB4A directly interacted with MYH9 and reduced GSK3β ubiquitination and degradation. Either *TUBB4A* KO or *MYH9* KD activated GSK3β/β-catenin signaling through increased expression of GSK3β and decreased expression of nuclear β-catenin and its downstream targets, cyclin D1 and c-MYC, as well as inhibition of the EMT. These data suggest that interaction of TUBB4A/MYH9 is not only necessary for protection of the nucleus during migration of prostate cancer cells, but also for inducing activation of GSK3β/β-catenin signaling and subsequently influencing tumor growth and metastasis. Thus, the functional consequence of TUBB4A/MYH9 interaction in prostate cancer cells may be through two potential mechanisms. (1) A TUBB4A-mediated mechanical mechanism by direct physical crowding of cytoskeletal proteins TUBB4A and MYH9 on the nuclear membrane to protect the nucleus, prevent DNA damage, and maintain an NF-kB signaling response during constricted cell migration. (2) A TUBB4A-mediated molecular mechanism through interaction with TUBB4A/MYH9 to regulate GSK3β/β-catenin signaling, contributing to TUBB4A-mediated tumor growth and metastasis. However, more comprehensive characterization of the TUBB4A-mediated networks is a necessary part of future studies.

In summary, in prostate cancer cells, TUBB4A facilitates tumor growth and metastasis through MYH9-mediated GSK3β/β-catenin signaling. During the metastatic process, interaction of TUBB4A with MYH9 may protect the nucleus to avoid DNA damage and maintain tumor cell survival through the NF-κB signaling response and activation. TUBB4A expression is a potential risk factor for prostate cancers, especially for AA prostate cancers, castration-resistant prostate cancers, and neuroendocrine prostate cancers. Our results address the role of TUBB4A in prostate cancer progression and provide a therapeutic target for patients who have tumors with TUBB4A overexpression, leading to the development of new targeted therapies of aggressive prostate cancer.

## Methods

### Cell lines, plasmids, antibodies, and experimental reagents

Prostate cancer cell lines PC3 (Cat No. CRL-1435), DU145 (Cat No. HTB-81), and LNCaP (Cat No. CRL-1740) were purchased from the ATCC (USA). DU145-luc cells (Cat No. A1700149) were acquired from the JCRB Cell Bank (Japan). Cells freshly amplified and frozen after obtention from the ATCC and JCRB were used every 5 months. The cell lines were professionally verified and free from contamination. Short tandem-repeat analysis for DNA fingerprinting was also used to verify the cell lines. Cells were cultured for less than 6 months in Dulbecco′s modified Eagle′s medium (DMEM) supplemented with 10% FBS (Thermo Fisher Scientific, USA). The specific primary antibodies used in all experiments are as shown in Table S3. The PX458 vector was obtained from Addgene (USA), and the p3XFLAG-CMV-7.1 vector was acquired from Millipore-Sigma (USA). *TUBB4A* sgRNAs and primers were synthesized by Integrated DNA Technologies (IDT, USA). *TUBB4A* and *MYH9* Dicer-substrate siRNAs (DsiRNAs, IDT) (Table S4) were transfected into cells. Tamoxifen (Millipore-Sigma, USA) was used for induction of Cre recombinase activity in mice.

### Generation of *TUBB4A* KO and rescued cell lines

CRISPR/Cas9 gene editing was performed on the exon 2 region of *TUBB4A*. sgRNAs were designed using the Benchling’s CRISPR tool (https://www.benchling.com/crispr/) and obtained based on suitable scores. Oligos pairs of sgRNAs were denatured, annealed together, and inserted into the PX458-GFP vector following previously published protocols^53^. pX458-GFP plasmids, which contain each of their own sgRNA-targeted sequences or no sgRNA for a scramble control, were transfected into cells with Lipofectamine 3000 (Thermo Fisher Scientific). After GFP was sorted by flow cytometry, 100 GFP^+^ cells were placed into a petri dish. Scrambled control or *TUBB4A* KO colonies were identified by Sanger DNA sequencing after selection, and validated by Western blots. All sgRNAs were checked using Cas-OFFinder tool (Daejeon, South Korea)^18^, and potential off-target regions were predicted for PCR analysis to test possible off-targets^54^. All selected colonies of scramble and KO cells were validated by PCR and Sanger sequence analysis to exclude off-target effects in potential off-target regions of sgRNAs, as described previously^54,55^. The sgRNAs and primers for CRISPR design are shown in Table S4. To rescue TUBB4A expression in *TUBB4A* KO cells, the CMV-7.1 vector was designed to encode with the open reading frame of *TUBB4A* as a rescue plasmid. Then, the plasmid was transfected into confirmed *TUBB4A* KO cell lines, according to the method described above, and the rescued expression of *TUBB4A* was validated by Western blots. The sequence of the *TUBB4A* open reading frame is shown Table S4.

### Cell growth assay and colony formation assay

Cells were cultured in 6-well plates until 70% confluent in DMEM with 10% FBS. After washing twice with phosphate-buffered saline (PBS), cells were digested, centrifuged, and seeded (5000 cells each well) into 24-well plates for assessment of cell growth and into 6-well plates (400 cells each well) for assay of colony formation. For assessment of cell growth, cells were counted at days 1, 2, 3, 4, 6, and 8, and cell growth curves were prepared. The growth of PC3 and DU145 cells was measured by live cell kinetic imaging with walk-away automation using a Lionheart™ FX cell imager (Agilent Technologies BioTek, Winooski, VT) according to the manufacturer’s protocol. For assay of colony formation, after cell culture for two weeks, cells were cleaned 3 times and fixed by 4% paraformaldehyde (PFA) for 20 min. After washing with PBS 3 times, cells were stained by violet crystal (0.1 g/mL) for 20 min. The plates were scanned to determine the colony sizes and numbers. A colony was considered to be 50 cells or more as determined microscopically^56^. For soft-agar colony formation assay, 10^5^ cells in each well were plated on top of a solidified layer in culture medium, which was changed every three days. After 3–4 weeks, colonies were cleaned with PBS and photographed with a digital camera (Thermo Fisher Scientific). 3D colonies were counted based on the capacity of single cells to grow to colonies consisting of at least 50 cells^56,57^.

### Transwell migration assays and automated scratch migration assays

DMEM with 10% FBS was used to culture cells. The cells were suspended in DMEM with 0.2% FBS and seeded at 5 × 10^4^ per well into 8-μm invasion chambers (Millipore-Sigma). After 20 h, chambers were washed 3 times, and cells on the other side of the chambers were scraped off. Then, migrant cells were fixed with 4% PFA, washed with PBS, stained with 4′,6-diamidino-2-phenylindole (DAPI) or hematoxylin for 10 min, dried away from light, and photographed with a microscope. In addition, the growth of DU145 cells as determined by an automated scratch migration assay was measured by live-cell kinetic imaging with walk-away automation using Lionheart™ FX cell imager (Agilent Technologies BioTek, Winooski, VT) according to the manufacturer’s protocol.

### Collagen 2D/3D cell migration assay

For 2D collagen-coating of cell culture plates, 50 µg/mL rat tail collagen type I (Millipore-Sigma) was added to the coverslips and incubated for 1 h at 37 °C, followed by washing 3 times with PBS before plating cells. For time-lapse videos, 2D collagen matrix was made to treat 12-well plates. Under the microscope, 1000 cells were counted per well. In order to concentrate the cells, a sterilized hollow glass cylinder was placed in the middle of the hole. The cells were laid in the cylinder and cultured in an incubator for 6 h until the cells adhered to the plate wall. Medium was discarded until 90% of the cells had adhered to the wall. Then, fresh complete medium was added after washing twice with PBS to remove impurities and non-adherent cells. Plates (12-hole) were placed into an intelligent full-automatic fluorescence imaging microscope (EVOS AUTOFL2, Thermo Fisher Scientific) connected with CO_2_ and set at 37 °C, and the field of vision (20×) was set through an Automatic Imaging System. Area with more cells was selected to take pictures, every 30 min from the first hole, and circulating for 24 h. All pictures were set to compose video automatically.

For 3D cell migration assay, the 3D collagen gel polymerization method was utilized according to the following protocol. Briefly, rat tail collagen type I was diluted to 3 mg/mL in DMEM with 5 µg/mL fibronectin and FBS-free, and pH was adjusted to 7.4 using 1 N NaOH solution. Next, 200 µL of collagen was added to 8-well chamber slides, and, for experiments in which collagen gel porosity was manipulated, collagen was polymerized at 37 °C for 36 h, and then 2000 cells were seeded on top of the gel and allowed to migrate for 20 h in an incubator box. After that, the live and dead cells and DNA damage were observed.

### 2D/3D immunofluorescence (IF)

For 2D IF of cells, 10^4^ cells were seeded into 8-well chamber slides and cultured for 20 h. First, cells were washed with PBS and fixed by 4% PFA, then 0.25% Triton X-100 was used to permeabilize cells for 10 min. After that, cells were blocked with PBS containing 2% goat serum for 1 h, and a primary antibody was incubated at 4 °C overnight. Finally, secondary antibodies were added for 1 h, and DAPI staining was performed for 10 min. After washing, coverslips were mounted on glass slides. With collagen gels, 3D IF experiments were performed with minor modifications. Briefly, cells in collagen gels were fixed for 30 min with 4% PFA, washed once with Tris Buffer, permeabilized for 35 min using 0.5% Triton X-100m and washed for 30 min with 0.1 M glycine followed by washing with 0.1% TBST (Tris-buffered saline, 0.1% Tween 20). Fixed cells were blocked for at least 6 h to overnight in a solution containing 2% BSA, 0.1% Tween-20, 2% goat serum, and incubated overnight with primary antibody, washed with TBST, and incubated at least 5 h in fluorescently conjugated secondary antibody with DAPI followed by washing 3 times with TBST, then 3 washes with TBS without Triton. Collagen gels were then mounted with fluorescent mounting media (Agilent, USA).

### Western blots and immunocoprecipitation

Western blotting experiments were performed as previously^58,59^. The blots were imaged with the ChemiDoc MP Imaging System (Bio-Rad, Hercules, CA) or Mini Med 90 Processor (AFP, Peachtree City, GA). Immunocoprecipitation experiments were conducted with minor modifications. Cells were lysed in a cooled buffer solution containing protease inhibitors and phenylmethylsulfonyl fluoride (Millipore-Sigma) for 15 min, then extracted proteins were assigned to different tubes and incubated with IgG and a specific antibody for one day at room temperature. Finally, antibody protein complexes were precipitated with protein A/G agarose (Thermo Fisher Scientific).

### High-resolution mass spectrometry (MS) analysis

The samples immunoprecipitated with TUBB4A antibody (ab222822, 1:200) and input control samples were used for agarose gel electrophoresis. Afterward, the gel was put into 500 mL Coomassie Blue solution and shaken for 20 h at 37 °C. The Coomassie-stained and excised bands were de-stained in a solution containing 100 mM ammonium bicarbonate and acetonitrile and were equally divided for overnight. Dithiothreitol (25 mM) was used to reduce the disulfide bonds at 50 °C for 30 min, and then alkylation of free thiols with iodoacetamide (55 mM) was accomplished in the dark for 30 min at 37 °C. The gel pieces were evaporated to dryness after removal of excess alkylating agents, placed in 100 mM ammonium bicarbonate buffer, and further digested overnight with trypsin. The peptides were then extracted and evaporated to dryness. For mass spectrometry analysis, the samples were suspended on 0.1% formic acid in ddH_2_O.

An aliquot (5 µL) of each digest was loaded onto a Nano cHiPLC (Eksigent, USA) at the speed of 2 µL/min using an Eksigent 415 LC system autosampler. After the cartridge was washed for 10 min, the bound peptides were rinsed onto a Nano cHiPLC column for 35 min at 1000 nL/min. The column was washed for 5 min and re-balanced for an additional 10 min. A SCIEX 5600 Triple-Tof mass spectrometer (Sciex, Canada) with ion spray voltage of 2300 V and declustering potential of 80 V was used to analyze the protein digestion products, and ion spray and curtain gases were set separately at 10 psi and 25 psi. Time-of-flight survey scan with *m*/*z* value from 400 to 1250 implemented eluted peptides test to detect the most intense 20 ions during processing. To obtain the tandem mass spectra, production time-of-flight scans of 50 ms were accomplished, and spectra were then de-isotoped and centroided by Analyst software. A β-galactosidase trypsin digest was performed to find and verify the precision. Tandem MS data processing provides protein identifications using a Protein Pilot 4.5 search engine and a trypsin/chymotrypsin co-digestion parameter. Significant proteins were selected on the criteria of having at least two peptides detected with a confidence score of at least 95% using the Paradigm method. Data are available via ProteomeXchange with identifier PXD033359.

### Transplantation of xenogeneic tumor cells

NOD-scid IL2rg^null^ (NSG) immunodeficient mice were purchased from the Jackson Laboratory (USA). Scrambled or *TUBB4A* KO DU145-luc cells (10^6^ cells in 100 µL) were S.C. injected into the left flanks of NSG male mice at 8 weeks of age. Xenograft tumor growth and metastasis were monitored by firefly luciferase bioluminescence imaging every 10 days up to 50 or 90 days. Tumor sizes and weights were recorded as described^60,61^. Mice were euthanized for histopathological examination and further analysis. Next, scrambled and *TUBB4A* KO DU145-luc cells were mixed together (100 µL, 10^6^) and injected into the dorsal abdomen of NSG male mice at 8 weeks of age. Tumors and lung tissues were removed for histologic examination and expression analysis. Scrambled or *TUBB4A* KO DU145-luc cells (5 × 10^4^ cells in 100 µL) were injected into NSG male mice at 8 weeks of age via their tail veins. Mice were observed up to 30 days after injection, and metastatic sites were checked by histologic analysis. The numbers of surface lesions over all lobes of the lungs were scored before pathologic analysis. Tumor burden in the lungs was quantified in two-step sections from each lobe in a blinded fashion by calculating the area of tumor tissue as a percentage of the total tissue area^62^.

### Genetically engineered animal models

*Tubb4a* and *Pten* floxed mice (The Jackson Laboratory) were crossed to *Nkx3-1*^*CreERT2*^ knock-in mice (National Cancer Institute Mouse Model Deposit) expressing *Cre* cDNA under inducible control of the tamoxifen on a C57BL/6 background. We generated prostate *Tubb4a, Pten*, or both conditional KO (cKO, *Nkx3-1*^*CreERT2/-*^ × *Tubb4a*^flox/flox^, *Nkx3-1*^*CreERT2/-*^ × *Pten*^flox/flox^, *Nkx3-1*^*CreERT2/-*^ × *Tubb4a*^flox/flox^ × *Pten*^flox/flox^) male mice. Next, *Nkx3-1*^*CreERT2/-*^ × *Tubb4a*^flox/flox^ mice were crossed to TRAMP mice (The Jackson Laboratory) on a C57BL/6 background. We generated prostate *Tubb4a* cKO TRAMP male mice (*Nkx3-1*^*CreERT2/-*^ × *Tubb4a*^flox/flox^ × TRAMP) male mice. All mice were observed for tumor development and metastasis of spontaneous prostate cancers up to 1 year. Histologic examination and expression analysis were as described previously. Mice were housed in the University of Alabama at Birmingham Animal Resources Program with 12-h light/dark cycles, 65–75 degrees Fahrenheit, 40–60% humidity, and on-site veterinarian care. All animal experiments were conducted in accordance with accepted standards of animal care and approved by the Institutional Animal Care and Use Committee of University of Alabama at Birmingham (Protocol Number: IACUC-22241).

### Human subjects

Formalin-fixed, paraffin-embedded specimens of 136 primary prostate cancer tissues and 50 tumor-adjacent normal prostate samples were used for IHC staining. The tumor specimens contained the following information: patient age, race, prostate-specific antigen levels, and pathological stage. Samples from this study were collected from patients with prostate cancer who underwent primary surgery between January 2012 and June 2018 at the University of Alabama at Birmingham as presented in Table S1. All patients were diagnosed with prostate cancer by histopathological examination. The pathological staging was obtained using the tumor-node-metastasis (TNM) staging system, and pathologic grading was based on specimens corresponding to Gleason scores of 2–6, 7, and 8–10, respectively. Written informed consent was obtained from all subjects, and the study protocol was approved by the Institutional Review Board of the University of Alabama at Birmingham prior to start of this study. (Protocol Number: IRB-300006570)

### Immunohistochemical (IHC) analysis

IHC staining was performed using Vectastain Elite ABC kits (Vector Lab, USA) following the experimental procedures described previously^63,64^. The tumor cell positive rate (0% to 100%) per slice was multiplied by the dominant intensity pattern of staining (1: weak; 2: mild; 3: strong), and the overall H-scores ranged from 0 to 300. Image processing was performed using Adobe Photoshop CS2 (version 9.0.2; San Jose, CA).

### Construction and enrichment analysis of gene network and pathway

Gene network analysis was preformed using the HAPPI2 database^65^ with Protein-Protein Interaction (PPI) quality no less than 5-star. The highly confident shared interactors of MYH9 and TUBB4A were identified in the HAPPI2 database, which is one of the most comprehensive PPI databases. To investigate the pathways involving MYH9 and TUBB4A, pathway enrichment analysis was performed using NCI pathways collected from PAGER2^26^. MYH9, and TUBB4A and all shared interactors generated above as candidate genes were added in the input. The gene network and enriched pathways of the interactions between the genes were generated using PAGER2. The adjusted *p*-value by false discovery rate (FDR) was set to 0.05.

### Statistics and reproducibility

A Mann–Whitney *U* test or a two-tailed *t* test were used to analyze differences between two groups. The overall differences were analyzed with one-way ANOVA or Kruskal–Wallis test, followed by post-hoc assessments for more than two groups and a two-way repeated measures ANOVA test groups over time. The log-rank test was used to evaluate Kaplan–Meier survival curves and to analyze initiation and metastasis of tumors and survival of mice. Two-tailed tests were performed for all statistical analyses. For in vitro and in vivo analyses, experiments were repeated independently three and two times, respectively, with similar results to demonstrate reproducibility. Microsoft Excel (Version 16, USA), IBM SPSS (Version 25, USA), SAS (Version 9.4, USA), and GraphPad Prism (Version 8.4.3, USA) software were used to analyze all the data.

### Reporting summary

Further information on research design is available in the Nature Research Reporting Summary linked to this article.

## Supplementary information

Supplementary Information

Description of Additional Supplementary Information

Supplementary Video 1

Supplementary Video 2

Supplementary Video 3

Supplementary Video 4

Supplementary Video 5

Supplementary Video 6

Supplementary Video 7

Reporting Summary

## Data Availability

The authors declare that the data supporting the findings of this study are available within the manuscript and its supplementary information files. Genetic analysis of 6 studies, including TCGA dataset (www.cancer.gov/about-nci/organization/ccg/research/structural-genomics/tcga), was performed using cBioPortal^66,67^. Analysis of RNA expression and DNA methylation from TCGA dataset was performed using UALCAN^68^ and Wanderer^69^. Survival analysis with gene expression from TCGA dataset was performed using GEPIA^70^. The mass spectrometry proteomics data have been deposited to the ProteomeXchange Consortium via the PRIDE^71^ partner repository (www.ebi.ac.uk/pride/) with the dataset identifier PXD033359. Source data are provided with this paper.

## Source data

Source Data
