# Supplementary material for: TUBB4A interacts with MYH9 to protect the nucleus during cell migration and promotes prostate cancer via GSK3β/β-catenin signalling
Source: Nat Commun. 2022 May 19;13:2792. doi: 10.1038/s41467-022-30409-1 (PMC9120517; doi:10.1038/s41467-022-30409-1)

## Supplementary Figures

# TUBB4A interacts with MYH9 to protect the nucleus during cell migration and promotes prostate cancer via GSK3β/β-catenin signalling

Song Gao, Shuaibin Wang, Zhiying Zhao, Chao Zhang, Zhicao Liu, Ping Ye, Zhifang Xu, Baozhu Yi, Kai Jiao, Gurudatta A. Naik, Shi Wei, Soroush Rais-Bahrami, Sejong Bae, Wei-Hsiung Yang, Guru Sonpavde, Runhua Liu and Lizhong Wang

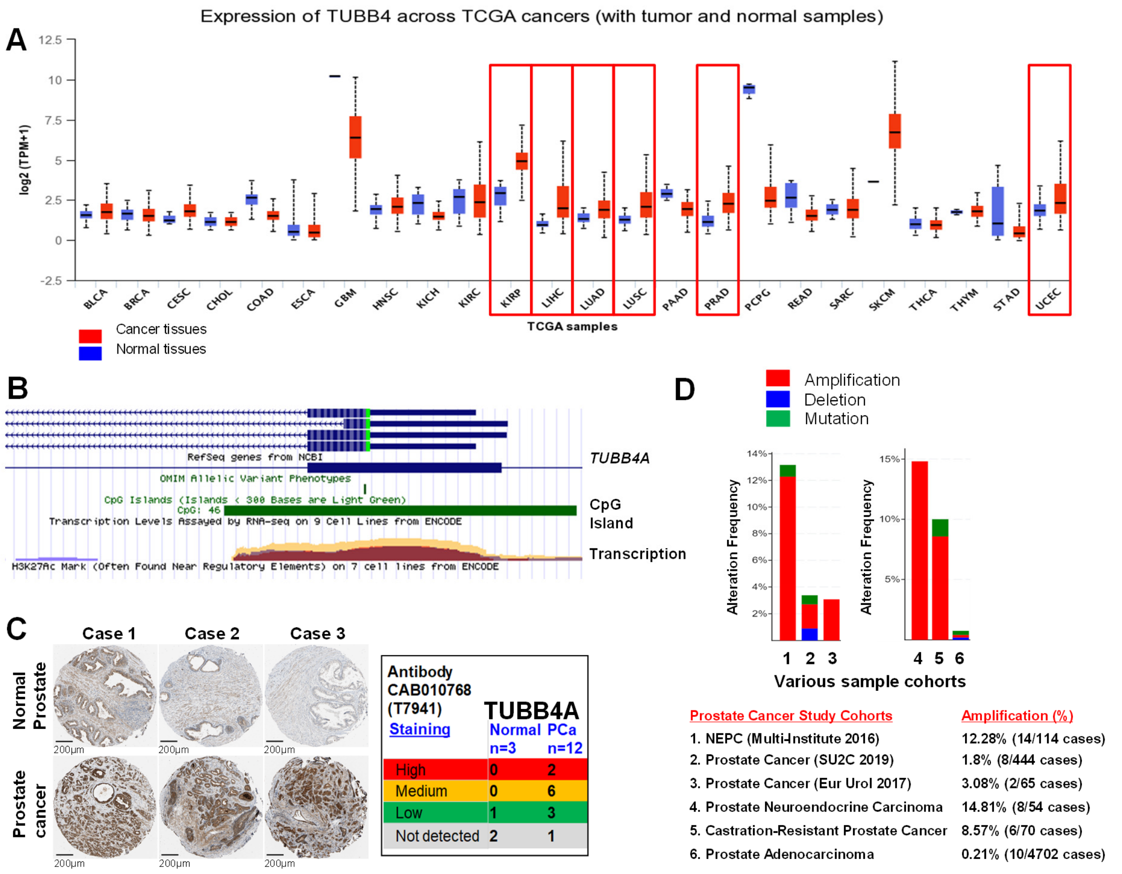


**Figure S1. Characterization of *TUBB4A* expression profiling and genetic alteration in human primary prostate cancer. A,** Expression of *TUBB4A* across tumors with normal control samples from TCGA dataset was analyzed using UALCAN (ualcan.path.uab.edu). Data are presented as the medians and interquartile ranges Red frames indicate high expression of *TUBB4A* in tumors as compared to normal controls. **B,** CpG Island in promoter and transcription of human *TUBB4A* gene. Screenshot from the UCSC genome browser showing location of the *TUBB4A* promoter, CpG island, exon1, intron1, and transcription (Broad Institute Histone data). **C**, Representative IHC staining and summary of TUBB4A intensity patterns in normal prostate and prostate cancer tissues (Uhlén M *et al.*, Mol Cell Proteomics, 2005

PubMed: 16127175) from The Human Protein Atlas (THPA, www.proteinatlas.org). Anti-human TUBB4A rabbit Ab (Millipore-Sigma, T7941) was used for IHC staining. **D**, Genetic alterations of *TUBB4A* in primary prostate cancers. Public dataset-based analysis and summary of genetic alterations (amplification, deletion, and mutation) of *TUBB4A* in primary prostate cancers from 6 studies using cBioPortal (www.cbioportal.org) with various public datasets. BLCA, bladder carcinoma; BRCA, breast invasive carcinoma; CESC, cervical squamous cell carcinoma; CHOL, cholangiocarcinoma; COAD, colon adenocarcinoma; ESCA, esophageal carcinoma; HNSC, head and neck squamous cell carcinoma; KIRC, kidney renal clear cell carcinoma; KIRP, kidney renal papillary cell carcinoma; LIHC, liver hepatocellular carcinoma; LUAD, lung adenocarcinoma; LUSC, lung squamous cell carcinoma; PAAD, pancreatic adenocarcinoma; PCPG, pheochromocytoma and paraganglioma; PRAD, prostate adenocarcinoma; READ, rectal adenocarcinoma; SKCM, skin cutaneous melanoma; STAD, stomach adenocarcinoma; THCA, thyroid carcinoma; UCEC, uterine corpus endometrial carcinoma.

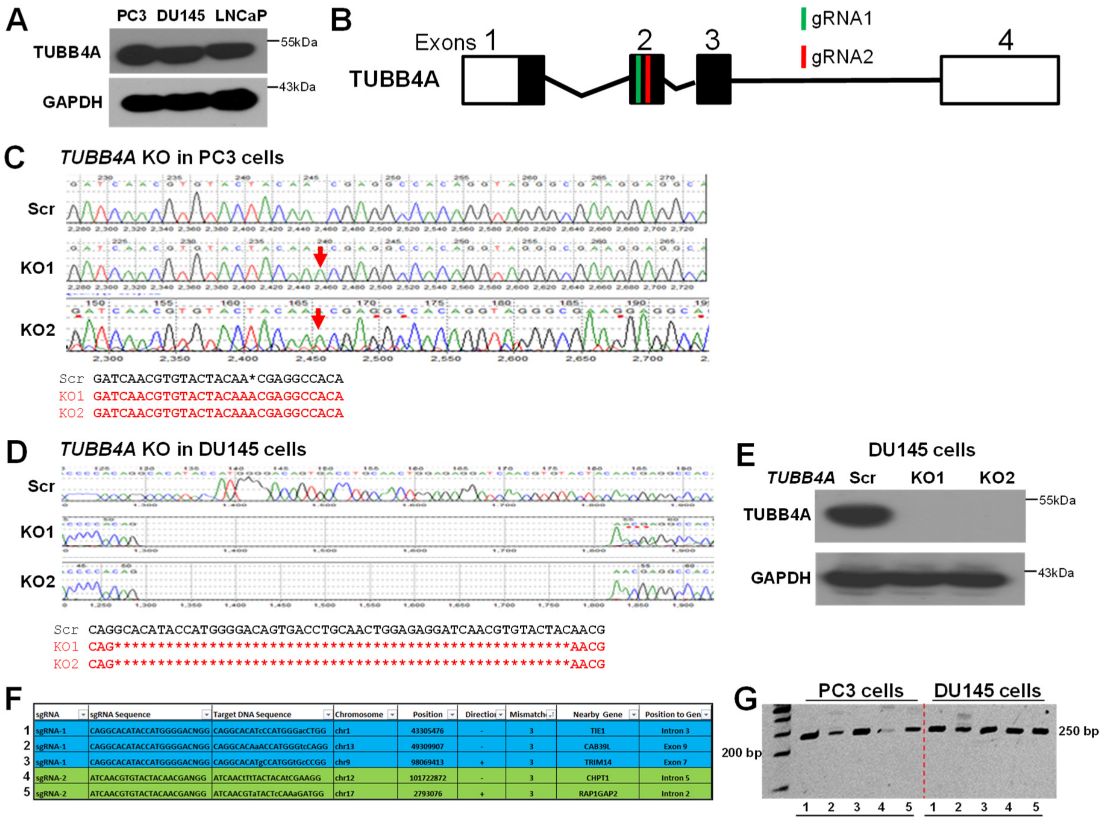


**Figure S2. CRISPR-Cas9 *TUBB4A* knockout (KO) in prostate cancer cell lines. A**, TUBB4A expression in three prostate cancer cell lines. **B**, Schematic diagram of guide RNA (gRNA) design at human *TUBB4A* gene. **C**, Sanger DNA sequencing of scrambled (Scr) and *TUBB4A* KO PC3 cells (red arrow indicates the base A insertion). **D, E**, Sanger DNA sequencing and Western blot confirmation of scrambled (Scr) and *TUBB4A* KO DU145 cells. **F**, The potential off-target regions as predicted by the Cas-OFFinder tool. **G**, PCR analysis of potential off-target genes in *TUBB4A* KO PC3 and DU145 cells. Source data are provided as a Source Data file.

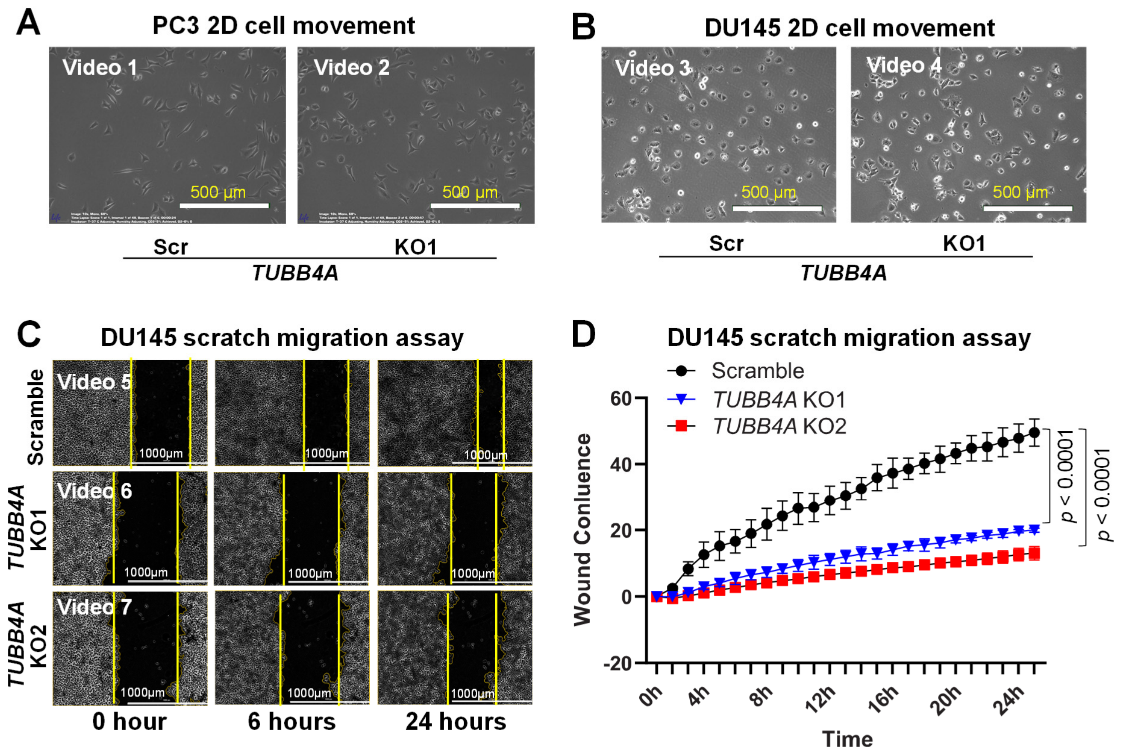


**Figure S3. Cell movement and migration of scrambled and *TUBB4A* KO prostate cancer cell lines. A**, Automated live-cell 2D video movement assay of PC3 cells (also see Supplementary Videos 1 and 2). Scale bar, 500 μm. **B**, Automated live-cell 2D video movement assay of DU145 cells (also see Supplementary Videos 3 and 4). Scale bar, 500 μm. **C, D**, Automated live-cell 2D video scratch migration assay and wound healing curves of DU145 cells (also see Supplementary Videos 5-7). Data are replicated 3 times (D). Data are presented as the means and SD with a repeated-measures ANOVA test. Source data are provided as a Source Data file. Scr, scramble; KO, knockout.

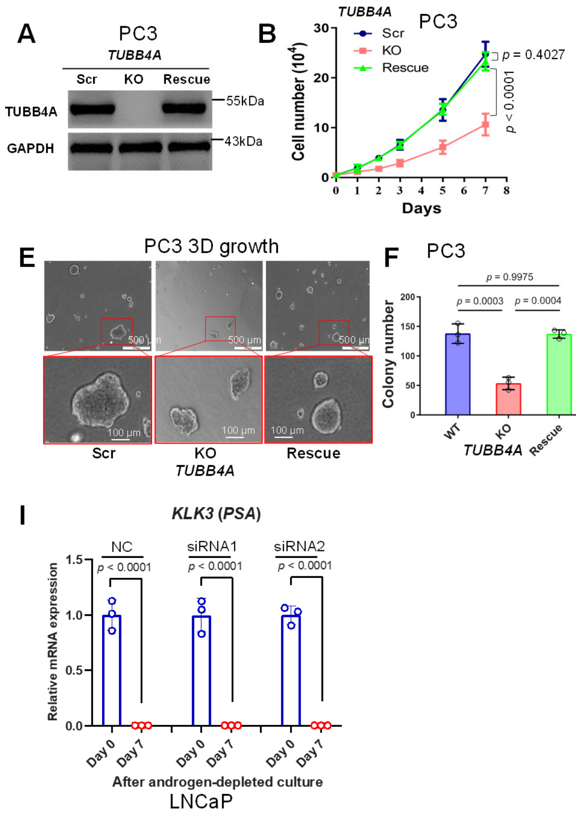


**Figure S4. Ectopic expression of TUBB4A rescues the *TUBB4A* KO-reduced proliferation and migration of prostate cancer cells. A-D,** Confirmation of *TUBB4A* KO and rescue, cell growth, clone formation, and statistical analysis of PC3 cells. A colony was considered to be 50 cells or more under a microscope. **E**, **F**, 3D soft-agar colony formation and statistical analysis of PC3 cells. A 3D colony was counted based on the capacity of single cells to grow to colonies consisting of at least 50 cells. Scale bar, 500 μm. **G**, **H**, Transwell assay and statistical analysis of PC3 cells. Scale bar, 50 μm. **I,** Relative mRNA levels of *KLK3 (PAS)* as a percentage of *GAPDH* expression as determined by qPCR in LNCaP cells transfected with negative control siRNA or *TUBB4A* siRNAs in androgen-depleted culture medium (charcoal-stripped growth medium). Data are replicated 3 (B, F, I) and 6 (D, H) times. Data are presented as the means and SD with a two-tailed *t* test (I), a repeated measures ANOVA test (B), or an ANOVA followed by Tukey's post hoc *t* test (D, F, H). Source data are provided as a Source Data file. Scr, scramble; KO, knockout, NC, negative control siRNA; siRNA, small interfering RNA.

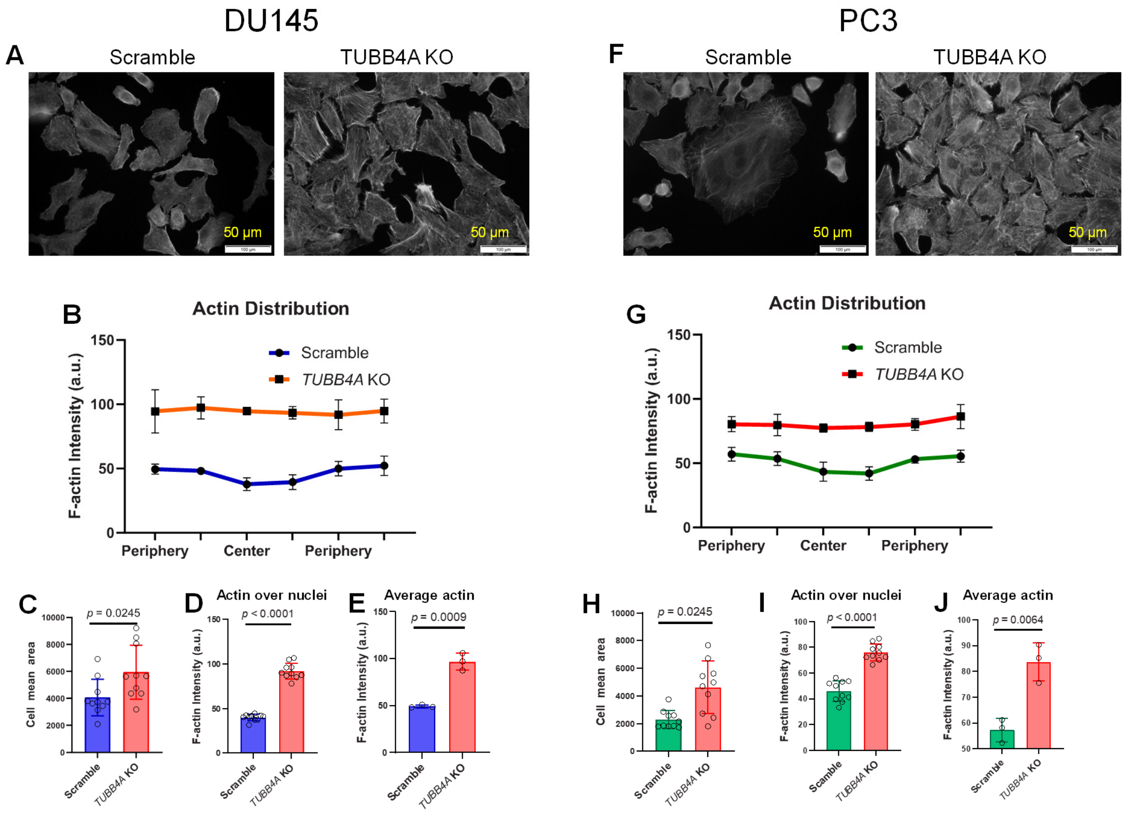


**Figure S5. *TUBB4A* KO increases the cell area and the F-actin intensity in prostate cancer cells. A,** Expression levels and localization of F-actin by immunofluorescence imaging in scrambled control and KO DU145 cells. **B,** Quantification of the distribution of F-actin intensity in scrambled control and KO DU145 cells. **C-E,** Quantification of the cell area, F-actin intensity over nuclei, and average F-actin intensity in scrambled control and KO DU145 cells. **F-J,** Expression levels, localization, distribution, and intensity of F-actin in scrambled control and KO PC3 cells. Data are replicated 3 (E, J) and 10 (C-D and H-I) times. Data are presented as the means and SD with a two-tailed *t* test (C-E and H-J). Source data are provided as a Source Data file. Scr, scrambled control; KO, knockout.

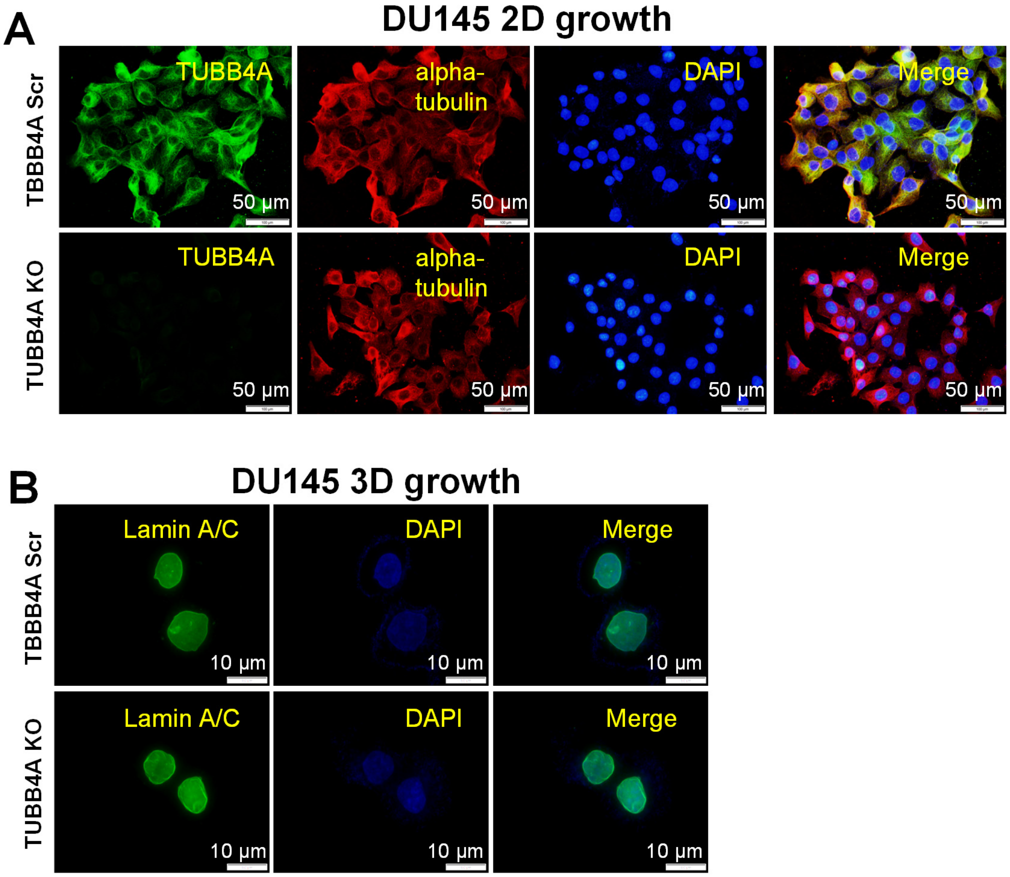


**Figure S6. *TUBB4A* KO does not change α-tubulin or lamin. A**, IF staining of TUBB4A and α-tubulin in DU145 cells in 2D collagen gels. **B**, IF staining of TUBB4A and lamin A/C in DU145 cells after migration in 3D collagen gels.

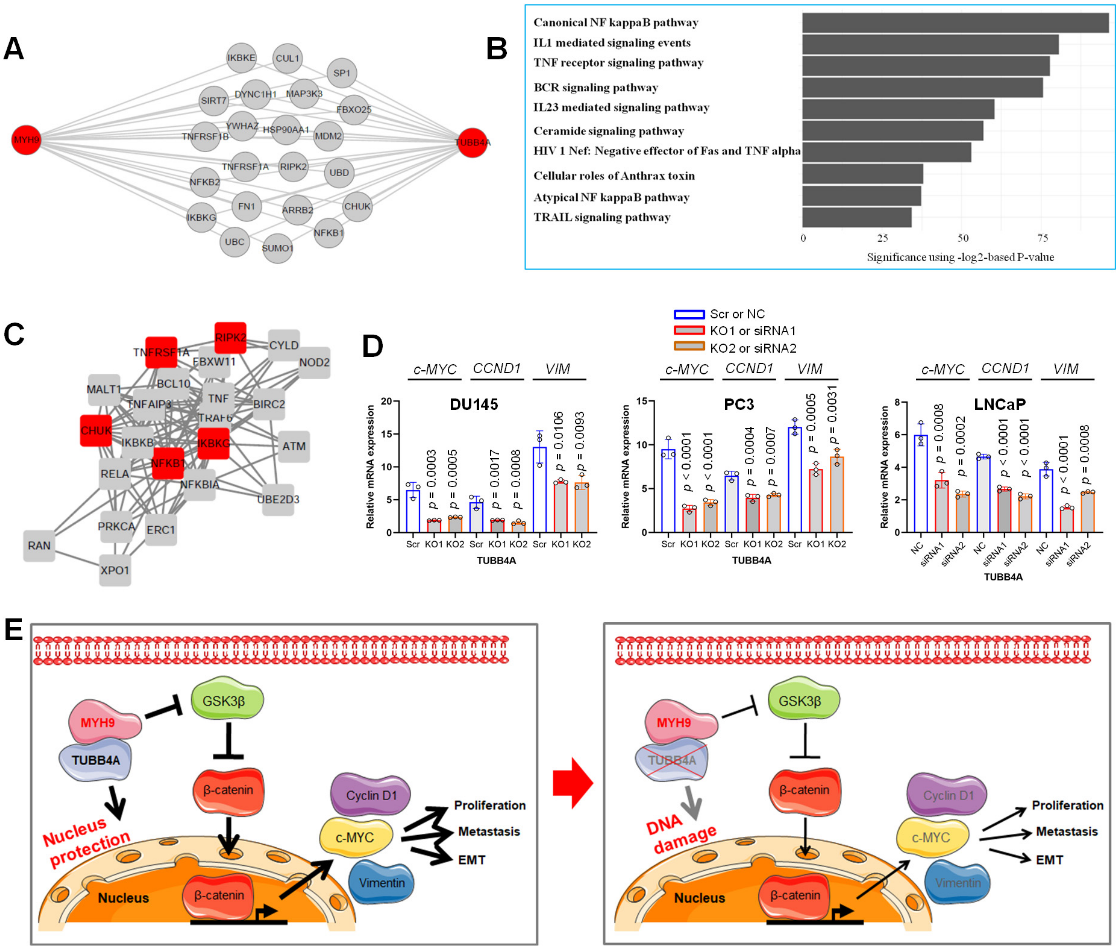

**Figure S7. TUBB4A-related signaling pathways and gene network. A,** Gene network as shared interactors between TUBB4A and MYH9. **B,** Top 10 TUBB4A/MYH9-related significantly enriched NCI-pathways ordered by the *p*-values using bioinformatics analysis with PAGER2 (Yue Z, *et al.* Nucleic Acids Res, 46, D668-D676, 2018). **C**, TUBB4A/MYH9-related 23 genes and 113 interactions regulated the gene network in the top canonical NF-κB signaling pathway. **D**, Expressions of *c-MYC, CCND1, and VIM* in scrambled control and *TUBB4A* KO DU145 and PC3 cells as well as NC control and *TUBB4A* siRNA-treated LNCaP cells. Data are replicated 3 times. Data are presented as the means and SD with an ANOVA followed by Tukey's post hoc *t* test (*TUBB4A* KO *vs*. scramble control or *TUBB4A* siRNA *vs*. NC control). Source data are provided as a Source Data file. **E,** Schematic diagram of TUBB4A/MYH9-mediated nucleus protection and -regulated GSK3β/β-catenin signaling pathway for cell proliferation, metastasis, and epithelial-mesenchymal transition (EMT) in prostate cancer cells. Scr, scramble; KO, knockout; NC, negative control siRNA; siRNA, small interfering RNA.

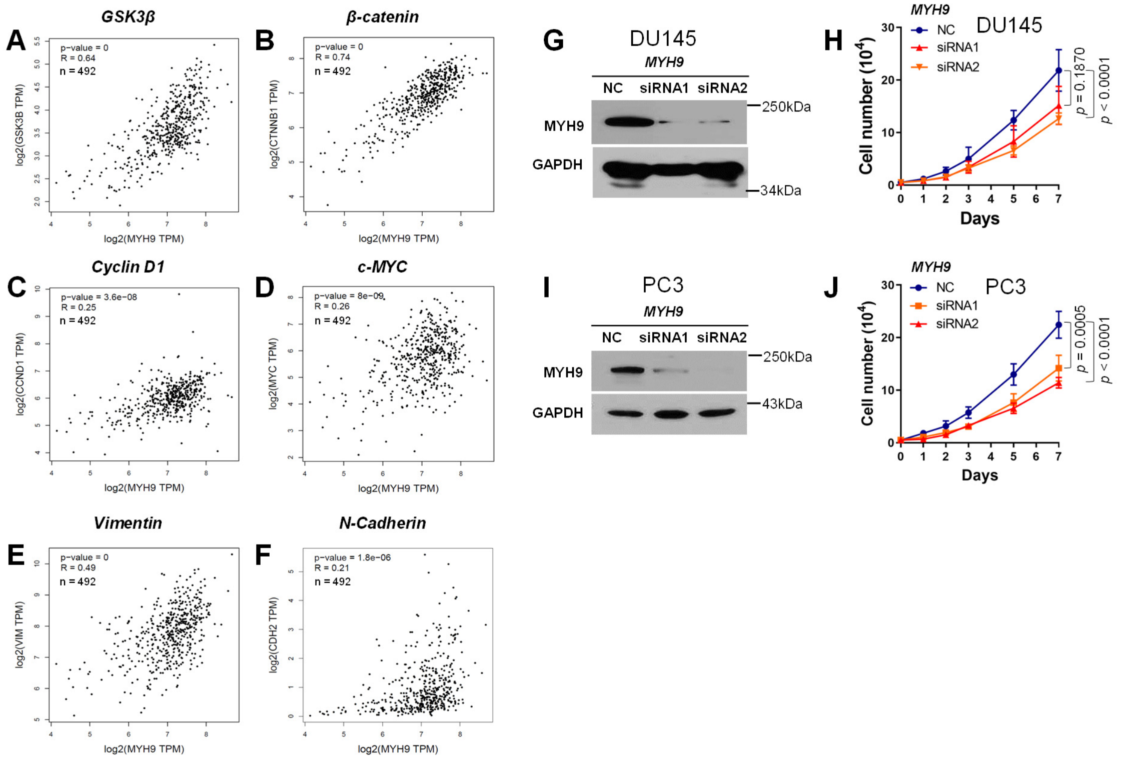


**Figure S8. MYH9-related GSK3β/β-catenin signaling gene network and the effect of *MYH9* knockdown on growth of prostate cancer cells. A-F,** Relationship between mRNA expression levels of *MYH9* with *GSK3β, β-catenin, cyclin D1, c-MYC, vimentin,* and *N-cadherin* in human primary prostate cancer tissues from TCGA dataset was analyzed using Gene Expression Profiling Interactive Analysis (GEPIA, gepia.cancer-pku.cn). **G-J,** Confirmation of *MYH9* knockdown by Western blots and effect of *MYH9* knockdown on proliferation of DU145 and PC3 cells. Data are replicated 3 times. Data are presented as the means and SD with a repeated measures ANOVA test. Source data are provided as a Source Data file. NC, negative control siRNA; siRNA, small interfering RNA.

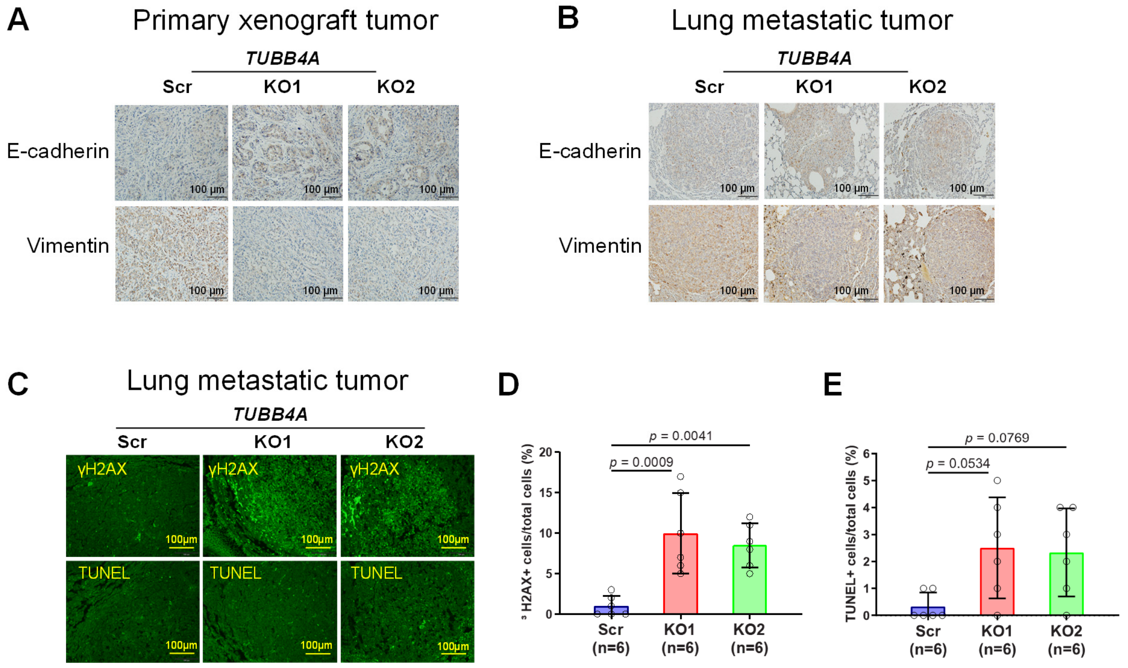


**Figure S9. Expression of EMT, DNA damage response and apoptotic markers in scrambled and *TUBB4A* KO DU145 xenograft tumors of NSG mice. A, B,** IHC staining of primary and metastatic tumors by specific antibodies to E-cadherin and vimentin for scrambled and KO groups. Scale bar, 100 µm. **C-E,** IF staining and statistical analysis of lung metastases by DNA damage response maker γH2AX and apoptotic marker TUNEL for scrambled and KO groups. Scale bar, 100 µm. Data are replicated with 6 times. Data are presented as the means and SD with an ANOVA followed by Tukey's post hoc *t* test (*TUBB4A* KO *vs*. scramble control). Source data are provided as a Source Data file. Scr, scramble; KO, knockout.

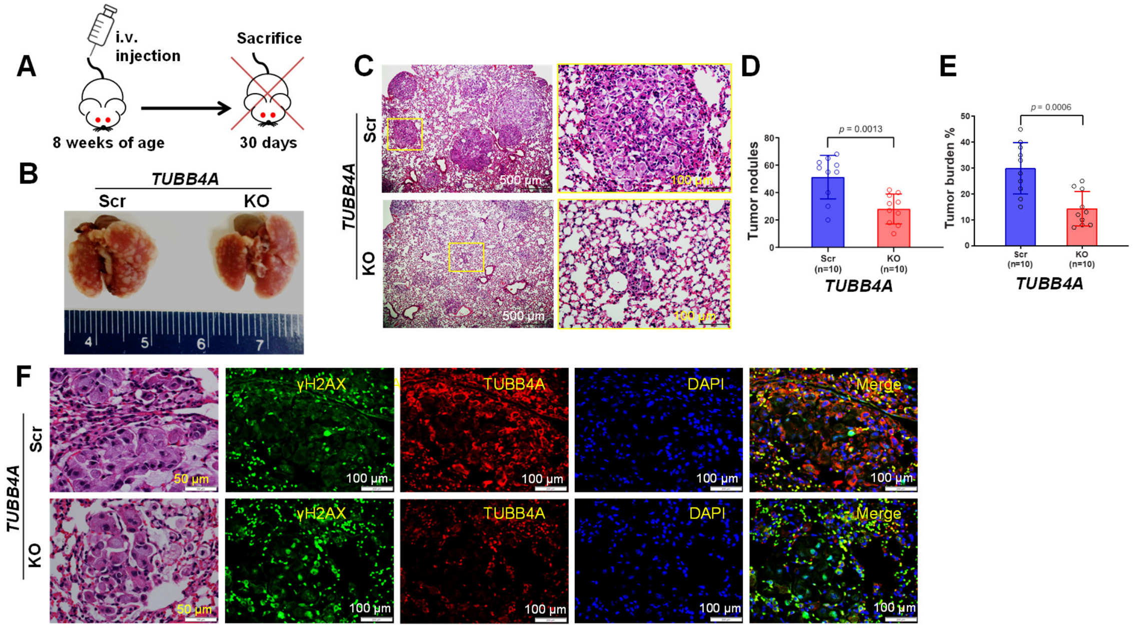


**Figure S10. *TUBB4A* KO reduces tumor colonization in the lungs of NSG mice.**

**A**, Schematic diagram of IV xenograft tumors followed for up to 30 days in NSG mice. **B, C,** Representative tumor metastases in the lung. **D, E,** Statistical analysis of tumor nodules and burdens of lung metastasis at day 30. Data are replicated 10 times. Data are presented as the means and SD with a two-tailed *t* test. Source data are provided as a Source Data file. **F,** H/E and IF staining of γH2A and TUBB4A in lung metastatic tumors.

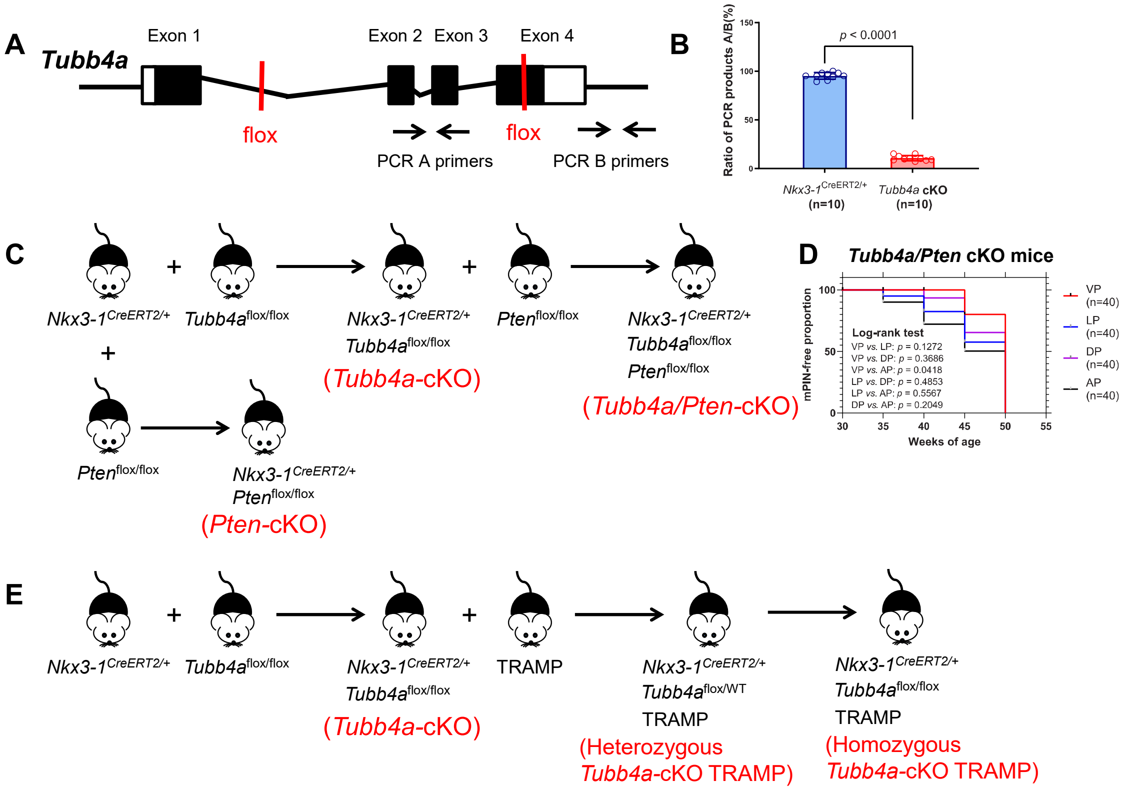


**Figure S11. Establishment of genetically engineered spontaneous prostate cancer mouse models. A,** Diagram of the floxed *Tubb4a* locus and position of the primers used to measure the ratio of deleted *versus* undeleted alleles. **B,** Efficient DNA deletion of the *Tubb4a* locus in the *Tubb4a* cKO but not the *Nkx3-1*$^{CreERT2/+}$ control mice. Data are replicated 10 times. Data are presented as the means and SD with a two-tailed *t* test. **C,** Diagram of the development of *Nkx3-1*$^{CreERT2/+}$*Tubb4a*$^{flox/flox}$ (*Tubb4a* cKO), *Nkx3-1*$^{CreERT2/+}$*Pten*$^{flox/flox}$ (*Pten* cKO), and *Nkx3-1*$^{CreERT2/+}$*Tubb4a*$^{flox/flox}$ *Pten*$^{flox/flox}$ (*Tubb4a/Pten* cKO) models. **D,** Kaplan–Meier curves of mPIN incidences for up to 50 weeks of age in AP, DP, LP, and VP lobes of *Tubb4a/Pten* cKO mice, respectively. Data are replicated 40 times. **E,** Diagram of the development of *Nkx3-1*$^{CreERT2/+}$*Tubb4a*$^{flox/flox}$ (*Tubb4a* cKO) TRAMP models. AP, anterior prostate; DP, dorsal prostate; LP, lateral prostate; VP, ventral prostate; cKO, prostate conditional knockout; mPIN, mouse prostatic intraepithelial neoplasia. Source data are provided as a Source Data file.

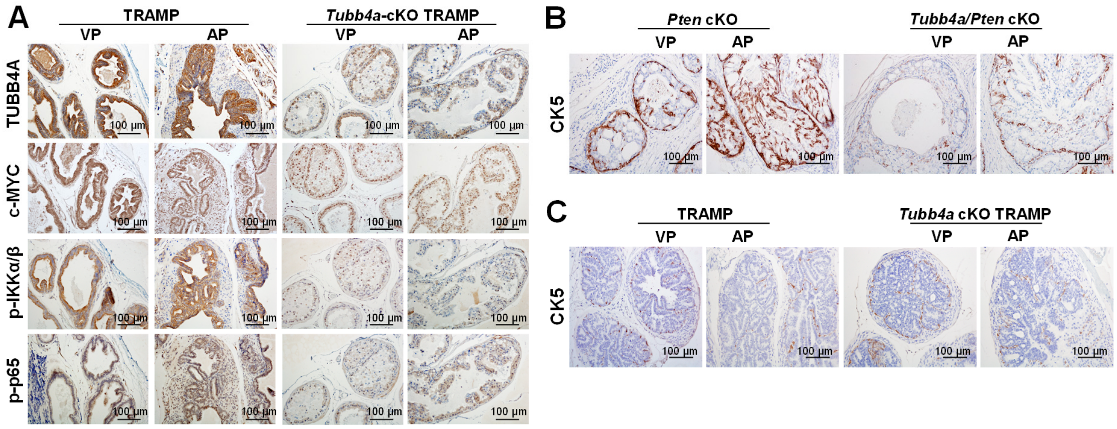


**Figure S12. Representative immunostaining of genetically engineered spontaneous prostate cancer mouse models. A,** Immunostaining for TUBB4A, AR, Ki67, c-MYC, p-IKK, p-p65 and in the prostates of wild-type TRAMP and *Tubb4a* cKO TRAMP mice at 12 weeks of age. **B, C,** Immunostaining for CK5 in the prostates of *Pten* cKO and *Tubb4a/Pten* cKO mice at 35 weeks of age and wild-type TRAMP and *Tubb4a* cKO TRAMP mice at 30 weeks of age. Scale bar, 100 µm.

**Supplementary Video 1. Automated live-cell two-dimensional random movement assay for scramble control PC3 cells.** Images were taken several times throughout 24 hours in a 6-well cell culture plate at 37°C under 5% $CO_2$. This video is related to Fig. S3A, left panel.

**Supplementary Video 2. Automated live-cell two-dimensional random movement assay for *TUBB4A* KO PC3 cells.** Images were taken several times throughout 24 hours in a 6-well cell culture plate at 37°C under 5% $CO_2$. This video is related to Fig. S3A, right panel.

**Supplementary Video 3. Automated live-cell two-dimensional random movement assay for scramble control DU145 cells.** Images were taken several times throughout 24 hours in a 6-well cell culture plate at 37°C under 5% $CO_2$. This video is related to Fig. S3B, left panel.

**Supplementary Video 4. Automated live-cell two-dimensional random movement assay for *TUBB4A* KO DU145 cells.** Images were taken several times throughout 24 hours in a 6-well cell culture plate at 37°C under 5% $CO_2$. This video is related to Fig. S3B, right panel.

**Supplementary Video 5. Automated live-cell two-dimensional scratch migration assay in scramble control DU145 cells.** Images were taken several times throughout 24 hours in a 6-well cell culture plate at 37°C under 5% $CO_2$. This video is related to Fig. S3C, left panel.

**Supplementary Video 6. Automated live-cell two-dimensional scratch migration assay in *TUBB4A* KO1 DU145 cells.** Images were taken several times throughout 24 hours in a 6-well cell culture plate at 37°C under 5% $CO_2$. This video is related to Fig. S3C, right panel.

**Supplementary Video 7. Automated live-cell two-dimensional scratch migration assay for *TUBB4A* KO2 DU145 cells.** Images were taken several times throughout 24 hours in a 6-well cell culture plate at 37°C under 5% $CO_2$. This video is related to Fig. S3C, right panel.

**Table S1. Human specimen characteristics**

| **Groups** | **Normal prostate** [a] n=50 | **Prostate cancer** n=136 |
|---|---|---|
| Age y, median (range) | 64 (47–76) | 63 (46–76) |
| PSA (ng/ml), median (range) | | 5.9 (1.9–46.8) |
| Race | | |
| European-Americans (EA) | 27 | 75 |
| African-Americans (AA) | 23 | 61 |
| Tumor stage (TNM) | | |
| T1 | | 0 |
| T2 | | 62 |
| T3 | | 51 |
| T4 or N+ or M+ | | 23 |
| Gleason score | | |
| G6 | | 14 |
| G7 | | 92 |
| G8 | | 10 |
| G9 | | 16 |
| G10 | | 4 |

[a] Histologically normal prostate tissue adjacent to the prostate cancer tissue

## Table S2. Mass spectrometry analysis for TUBB4A-interacting proteins

| Ranking | %Cov(95) [a] | Accession | Name | Species | Peptides(95%) |
|---|---|---|---|---|---|
| 1 | 41.12 | sp\|P35579\|MYH9_HUMAN | Myosin-9 OS=Homo sapiens GN=MYH9 PE=1 SV=4 | HUMAN | 77 |
| 2 | 34.75 | sp\|Q00610\|CLH1_HUMAN | Clathrin heavy chain 1 OS=Homo sapiens GN=CLTC PE=1 SV=5 | HUMAN | 59 |
| 3 | 22.63 | sp\|P46940\|IQGA1_HUMAN | Ras GTPase-activating-like protein IQGAP1 OS=Homo sapiens GN=IQGAP1 PE=1 SV=1 | HUMAN | 36 |
| 4 | 46.07 | sp\|P68371\|TBB4B_HUMAN | Tubulin beta-4B chain OS=Homo sapiens GN=TUBB4B PE=1 SV=1 | HUMAN | 21 |
| 5 | 9.31 | sp\|P35580\|MYH10_HUMAN | Myosin-10 OS=Homo sapiens GN=MYH10 PE=1 SV=3 | HUMAN | 20 |
| 6 | 38.96 | sp\|P07437\|TBB5_HUMAN | Tubulin beta chain OS=Homo sapiens GN=TUBB PE=1 SV=2 | HUMAN | 19 |
| 7 | 12.89 | sp\|O00159\|MYO1C_HUMAN | Unconventional myosin-Ic OS=Homo sapiens GN=MYO1C PE=1 SV=4 | HUMAN | 14 |
| 8 | 5.86 | sp\|P21333\|FLNA_HUMAN | Filamin-A OS=Homo sapiens GN=FLNA PE=1 SV=4 | HUMAN | 12 |
| 9 | 19.29 | sp\|P68363\|TBA1B_HUMAN | Tubulin alpha-1B chain OS=Homo sapiens GN=TUBA1B PE=1 SV=1 | HUMAN | 8 |
| 10 | 19.47 | sp\|P63261\|ACTG_HUMAN | Actin, cytoplasmic 2 OS=Homo sapiens GN=ACTG1 PE=1 SV=1 | HUMAN | 7 |
| 11 | 19.47 | sp\|P60709\|ACTB_HUMAN | Actin, cytoplasmic 1 OS=Homo sapiens GN=ACTB PE=1 SV=1 | HUMAN | 7 |
| 12 | 16.48 | sp\|Q9BQE3\|TBA1C_HUMAN | Tubulin alpha-1C chain OS=Homo sapiens GN=TUBA1C PE=1 SV=1 | HUMAN | 7 |
| 13 | 16.41 | sp\|Q71U36\|TBA1A_HUMAN | Tubulin alpha-1A chain OS=Homo sapiens GN=TUBA1A PE=1 SV=1 | HUMAN | 7 |
| 14 | 11.68 | sp\|P14618\|KPYM_HUMAN | Pyruvate kinase PKM OS=Homo sapiens GN=PKM PE=1 SV=4 | HUMAN | 7 |
| 15 | 5.72 | sp\|Q9UM54\|MYO6_HUMAN | Unconventional myosin-VI OS=Homo sapiens GN=MYO6 PE=1 SV=4 | HUMAN | 7 |
| 16 | 10.96 | sp\|Q9Y608\|LRRF2_HUMAN | Leucine-rich repeat flightless-interacting protein 2 OS=Homo sapiens GN=LRRFIP2 PE=1 SV=1 | HUMAN | 6 |
| 17 | 16.23 | sp\|Q5VTE0\|EF1A3_HUMAN | Putative elongation factor 1-alpha-like 3 OS=Homo sapiens GN=EEF1A1P5 PE=5 SV=1 | HUMAN | 6 |
| 18 | 16.23 | sp\|P68104\|EF1A1_HUMAN | Elongation factor 1-alpha 1 OS=Homo sapiens GN=EEF1A1 PE=1 SV=1 | HUMAN | 6 |
| 19 | 8.73 | sp\|P10809\|CH60_HUMAN | 60 kDa heat shock protein, mitochondrial OS=Homo sapiens GN=HSPD1 PE=1 SV=2 | HUMAN | 5 |
| 20 | 11.80 | sp\|P07355\|ANXA2_HUMAN | Annexin A2 OS=Homo sapiens GN=ANXA2 PE=1 SV=2 | HUMAN | 4 |
| 21 | 11.80 | sp\|A6NMY6\|AXA2L_HUMAN | Putative annexin A2-like protein OS=Homo sapiens GN=ANXA2P2 PE=5 SV=2 | HUMAN | 4 |
| 22 | 6.22 | sp\|P08238\|HS90B_HUMAN | Heat shock protein HSP 90-beta OS=Homo sapiens GN=HSP90AB1 PE=1 SV=4 | HUMAN | 4 |
| 23 | 2.22 | sp\|P41252\|SYIC_HUMAN | Isoleucine--tRNA ligase, cytoplasmic OS=Homo sapiens GN=IARS PE=1 SV=2 | HUMAN | 3 |
| 24 | 3.82 | sp\|Q09666\|AHNK_HUMAN | Neuroblast differentiation-associated protein AHNAK OS=Homo sapiens GN=AHNAK PE=1 SV=2 | HUMAN | 3 |
| 25 | 14.57 | sp\|Q06830\|PRDX1_HUMAN | Peroxiredoxin-1 OS=Homo sapiens GN=PRDX1 PE=1 SV=1 | HUMAN | 3 |
| 26 | 1.86 | sp\|P01860\|IGHG3_HUMAN | Ig gamma-3 chain C region OS=Homo sapiens GN=IGHG3 PE=1 SV=2 | HUMAN | 3 |
| 27 | 2.12 | sp\|P01857\|IGHG1_HUMAN | Ig gamma-1 chain C region OS=Homo sapiens GN=IGHG1 PE=1 SV=1 | HUMAN | 3 |
| 28 | 2.15 | sp\|P01859\|IGHG2_HUMAN | Ig gamma-2 chain C region OS=Homo sapiens GN=IGHG2 PE=1 SV=2 | HUMAN | 3 |
| 29 | 2.14 | sp\|P01861\|IGHG4_HUMAN | Ig gamma-4 chain C region OS=Homo sapiens GN=IGHG4 PE=1 SV=1 | HUMAN | 3 |
| 30 | 3.86 | sp\|P14625\|ENPL_HUMAN | Endoplasmin OS=Homo sapiens GN=HSP90B1 PE=1 SV=1 | HUMAN | 3 |
| 31 | 15.05 | sp\|P05109\|S10A8_HUMAN | Protein S100-A8 OS=Homo sapiens GN=S100A8 PE=1 SV=1 | HUMAN | 3 |
| 32 | 24.56 | sp\|P06702\|S10A9_HUMAN | Protein S100-A9 OS=Homo sapiens GN=S100A9 PE=1 SV=1 | HUMAN | 2 |
| 33 | 19.53 | sp\|P62987\|RL40_HUMAN | Ubiquitin-60S ribosomal protein L40 OS=Homo sapiens GN=UBA52 PE=1 SV=2 | HUMAN | 2 |
| 34 | 16.03 | sp\|P62979\|RS27A_HUMAN | Ubiquitin-40S ribosomal protein S27a OS=Homo sapiens GN=RPS27A PE=1 SV=2 | HUMAN | 2 |
| 35 | 32.85 | sp\|P0CG48\|UBC_HUMAN | Polyubiquitin-C OS=Homo sapiens GN=UBC PE=1 SV=3 | HUMAN | 2 |

| | | | | | |
|---|---|---|---|---|---|
| 36 | 32.75 | sp|P0CG47|UBB_HUMAN | Polyubiquitin-B OS=Homo sapiens GN=UBB PE=1 SV=1 | HUMAN | 2 |
| 37 | 1.66 | sp|Q9UPQ0|LIMC1_HUMAN | LIM and calponin homology domains-containing protein 1 OS=Homo sapiens GN=LIMCH1 PE=1 SV=4 | HUMAN | 2 |
| 38 | 1.40 | sp|P31327|CPSM_HUMAN | Carbamoyl-phosphate synthase [ammonia], mitochondrial OS=Homo sapiens GN=CPS1 PE=1 SV=2 | HUMAN | 2 |
| 39 | 1.09 | sp|Q14152|EIF3A_HUMAN | Eukaryotic translation initiation factor 3 subunit A OS=Homo sapiens GN=EIF3A PE=1 SV=1 | HUMAN | 2 |
| 40 | 13.91 | sp|P60660|MYL6_HUMAN | Myosin light polypeptide 6 OS=Homo sapiens GN=MYL6 PE=1 SV=2 | HUMAN | 2 |
| 41 | 4.08 | sp|P11413|G6PD_HUMAN | Glucose-6-phosphate 1-dehydrogenase OS=Homo sapiens GN=G6PD PE=1 SV=4 | HUMAN | 2 |
| 42 | 2.20 | sp|P00352|AL1A1_HUMAN | Retinal dehydrogenase 1 OS=Homo sapiens GN=ALDH1A1 PE=1 SV=2 | HUMAN | 1 |
| 43 | 1.91 | sp|P08195|4F2_HUMAN | 4F2 cell-surface antigen heavy chain OS=Homo sapiens GN=SLC3A2 PE=1 SV=3 | HUMAN | 1 |
| 44 | 0.60 | sp|P07814|SYEP_HUMAN | Bifunctional glutamate/proline--tRNA ligase OS=Homo sapiens GN=EPRS PE=1 SV=5 | HUMAN | 1 |
| 45 | 0.83 | sp|P00533|EGFR_HUMAN | Epidermal growth factor receptor OS=Homo sapiens GN=EGFR PE=1 SV=2 | HUMAN | 1 |
| 46 | 0.57 | sp|P42704|LPPRC_HUMAN | Leucine-rich PPR motif-containing protein, mitochondrial OS=Homo sapiens GN=LRPPRC PE=1 SV=3 | HUMAN | 1 |
| 47 | 1.05 | sp|Q9Y5B9|SP16H_HUMAN | FACT complex subunit SPT16 OS=Homo sapiens GN=SUPT16H PE=1 SV=1 | HUMAN | 1 |
| 48 | 0.93 | sp|P17301|ITA2_HUMAN | Integrin alpha-2 OS=Homo sapiens GN=ITGA2 PE=1 SV=1 | HUMAN | 1 |
| 49 | 3.74 | sp|B9A064|IGLL5_HUMAN | Immunoglobulin lambda-like polypeptide 5 OS=Homo sapiens GN=IGLL5 PE=2 SV=2 | HUMAN | 1 |
| 50 | 8.11 | sp|P61626|LYSC_HUMAN | Lysozyme C OS=Homo sapiens GN=LYZ PE=1 SV=1 | HUMAN | 1 |
| 51 | 1.07 | sp|P63010|AP2B1_HUMAN | AP-2 complex subunit beta OS=Homo sapiens GN=AP2B1 PE=1 SV=1 | HUMAN | 1 |
| 52 | 1.99 | sp|P39023|RL3_HUMAN | 60S ribosomal protein L3 OS=Homo sapiens GN=RPL3 PE=1 SV=2 | HUMAN | 1 |
| 53 | 3.53 | sp|A6NCN2|KR87P_HUMAN | Putative keratin-87 protein OS=Homo sapiens GN=KRT87P PE=5 SV=4 | HUMAN | 1 |
| 54 | 0.45 | sp|Q8TF72|SHRM3_HUMAN | Protein Shroom3 OS=Homo sapiens GN=SHROOM3 PE=1 SV=2 | HUMAN | 1 |
| 55 | 1.76 | sp|Q14677|EPN4_HUMAN | Clathrin interactor 1 OS=Homo sapiens GN=CLINT1 PE=1 SV=1 | HUMAN | 1 |
| 56 | 2.46 | sp|P02768|ALBU_HUMAN | Serum albumin OS=Homo sapiens GN=ALB PE=1 SV=2 | HUMAN | 1 |
| 57 | 2.58 | sp|P31997|CEAM8_HUMAN | Carcinoembryonic antigen-related cell adhesion molecule 8 OS=Homo sapiens GN=CEACAM8 PE=1 SV=2 | HUMAN | 1 |
| 58 | 1.71 | sp|P13688|CEAM1_HUMAN | Carcinoembryonic antigen-related cell adhesion molecule 1 OS=Homo sapiens GN=CEACAM1 PE=1 SV=2 | HUMAN | 1 |
| 59 | 1.28 | sp|P06731|CEAM5_HUMAN | Carcinoembryonic antigen-related cell adhesion molecule 5 OS=Homo sapiens GN=CEACAM5 PE=1 SV=3 | HUMAN | 1 |
| 60 | 3.24 | sp|P61981|1433G_HUMAN | 14-3-3 protein gamma OS=Homo sapiens GN=YWHAG PE=1 SV=2 | HUMAN | 1 |
| 61 | 3.25 | sp|Q04917|1433F_HUMAN | 14-3-3 protein eta OS=Homo sapiens GN=YWHAH PE=1 SV=4 | HUMAN | 1 |
| 62 | 3.27 | sp|P63104|1433Z_HUMAN | 14-3-3 protein zeta/delta OS=Homo sapiens GN=YWHAZ PE=1 SV=1 | HUMAN | 1 |
| 63 | 3.14 | sp|P62258|1433E_HUMAN | 14-3-3 protein epsilon OS=Homo sapiens GN=YWHAE PE=1 SV=1 | HUMAN | 1 |
| 64 | 3.23 | sp|P31947|1433S_HUMAN | 14-3-3 protein sigma OS=Homo sapiens GN=SFN PE=1 SV=1 | HUMAN | 1 |
| 65 | 3.25 | sp|P31946|1433B_HUMAN | 14-3-3 protein beta/alpha OS=Homo sapiens GN=YWHAB PE=1 SV=3 | HUMAN | 1 |
| 66 | 3.27 | sp|P27348|1433T_HUMAN | 14-3-3 protein theta OS=Homo sapiens GN=YWHAQ PE=1 SV=1 | HUMAN | 1 |
| 67 | 2.48 | sp|P42330|AK1C3_HUMAN | Aldo-keto reductase family 1 member C3 OS=Homo sapiens GN=AKR1C3 PE=1 SV=4 | HUMAN | 1 |
| 68 | 2.48 | sp|Q04828|AK1C1_HUMAN | Aldo-keto reductase family 1 member C1 OS=Homo sapiens GN=AKR1C1 PE=1 SV=1 | HUMAN | 1 |
| 69 | 2.48 | sp|P52895|AK1C2_HUMAN | Aldo-keto reductase family 1 member C2 OS=Homo sapiens GN=AKR1C2 PE=1 SV=3 | HUMAN | 1 |
| 70 | 2.48 | sp|P17516|AK1C4_HUMAN | Aldo-keto reductase family 1 member C4 OS=Homo sapiens GN=AKR1C4 PE=1 SV=3 | HUMAN | 1 |
| 71 | 0.63 | sp|P54289|CA2D1_HUMAN | Voltage-dependent calcium channel subunit alpha-2/delta-1 OS=Homo sapiens GN=CACNA2D1 PE=1 SV=3 | HUMAN | 1 |

[a] %Cov (95): percentage of cover 95% peptides

**Table S3 Specific primary antibodies used in this study**

| | Antigen | Supplier | Cat # | Clone | Dilution |
|---|---|---|---|---|---|
| **Western blotting** | TUBB4A | Abcam | ab179509 | EPR16776 | 3000 |
| | MYH9 | Proteintech Rosemont | 14844-1-AP | | 1000 |
| | E-cadherin | Abcam | ab76055 | M168 | 3000 |
| | N-cadherin | Abcam, | ab18203 | | 2000 |
| | Vimentin | Abcam | ab92547 | EPR3776 | 2000 |
| | c-MYC | Abcam, | ab32072 | Y69 | 3000 |
| | CyclinD1 | Abcam | ab134175 | EPR2241 | 2000 |
| | p44/42 MAPK (Erk1/2) | Cell Signaling Technology | 4695 | 137F5 | 3000 |
| | p-p44/42 MAPK (Erk1/2) (Thr202/Tyr204) | Cell Signaling Technology | 4376 | 20G11 | 2000 |
| | p38 MAPK | Cell Signaling Technology | 9212 | | 3000 |
| | p-p38 MAPK (Thr180/Tyr182) | Cell Signaling Technology | 9211 | | 2000 |
| | TRAF6 | Cell Signaling Technology | 8028 | D21G3 | 1000 |
| | IRAK1 | Abcam | ab238 | | 2000 |
| | IKKα | Cell Signaling Technology | 2682 | | 2000 |
| | p-IKKα/β (Ser176/180) | Cell Signaling Technology | 2697 | 16A6 | 1000 |
| | IKKγ | Cell Signaling Technology | 2685 | | 2000 |
| | p-IKKγ (Ser376) | Cell Signaling Technology | 2689 | | 1000 |
| | NF-κB p65 | Santa Cruz Biotechnology | sc-514451 | A-12 | 2000 |
| | NF-κB p-p65 | Abcam | ab86299 | S536 | 1000 |
| | ATM | Santa Cruz Biotechnology | sc-377293 | G12 | 1000 |
| | p-ATM (Ser1981) | Abcam | ab81292 | EP1890Y | 1000 |
| | BCL-2 | Santa Cruz Biotechnology | sc-7382 | C-2 | 1000 |
| | BCL-XL | Cell Signaling Technology | 2764P | 54H6 | 1000 |
| | Caspase3 | Santa Cruz Biotechnology | sc-56053 | 31A1067 | 500 |
| | MEK1/2 | Proteintech Rosemont | 11049-1-AP | | 1000 |
| | p-MEK1/2 | Cell Signaling Technology | 9121 | Ser217/221 | 1000 |
| | TAK1 | Proteintech Rosemont | 12330-2-AP | | 1000 |
| | p-TAK1 | Proteintech Rosemont | 28958-1-AP | | 1000 |
| | GSK3β | Cell Signaling Technology | 9315 | 27C10 | 2000 |
| | p-GSK3β | Cell Signaling Technology | 5558S | S9 | 2000 |
| | β-catenin | BD Biotechnology | 610154 | | 2000 |
| | Lamin B | Santa Cruz Biotechnology | sc-374015 | B-10 | 250 |
| | GAPDH | Cell Signaling Technology | 5174 | D16H11 | 5000 |
| **IF** | TUBB4A | Abcam | ab11315 | ONS.1A6 | 200 |

| | | | | | |
|---|---|---|---|---|---|
| | MYH9 | Proteintech Rosemont | 60233-1-Ig | | 200 |
| | γH2AX | Cell Signaling Technology | 9718 | 20E3 Rb | 200 |
| | 53BP1 | Cell Signaling Technology | 4937 | | 200 |
| | alpha-tubulin | Sigma-Aldrich | T6199 | DM1A | 500 |
| | Lamin A/C | Santa Cruz Biotechnology | sc-376248 | E-1 | |
| | Goat-anti mouse IgG H&L | Abcam | ab150117 | Alex 488 | 500 |
| | Goat-anti mouse IgG H&L | Abcam | ab175473 | Alex 568 | 500 |
| | Alexa Fluor® 568 phalloidin (for F-actin) | Thermo Fisher Scientific | A12380 | | 500 |
| **IHC** | TUBB4A | Abcam | ab179509 | EPR16776 | 500 |
| | Androgen Receptor | Santa Cruz Biotechnology | sc-7305 | 441 | 200 |
| | Ki67 | Abcam | ab15580 | | 500 |
| | γH2AX | Cell Signaling Technology | 9718 | 20E3 Rb | 200 |
| | TUNEL | ABP Biosciences | A050 | | |
| | E-cadherin | Abcam | ab76055 | M168 | 500 |
| | Vimentin | Abcam | ab92547 | EPR3776 | 200 |
| | CK5 | Abcam | ab52635 | EP1601Y | 500 |
| | CyclinD1 | Abcam | ab134175 | EPR2241 | 200 |
| | c-MYC | Abcam | ab32072 | Y69 | 200 |
| | NF-κB p-p65 | Abcam | ab86299 | S536 | 100 |
| | NF-κB p-p66 | Sigma-Aldrich | SAB5700363 | | 100 |
| | p-IKKα/β (Ser176/180) | Cell Signaling Technology | 2697 | | 100 |

## Table S4 The sequences of primer, sgRNA, siRNA, and ORF used in this study

| Primer Name | Sequence |
|---|---|
| **Gene KO testing PCR primers** | |
| Mouse Tubb4a A primer-F | ATCAGTGATGAGCACGGCAT |
| Mouse Tubb4a A primer-R | TACAAAGTTGTCTGGCCGAA |
| Mouse Tubb4a B primer-F | AGTGGTGGGGTACAGGGATT |
| Mouse Tubb4a B primer-R | GAAGATGCATTCAGTGTGGC |
| **CRISP/Cas9 sgRNAs** | |
| Human TUBB4A gRNA1 | CACCGTCCCCATGGTATGTGCCTG |
| Human TUBB4A gRNA2 | AAACCAGGCACATACCATGGGGAC |
| **RT-PCR primers** | |
| Mouse c-Myc-F | CTAGTGCTGCATGAGGAGACA |
| Mouse c-Myc-R | TGTGCGGAGGTTTGCTGT |
| Mouse Ccnd1-F | TGCGTGCAGAAGGAGATTGT |
| Mouse Ccnd1-R | CTTCTTCAAGGGCTCCAGGG |
| Mouse Vim-F | CTCCAGAGAGAGGAAGCCGA |
| Mouse Vim-R | CGTTCAAGGTCAAGACGTGC |
| Mouse Hprt-realtime-F | CAGGCCAGACTTTGTTGGAT |
| Mouse Hprt-realtime-R | GCGCTCATCTTAGGCTTTGT |
| Human KLK3 (PSA)-F | CGTGACGTGGATTGGTGCT |
| Human KLK3 (PSA)-R | ACCCAGCAAGATCACGCTTT |
| Human GAPDH-F | CCCCTTCATTGACCTCAACTACAT |
| Human GAPDH-R | CGCTCCTGGAAGATGGTGA |
| **CRISP/Cas9 off-target PCR primers** | |
| TIE-off-target-F | CCATGGGACCCTCAGCTTTG |
| TIE-off-target-R | CCAGGTGGGCTATGTGACAG |
| CAB39L-off-target-F | CTAGAGAACTGTGAACATGTGGC |
| CAB39L-off-target-R | ACACATGAATCATTTCTCCTTCCA |
| TRIM14-off-target-F | CGTGACAGCCTCCAGAATGTCATG |
| TRIM14-off-target-R | GTGTGATCTCGGCTCACTGCAAC |
| CHPT1-off-target-F | ATAAGCTGGGTGTGGTGGCTCTC |
| CHPT1-off-target-R | CACACACGTTTTAAACTGGCTA |
| RAP1GAP2-off-target-F | GGCCGGAGTCTGTCCCGGCTCTG |
| RAP1GAP2-off-target-R | CACGCCACTGCACTCCAGCCTG |
| **Mouse genotyping primers** | |
| Tubb4a-21673 | ATGGGGTCCTAGGGAATGAG |
| Tubb4a-23806 | AGTTAGTGGATGCCGTCCTG |
| Tubb4a-23808 | TCTCATCAGTGTTCTCCACCA |
| Tubb4a-oIMR7202 | CGGTCGCTACCATTACCAGT |
| Pten-oIMR9554 | CAAGCACTCTGCGAACTGAG |
| Pten-oIMR9555 | AAGTTTTTGAAGGCAAGATGC |
| TRAMP-10363 | TAC AAC TGC CAA CTG GGA TG |
| TRAMP-10364 | CAG GCA CTC CTT TCA AGA CC |
| TRAMP-21238 | CTG TCC CTG TAT GCC TCT GG |
| TRAMP-21239 | AGA TGG AGA AAG GAC TAG GCT ACA |
| Nkx3-1CreWT-F | CTCCGCTACCCTAAGCATCC |
| Nkx3-1CreWT-R | GACACTGTCATATTACTTGGACC |
| Nkx3-1CreMut-F | CAGATGGCGCGGCAACACC |
| Nkx3-1CreMut-R | GCGCGGTCTGGCAGTAAAAAC |

| | |
|---|---|
| **siRNA sequences** | |
| Human TUBB4A siRNA-1-SEQ1 | GCAACAUGAAUGACCUGGUAUCUGA |
| Human TUBB4A siRNA-1-SEQ2 | UCAGAUACCAGGUCAUUCAUGUUGCUC |
| Human TUBB4A siRNA-2-SEQ1 | CAACAUGAAUGACCUGGUAUCUGAG |
| Human TUBB4A siRNA-2-SEQ2 | CUCAGAUACCAGGUCAUUCAUGUUGCU |
| Human MYH9 siRNA13.1-SEQ1 | AUGUUUUCCACUGAGCAUCACAATA |
| Human MYH9 siRNA13.1-SEQ2 | UAUUGUGAUGCUCAGUGGAAAACAUCU |
| Human MYH9 siRNA13.2-SEQ1 | GACCGAGAAGAUCAAUCCAUCUUGT |
| Human MYH9 siRNA13.2-SEQ2 | ACAAGAUGGAUUGAUCUUCUCGGUCUU |
| **TUBB4A open reading frame (ORF) sequence** | [illegible]<br>CGGCCAGTGCGGCAACCAGATCGGGGCCAAGTTTTGGG<br>AGGTTATCAGTGACGAACATGGCATCGACCCCACAGGCA<br>CATACCATGGGGACAGTGACCTGCAACTGGAGAGGATCA<br>ACGTGTACTACAACGAGGCCACAGGAGGAAATTATGTCC<br>CCAGAGCGGTGCTGGTGGACCTGGAACCCGGCACCATG<br>GACTCTGTCCGTTCTGGCCCCTTCGGTCAGATCTTTCGG<br>CCGGACAACTTCGTGTTTGGCCAATCCGGAGCCGGCAAC<br>AACTGGGCAAAGGGGCACTACACGGAGGGCGCAGAGCT<br>GGTGGACGCTGTCCTGGACGTAGTCCGGAAGGAGGCCG<br>AGAGCTGCGACTGCCTTCAGGGCTTCCAGCTGACCCACT<br>CGCTGGGGGGTGGCACGGGGTCCGGAATGGGCACGCT<br>GCTCATCAGTAAGATCCGCGAGGAGTTCCCAGACCGCAT<br>CATGAACACCTTCAGCGTGGTGCCCTCGCCCAAAGTGTC<br>AGACACGGTGGTGGAGCCCTACAACGCCACGCTGTCTGT<br>GCACCAGCTGGTGGAGAATACGGATGAGACCTACTGCAT<br>CGACAACGAGGCACTCTACGACATCTGTTTCCGCACCCT<br>CAAGCTGACCACCCCCACCTACGGGGACCTCAACCACCT<br>GGTGTCGGCCACCATGAGCGGGGTCACCACCTGCCTGC<br>GCTTCCCGGGCCAGCTGAACGCCGACCTGCGCAAGCTG<br>GCCGTCAACATGGTTCCCTTTCCTCGCCTGCACTTCTTCA<br>TGCCCGGCTTCGCACCCCTGACCAGCCGGGGCAGCCAG<br>CAGTACCGGGCCCTGACGGTGCCCGAGCTCACCCAGCA<br>GATGTTCGATGCCAAGAACATGATGGCGGCGTGCGACCC<br>GCGCCACGGCCGCTACCTGACCGTGGCCGCCGTGTTCC<br>GGGGCCGCATGTCCATGAAGGAGGTGGACGAGCAGATG<br>CTGAGCGTGCAGAGCAAGAACAGCAGCTACTTCGTGGAG<br>TGGATCCCCAACAACGTGAAGACGGCCGTGTGCGACATC<br>CCGCCCCGCGGCCTGAAGATGGCCGCGACCTTCATCGG<br>CAACAGCACGGCCATCCAGGAGCTGTTCAAGCGCATCTC<br>CGAGCAGTTCACGGCCATGTTCCGGCGCAAGGCCTTCTT<br>GCACTGGTACACGGGCGAGGGCATGGACGAGATGGAGT<br>TCACCGAGGCCGAGAGCAACATGAATGACCTGGTATCTG<br>AGTACCAGCAGTACCAGGACGCCACGGCCGAGGAGGGC<br>GAGTTCGAGGAGGAGGCGGAGGAGGAGGTGGCCTAGG<br>CTGCTCCCATCGCTTCCCACC |

# Fig. 2A, 2E, 2I

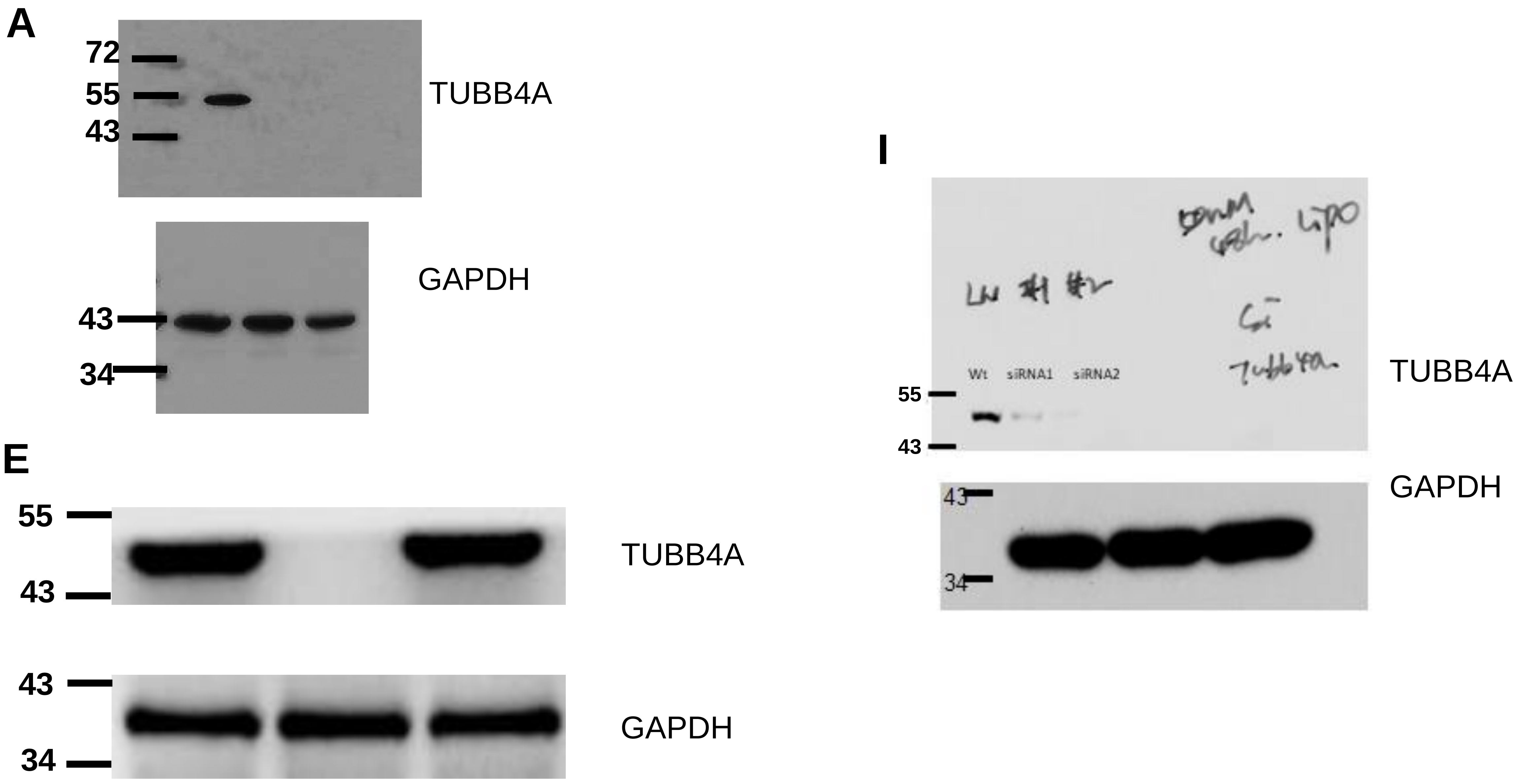
A
72
55
43
TUBB4A
GAPDH
43
34
E
55
43
TUBB4A
43
34
GAPDH
I
Wt
siRNA1
siRNA2
55
43
TUBB4A
GAPDH
43
34

**Fig. 4C**

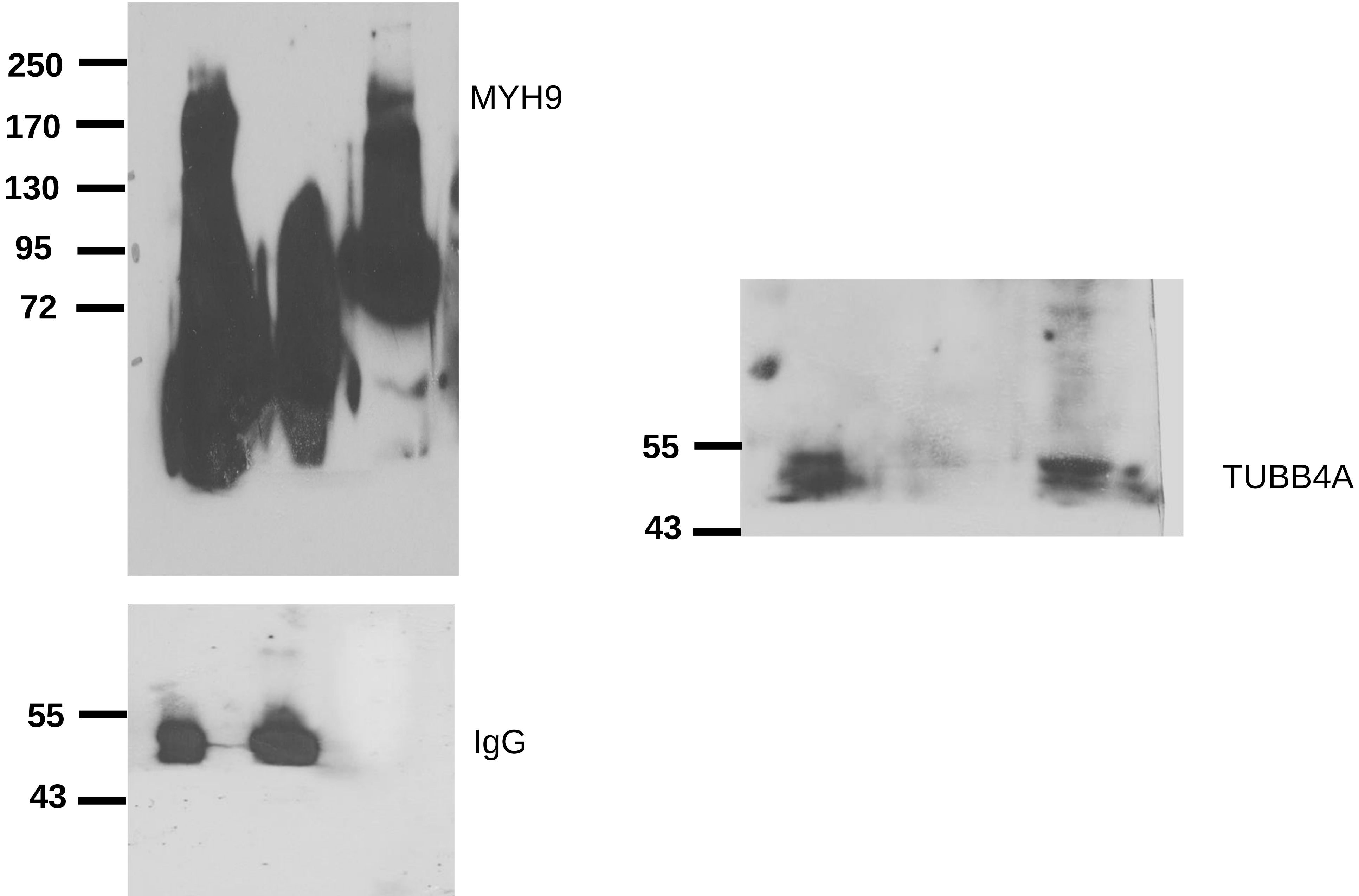

Fig. 4D

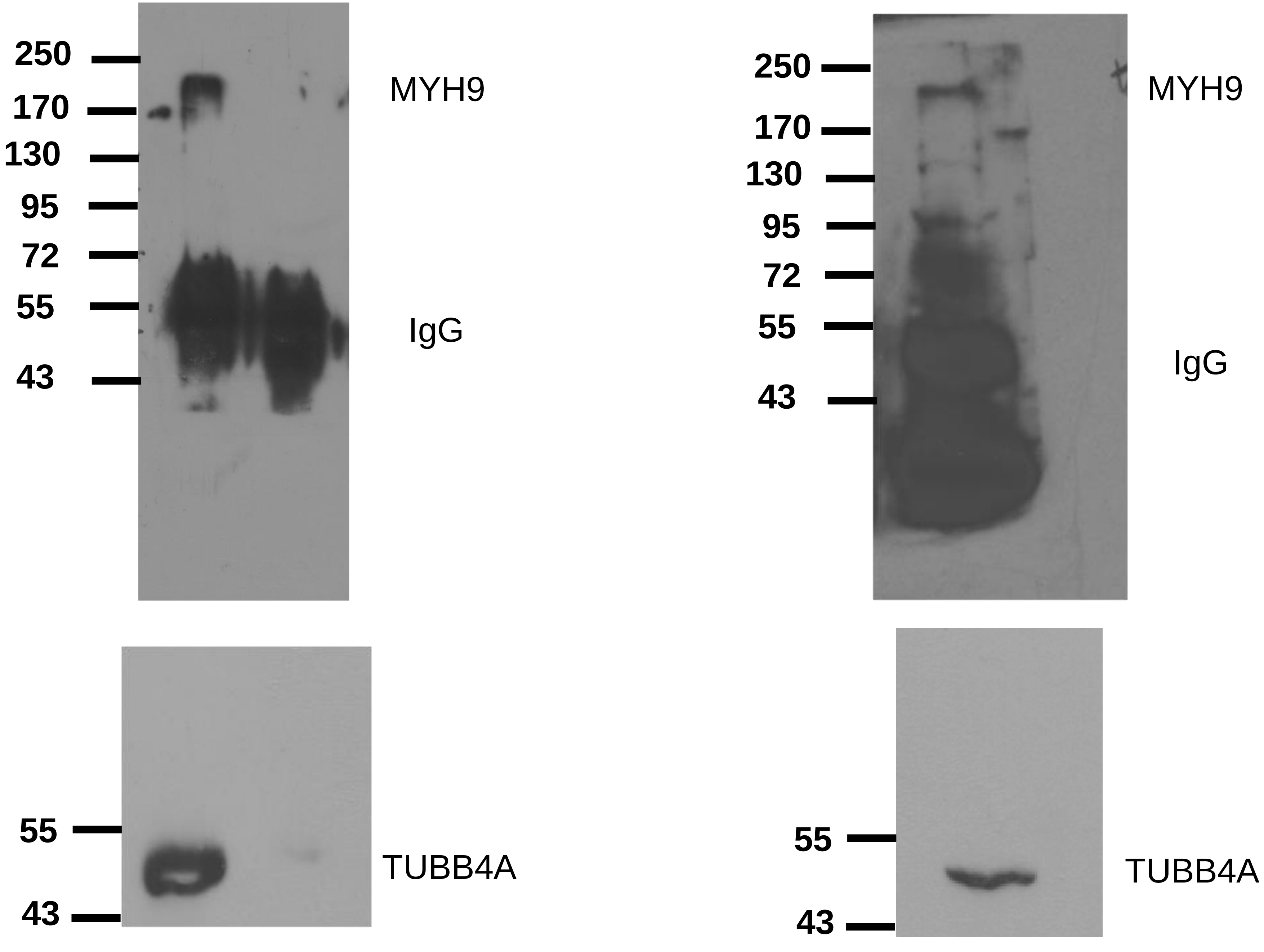
250
170
130
95
72
55
43
MYH9
IgG
250
170
130
95
72
55
43
MYH9
IgG
55
43
TUBB4A
55
43
TUBB4A

## Fig. 4G

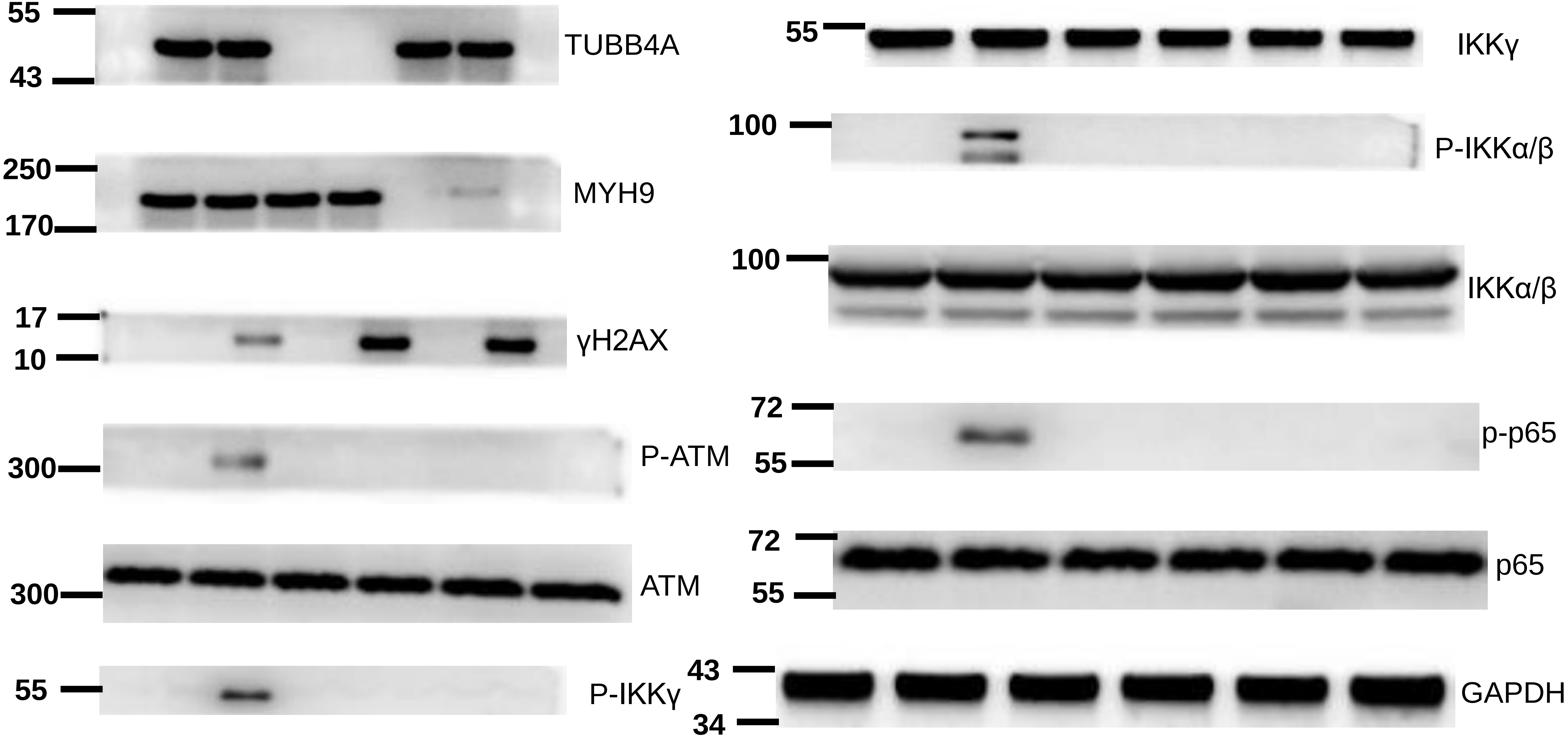
55
43
TUBB4A
250
170
MYH9
17
10
γH2AX
300
P-ATM
300
ATM
55
P-IKKγ
55
IKKγ
100
P-IKKα/β
100
IKKα/β
72
55
p-p65
72
55
p65
43
34
GAPDH

Fig. 4H

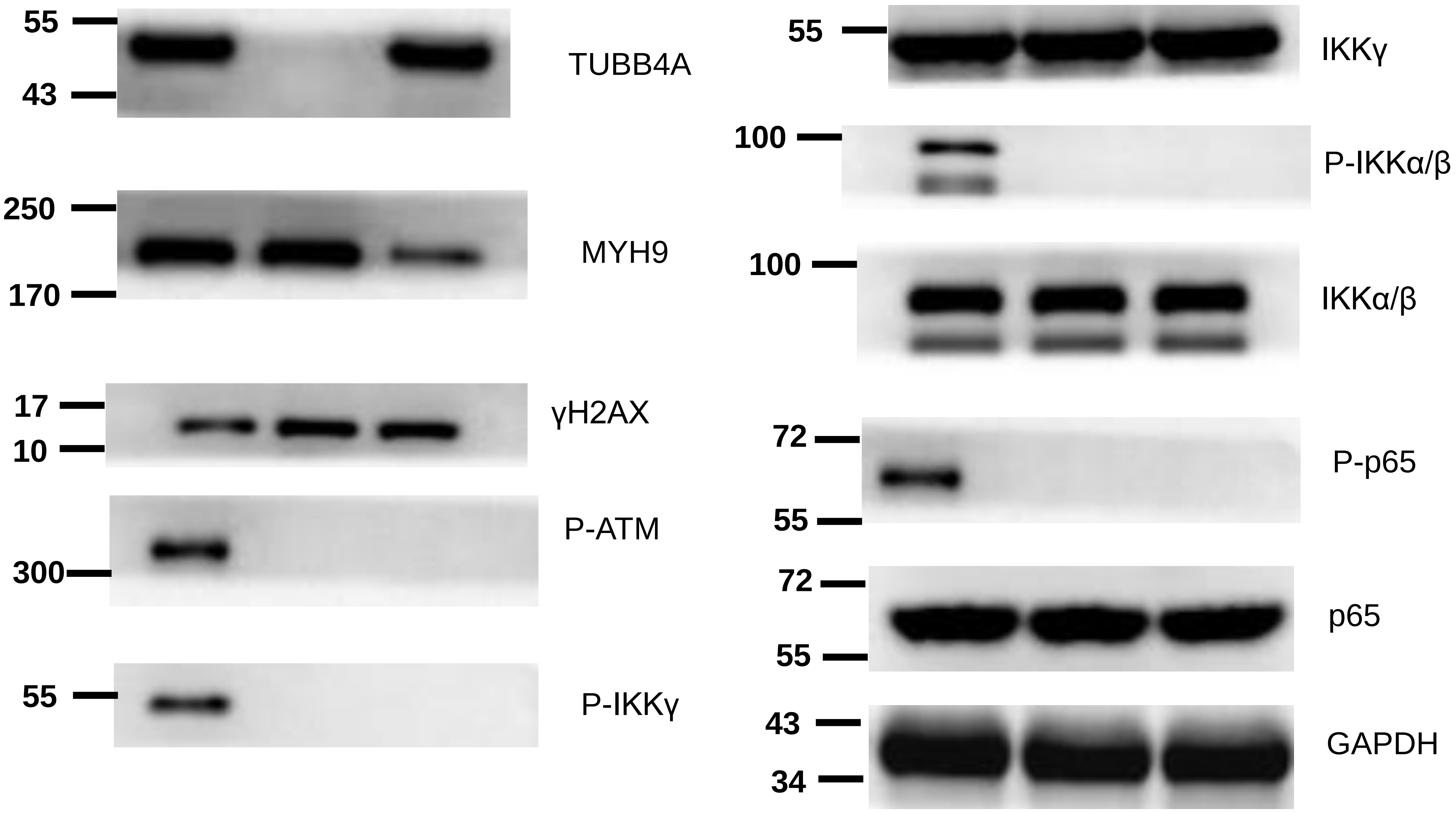
55
43
TUBB4A
250
170
MYH9
17
10
γH2AX
P-ATM
300
55
P-IKKγ
55
IKKγ
100
P-IKKα/β
100
IKKα/β
72
55
P-p65
72
55
p65
43
34
GAPDH

**Fig. 4I**

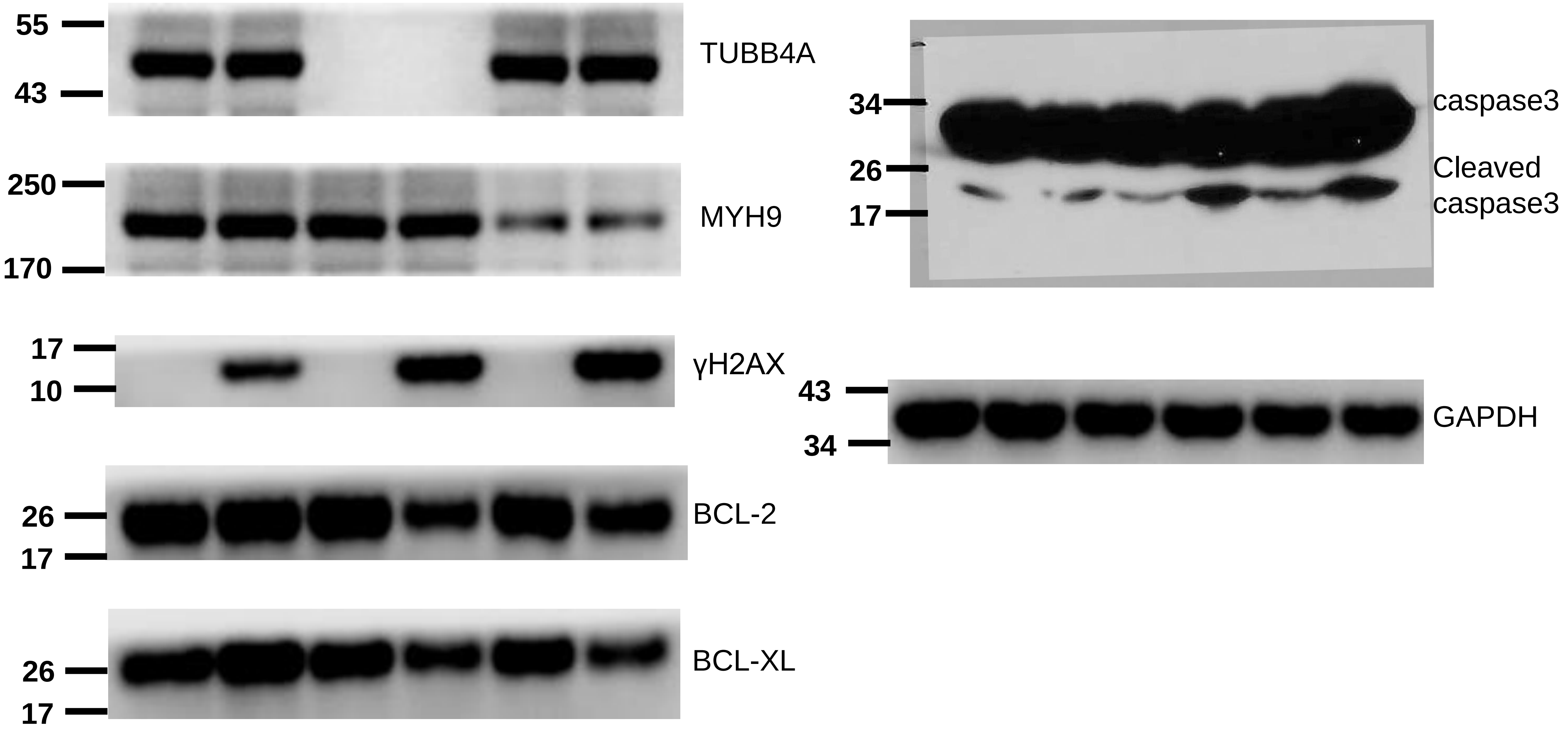
55
43
TUBB4A
250
170
MYH9
17
10
γH2AX
26
17
BCL-2
26
17
BCL-XL
34
26
17
caspase3
Cleaved
caspase3
43
34
GAPDH

Fig. 4J

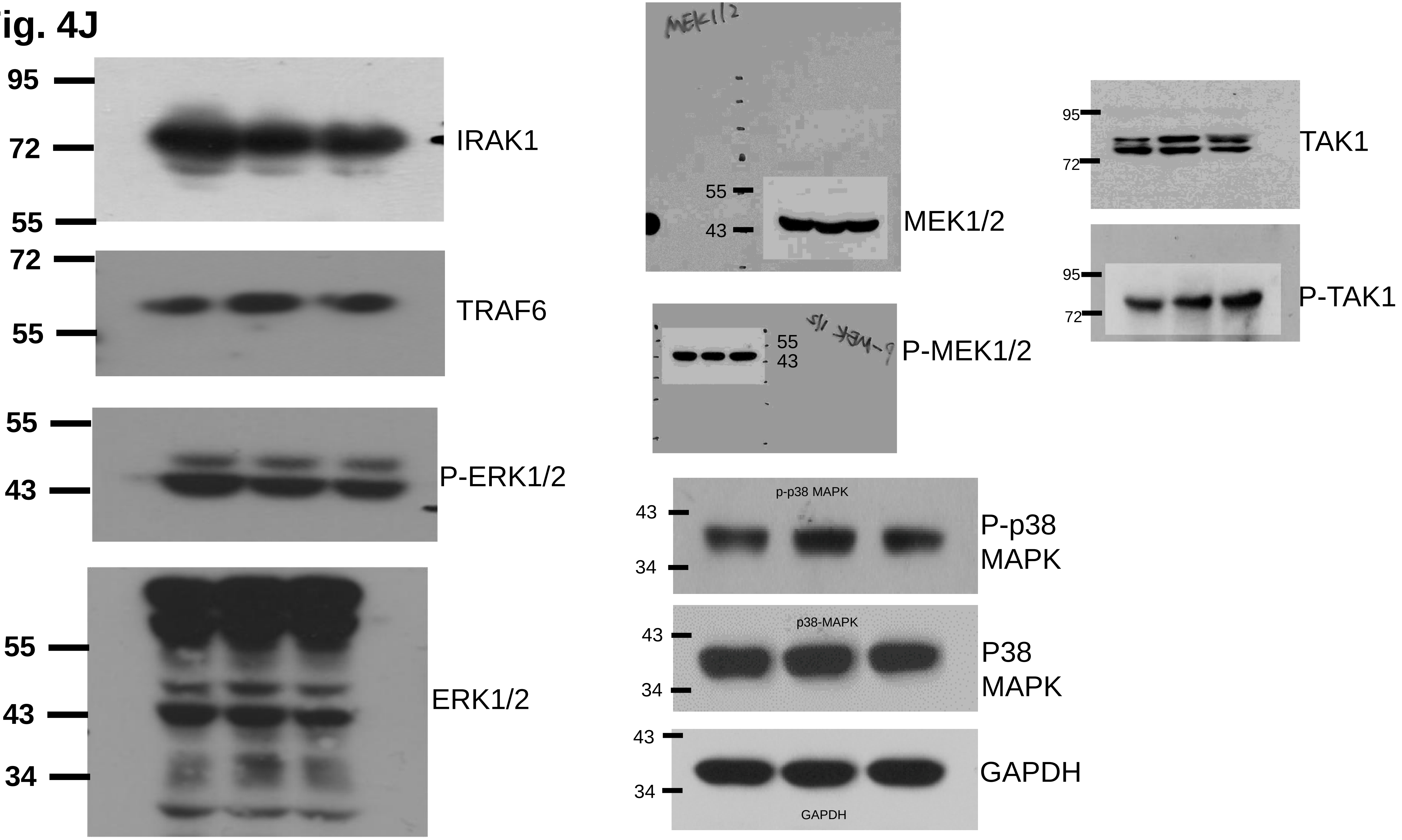
95
72
55
IRAK1
72
55
TRAF6
55
43
P-ERK1/2
55
43
34
ERK1/2
MEK1/2
55
43
MEK1/2
55
43
P-MEK1/2
43
p-p38 MAPK
34
P-p38 MAPK
43
p38-MAPK
34
P38 MAPK
43
34
GAPDH
GAPDH
95
72
TAK1
95
72
P-TAK1

**Fig. 5A**

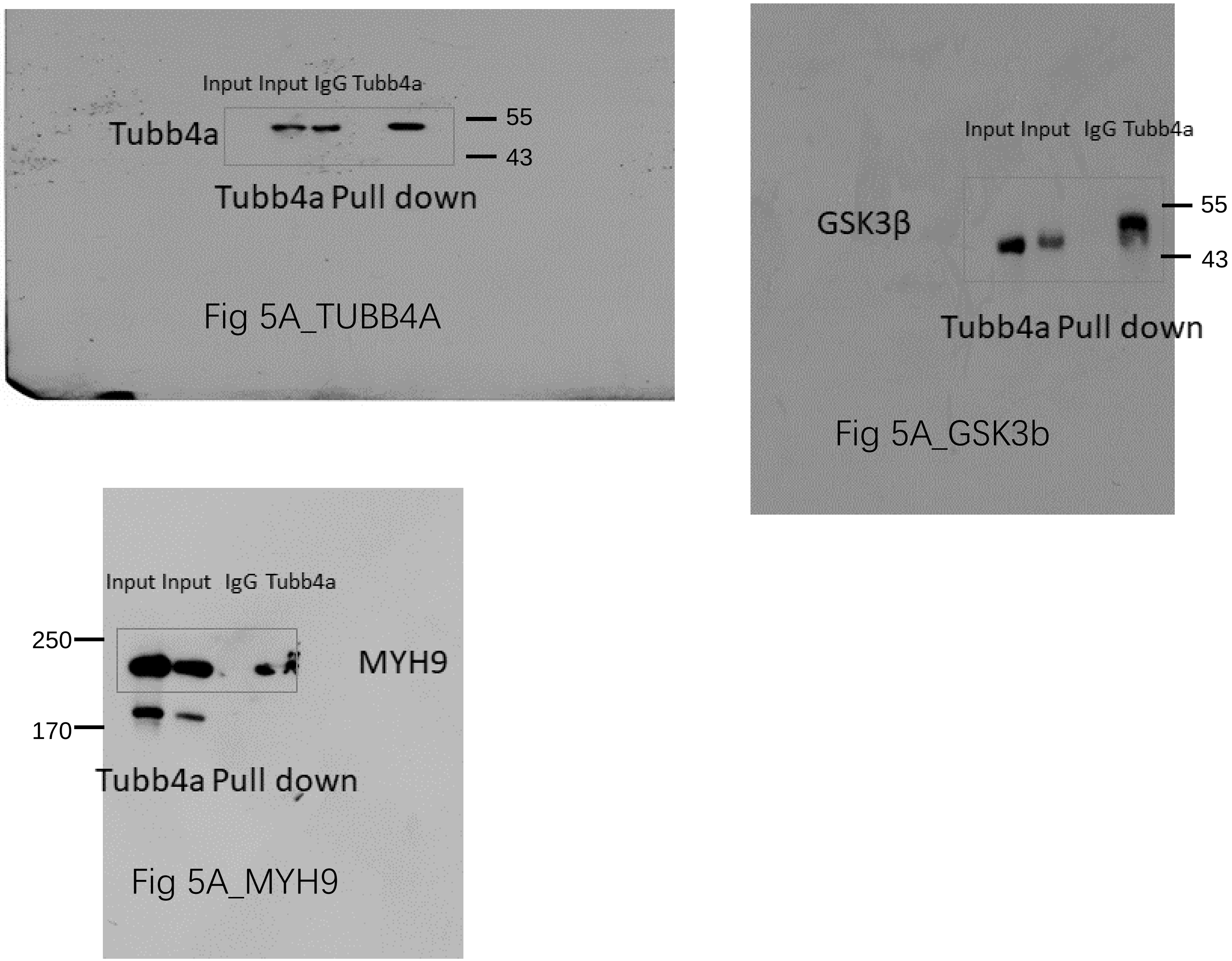
Input Input IgG Tubb4a
Tubb4a
55
43
Tubb4a Pull down
Fig 5A_TUBB4A
Input Input IgG Tubb4a
GSK3β
55
43
Tubb4a Pull down
Fig 5A_GSK3b
Input Input IgG Tubb4a
250
MYH9
170
Tubb4a Pull down
Fig 5A_MYH9

**Fig. 5B**

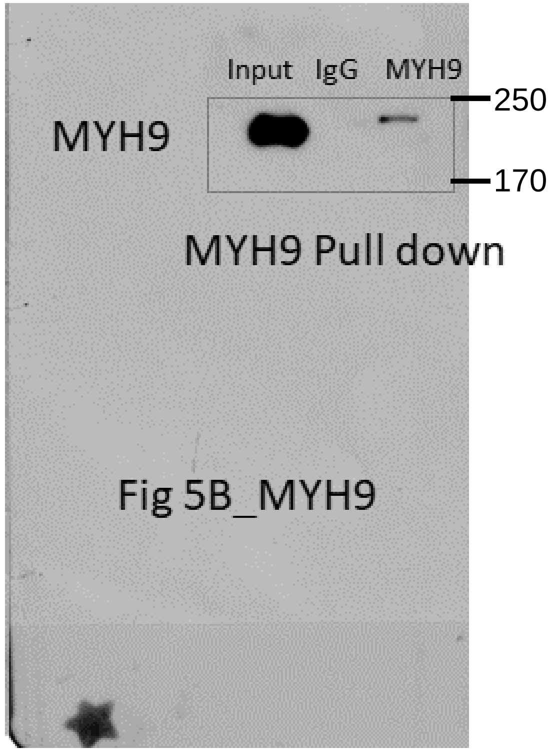


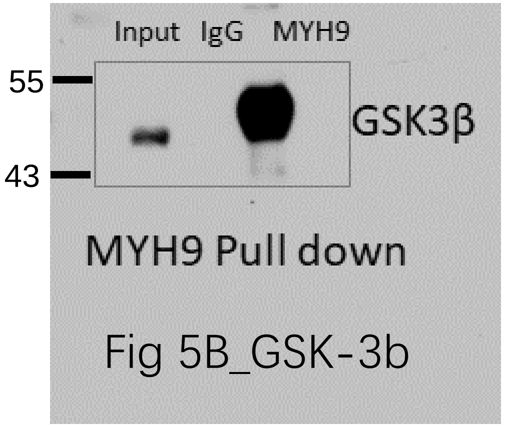


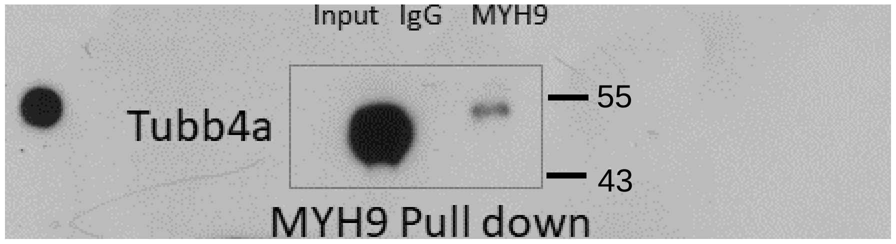


Fig 5B_TUBB4A

# Fig. 5C-D

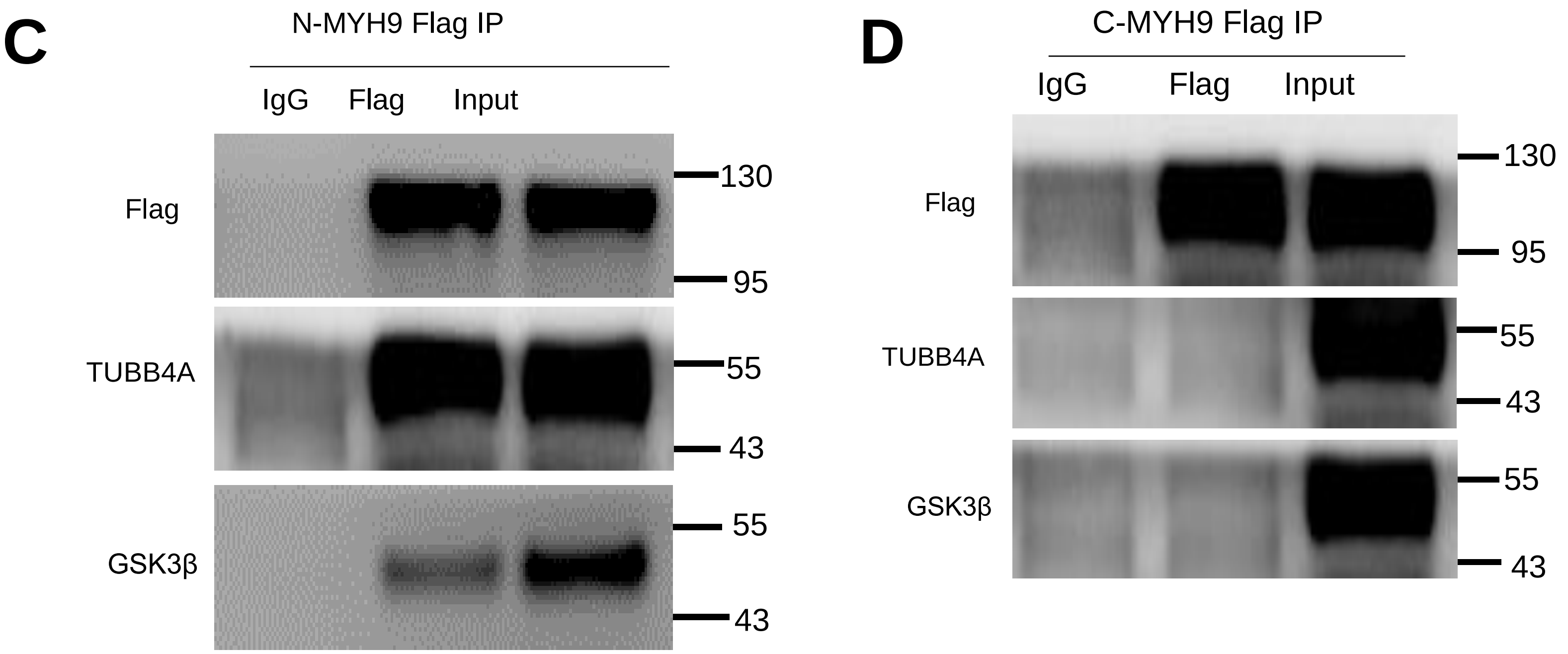
C
N-MYH9 Flag IP
IgG
Flag
Input
Flag
TUBB4A
GSK3β
130
95
55
43
55
43
D
C-MYH9 Flag IP
IgG
Flag
Input
Flag
TUBB4A
GSK3β
130
95
55
43
55
43

Fig. 5E

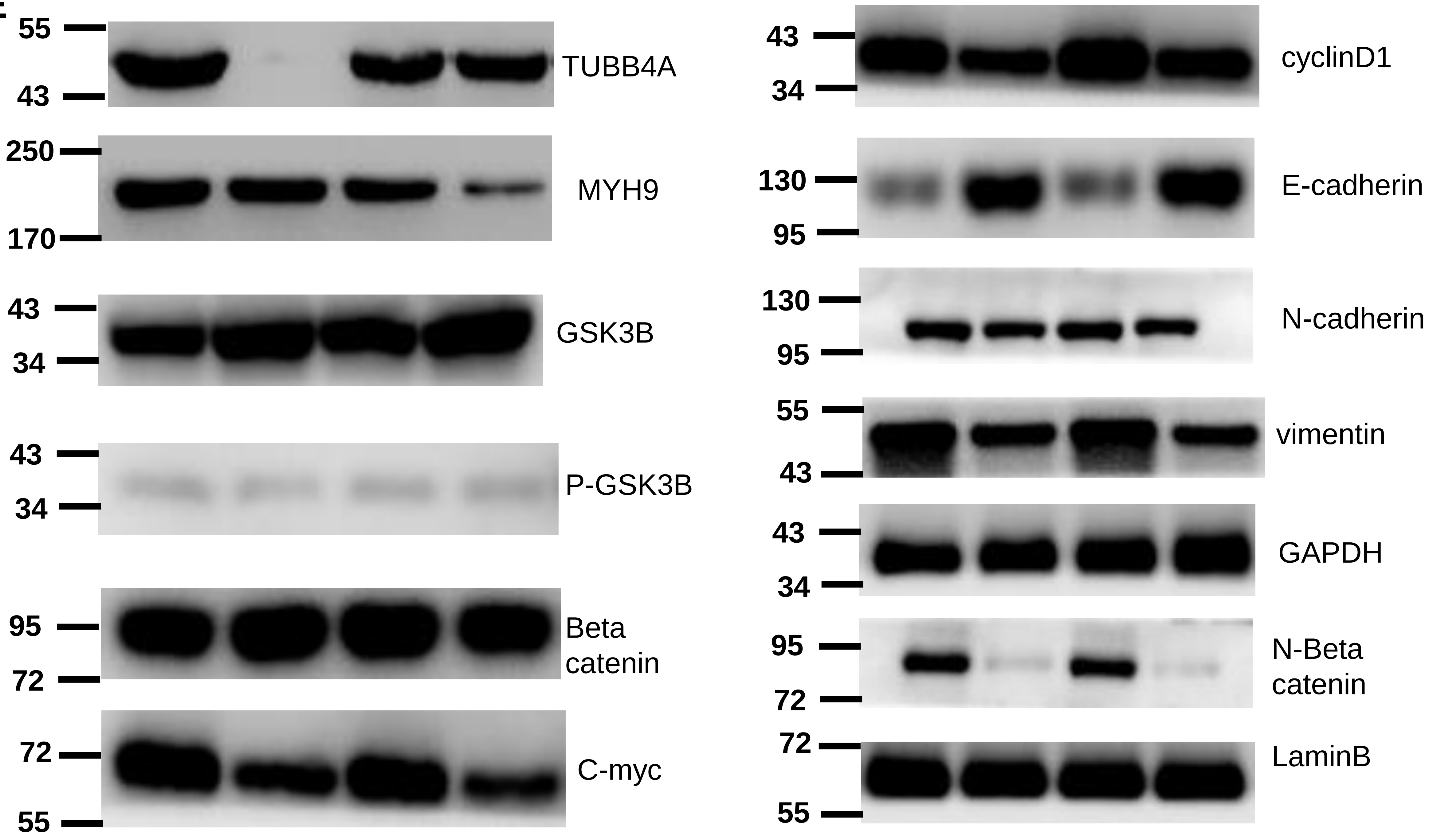
55
43
TUBB4A
250
170
MYH9
43
34
GSK3B
43
34
P-GSK3B
95
72
Beta catenin
72
55
C-myc
43
34
cyclinD1
130
95
E-cadherin
130
95
N-cadherin
55
43
vimentin
43
34
GAPDH
95
72
N-Beta catenin
72
55
LaminB

## Fig. 5F

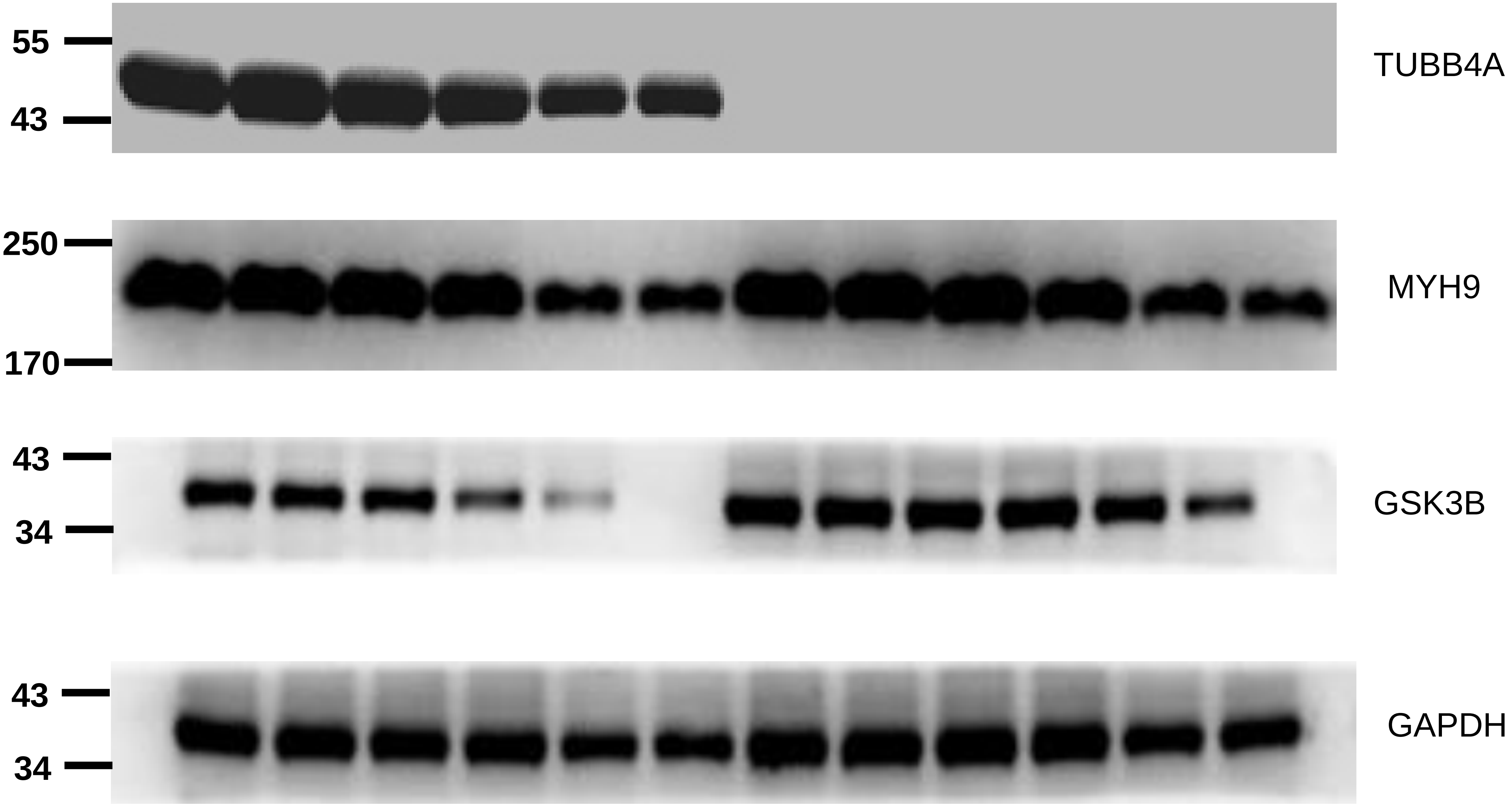

# Fig. 5G

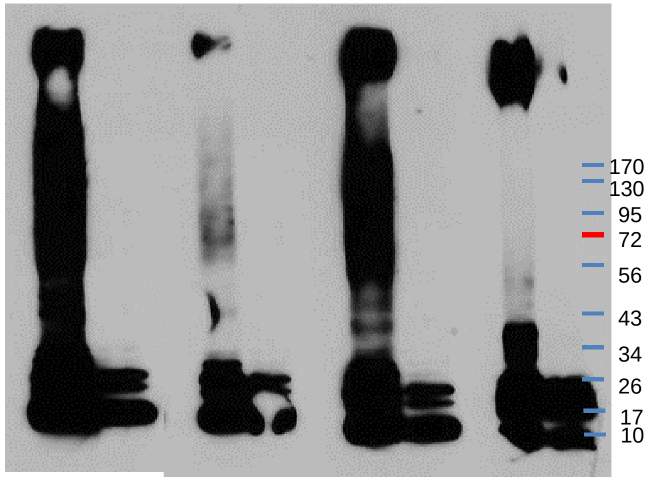


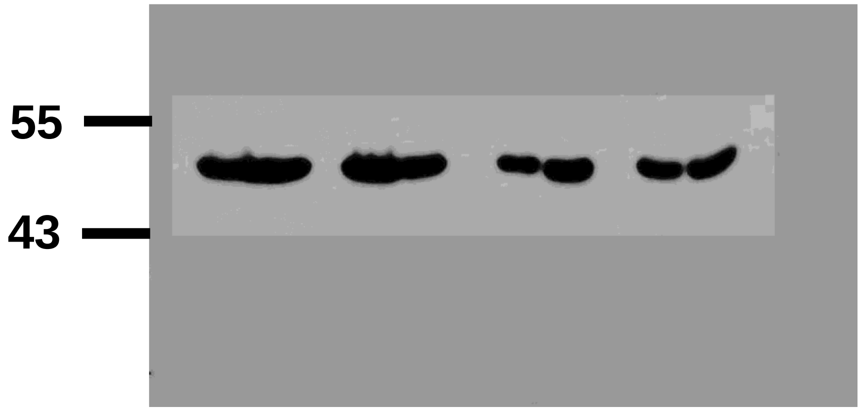


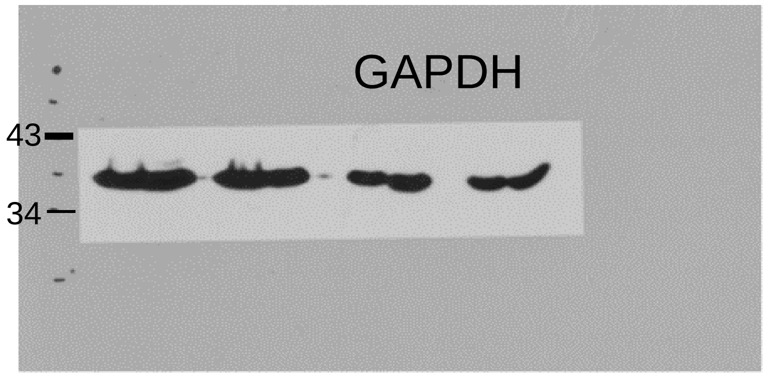

# Fig. S2A, 2E, 4A, 8G, 8I

## Fig S2A

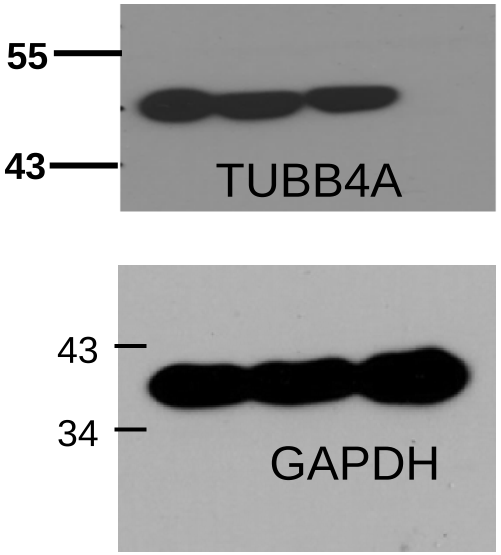


## Fig S2E

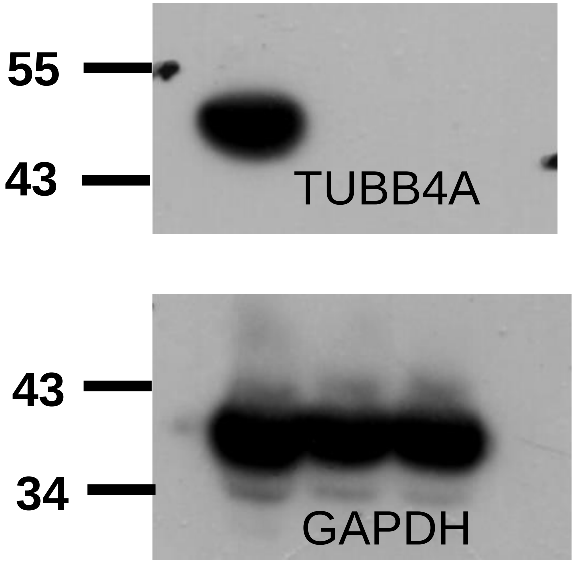


## Fig S8G

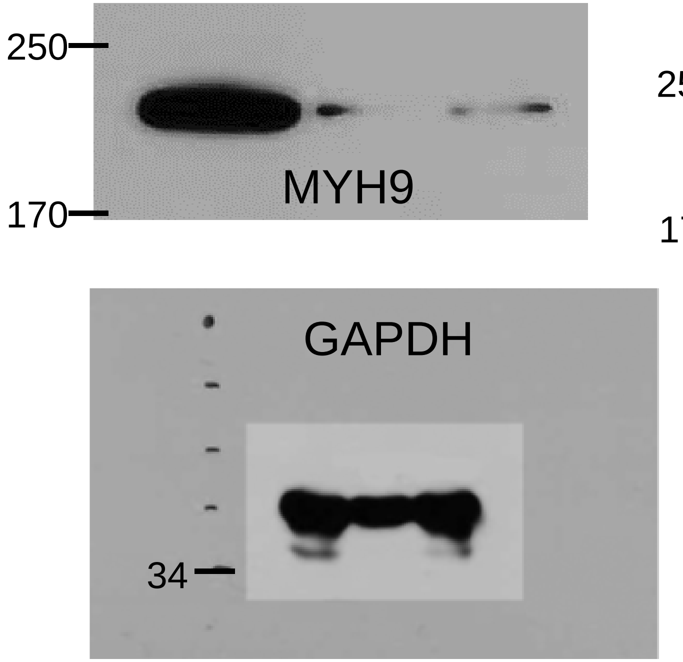


## Fig S8I

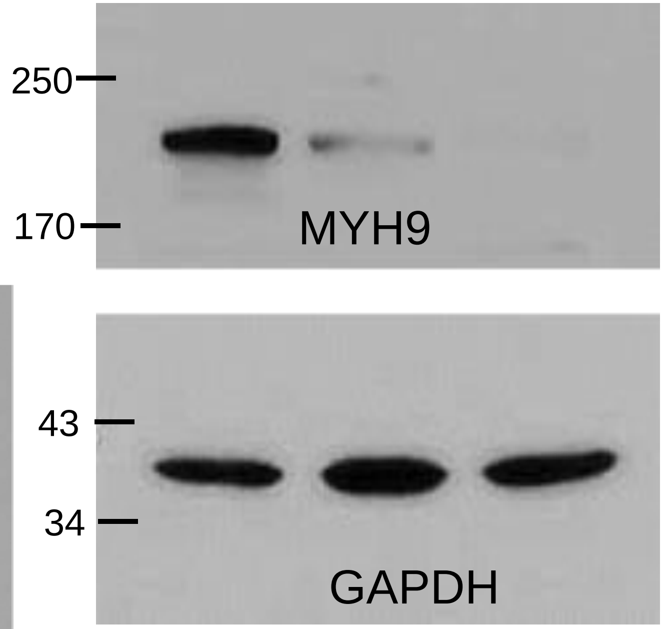


## Fig S4A

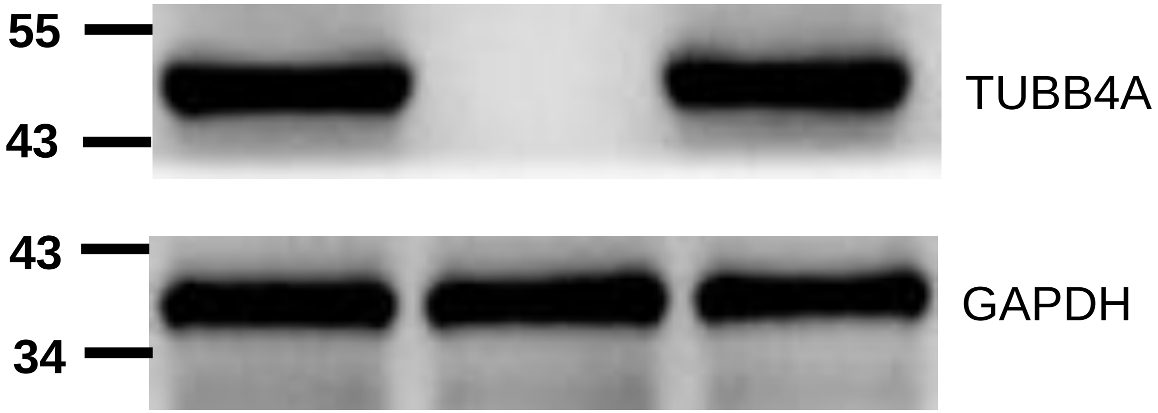

**Fig. S2G**

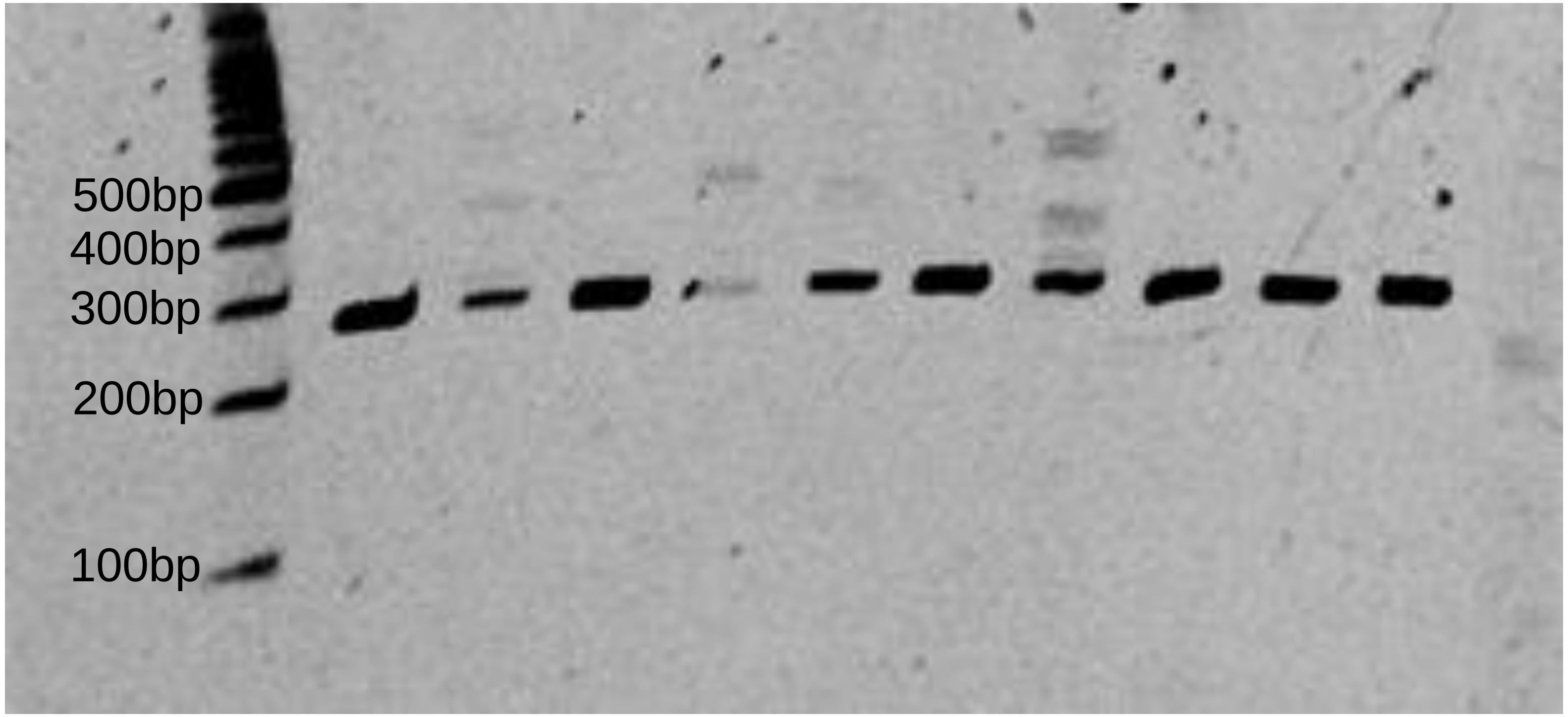

Supplement: Supplementary file 1 — Supplementary Information [file 41467_2022_30409_MOESM1_ESM.pdf]
