# Supplementary material for: TUBB4A interacts with MYH9 to protect the nucleus during cell migration and promotes prostate cancer via GSK3β/β-catenin signalling
Source: Nat Commun. 2022 May 19;13:2792. doi: 10.1038/s41467-022-30409-1 (PMC9120517; doi:10.1038/s41467-022-30409-1)
Supplement: Supplementary file 2 — Description of Additional Supplementary Information [file 41467_2022_30409_MOESM2_ESM.pdf]

Inventory of Supporting Information

Supplementary Figure S1. Characterization of *TUBB4A* expression profiling and genetic alteration in human primary prostate cancer.

Supplementary Figure S2. CRISPR-Cas9 *TUBB4A* knockout (KO) in prostate cancer cell lines.

Supplementary Figure S3. Cell movement and migration of scrambled and *TUBB4A* KO prostate cancer cell lines.

Supplementary Figure S4. Ectopic expression of TUBB4A rescues the *TUBB4A* KO-reduced proliferation and migration of prostate cancer cells.

Supplementary Figure S5. *TUBB4A* KO increases the cell area and the F-actin intensity in prostate cancer cells.

Supplementary Figure S6. *TUBB4A* KO does not change α-tubulin or lamin.

Supplementary Figure S7. TUBB4A-related signaling pathways and gene network.

Supplementary Figure S8. MYH9-related GSK3β/β-catenin signaling gene network and the effect of *MYH9* knockdown on growth of prostate cancer cells.

Supplementary Figure S9. Expression of EMT, DNA damage response and apoptotic markers in scrambled and *TUBB4A* KO DU145 xenograft tumors of NSG mice.

Supplementary Figure S10. *TUBB4A* KO reduces tumor colonization in the lungs of NSG mice.

Supplementary Figure S11. Establishment of genetically engineered spontaneous prostate cancer mouse models.

Supplementary Table S1. Human specimen characteristics

Supplementary Table S2. Mass spectrometry analysis for TUBB4A-interacting proteins

Supplementary Table S3. Specific primary antibodies used in this study

Supplementary Table S4. The sequences of primer, sgRNA, siRNA, and ORF used in this study

Supplementary Video 1. Automated live-cell two-dimensional random movement assay for scramble control PC3 cells.

Supplementary Video 2. Automated live-cell two-dimensional random movement assay for *TUBB4A* KO PC3 cells.

Supplementary Video 3. Automated live-cell two-dimensional random movement assay for scramble control DU145 cells.

Supplementary Video 4. Automated live-cell two-dimensional random movement assay for *TUBB4A* KO DU145 cells.

Supplementary Video 5. Automated live-cell two-dimensional scratch migration assay in scramble control DU145 cells.

Supplementary Video 6. Automated live-cell two-dimensional scratch migration assay in *TUBB4A* KO1 DU145 cells.

Supplementary Video 7. Automated live-cell two-dimensional scratch migration assay for *TUBB4A* KO2 DU145 cells.

Source data
