# Supplementary material for: TUBB4A interacts with MYH9 to protect the nucleus during cell migration and promotes prostate cancer via GSK3β/β-catenin signalling
Source: Nat Commun. 2022 May 19;13:2792. doi: 10.1038/s41467-022-30409-1 (PMC9120517; doi:10.1038/s41467-022-30409-1)
Supplement: Supplementary file 10 — Reporting Summary [file 41467_2022_30409_MOESM10_ESM.pdf]

nature portfolio

Corresponding author(s): Lizhong Wang

Last updated by author(s): Apr 7, 2022

Reporting Summary

Nature Portfolio wishes to improve the reproducibility of the work that we publish. This form provides structure for consistency and transparency in reporting. For further information on Nature Portfolio policies, see our Editorial Policies and the Editorial Policy Checklist.

Statistics

For all statistical analyses, confirm that the following items are present in the figure legend, table legend, main text, or Methods section.

n/a	Confirmed	
☐	☒	The exact sample size (*n*) for each experimental group/condition, given as a discrete number and unit of measurement
☐	☒	A statement on whether measurements were taken from distinct samples or whether the same sample was measured repeatedly
☐	☒	The statistical test(s) used AND whether they are one- or two-sided<br>*Only common tests should be described solely by name; describe more complex techniques in the Methods section.*
☒	☐	A description of all covariates tested
☐	☒	A description of any assumptions or corrections, such as tests of normality and adjustment for multiple comparisons
☐	☒	A full description of the statistical parameters including central tendency (e.g. means) or other basic estimates (e.g. regression coefficient) AND variation (e.g. standard deviation) or associated estimates of uncertainty (e.g. confidence intervals)
☐	☒	For null hypothesis testing, the test statistic (e.g. *F*, *t*, *r*) with confidence intervals, effect sizes, degrees of freedom and *P* value noted<br>*Give P values as exact values whenever suitable.*
☒	☐	For Bayesian analysis, information on the choice of priors and Markov chain Monte Carlo settings
☒	☐	For hierarchical and complex designs, identification of the appropriate level for tests and full reporting of outcomes
☒	☐	Estimates of effect sizes (e.g. Cohen's *d*, Pearson's *r*), indicating how they were calculated

Our web collection on statistics for biologists contains articles on many of the points above.

Software and code

Policy information about availability of computer code

Data collection	All data were collected on individual hard devices (e.g., SEAGATE Backup Plus for digital images). Immunohistochemistry, immunofluorescence, and Transwell migration images were acquired on Olympus microscope (BX43 plus EXCELITAS X-Cite 120 LED boost). Colony formation images were photographed using Canon digital camera (PowerShot SX500 IS). Cell migration videos were recorded using an intelligent full-automatic fluorescence imaging microscope (EVOS AUTOFL2 Automatic Imaging System). Cell death rate after migration in 3D collagen gels was photographed using AMG microscope (EVOS xl Core AMEX-1000). Cell growth and scratch migration were measured by live cell kinetic imaging with walk-away automation using a Lionheart™ FX cell imager (Agilent Technologies). Western blots were imaged with ChemiDoc MP Imaging System (Bio-Rad) or Mini Med 90 Processor (AFP).
Data analysis	- All imaging data were processed using Adobe Photoshop CS2 (version 9.0.2).<br>- All data were processed using Microsoft Excel (Version 16)<br>- Statistical analyses were performed with Graph Pad Prism (Version 8.4.3), IBM SPSS (Version 25), or SAS (Version 9.4).

For manuscripts utilizing custom algorithms or software that are central to the research but not yet described in published literature, software must be made available to editors and reviewers. We strongly encourage code deposition in a community repository (e.g. GitHub). See the Nature Portfolio guidelines for submitting code & software for further information.

Data

Policy information about availability of data

All manuscripts must include a data availability statement. This statement should provide the following information, where applicable:

- Accession codes, unique identifiers, or web links for publicly available datasets
- A description of any restrictions on data availability
- For clinical datasets or third party data, please ensure that the statement adheres to our policy

The authors declare that all data supporting the finding of this study are available within the paper and its supplement data source file.
Analysis of RNA expression and DNA methylation of our work is in part based upon data generated by the TCGA dataset (www.cancer.gov/tcga).
Genetic analysis was performed using cBioPortal (www.cbioportal.org).
Survival analysis with gene expression from the TCGA dataset was performed using GEPIA (gepia.cancer-pku.cn).
The full mass spectrometry results of pull-down assay in excel form and raw data are presented in Supplementary Table 2 and Source Data file.

Field-specific reporting

Please select the one below that is the best fit for your research. If you are not sure, read the appropriate sections before making your selection.

☒ Life sciences ☐ Behavioural & social sciences ☐ Ecological, evolutionary & environmental sciences

For a reference copy of the document with all sections, see nature.com/documents/nr-reporting-summary-flat.pdf

Life sciences study design

All studies must disclose on these points even when the disclosure is negative.

Sample size	Sample size was determined according to previously conducted experiments (Wu et al. Cancer Res. 2019, 79:1413-25; Liu et al. Cancer Res. 2015, 75: 1714-24; Wang et al, Nat Commun. 2015, 6: 5909) and determined to be adequate based on the magnitude and consistency of measurable differences between groups. The number of experiments (n) is indicated in each figure legend.
Data exclusions	No data were excluded from the analysis.
Replication	Each of the in vitro findings has been repeated at least 3 times to ensure reproducibility and each of the in vivo findings has been repeated at least 2 times. All attempts of replication were successful. Number of times experiments were repeated can be found in the figure legends.
Randomization	For experiments with cell line, no randomization was required because the experimental cells came from the same source and cells within a monoclonal cell line do not exhibit genetic and phenotypic variation. However, cells were randomized into wells for various treatment groups to reduce technical variation between wells.<br><br>For in vivo experiments, animals were randomly allocated to control and treatment groups to minimize individual differences between groups. For different genotype groups, the numbers are the same and the average weight and age of mice are similar.
Blinding	Investigators were blinded during tissue collection and processing. Data analyses were performed by a biostatistician who was blinded to the experimental groups.<br><br>For immunohistochemical analysis, samples were blindly examined by pathologists. The investigators were blinded to group allocation during data collection and analysis.<br><br>Experiments based on cell cultures were not performed blindly because investigators need to know the cell source in order to allow for proper controls to be present in each experiment. However, investigators were blinded during the data analysis for these experiments.

Reporting for specific materials, systems and methods

We require information from authors about some types of materials, experimental systems and methods used in many studies. Here, indicate whether each material, system or method listed is relevant to your study. If you are not sure if a list item applies to your research, read the appropriate section before selecting a response.

Materials & experimental systems

n/a	Involved in the study
☐	☒ Antibodies
☐	☒ Eukaryotic cell lines
☒	☐ Palaeontology and archaeology
☐	☒ Animals and other organisms
☒	☐ Human research participants
☒	☐ Clinical data
☒	☐ Dual use research of concern

Methods

n/a	Involved in the study
☒	☐ ChIP-seq
☒	☐ Flow cytometry
☒	☐ MRI-based neuroimaging

Antibodies

Antibodies used

53BP1 (Cell Signaling Technology #4937), alpha-tubulin (DM1A) (Sigma-Aldrich #T6199), Androgen Receptor (441) (Santa Cruz Biotechnology #sc-7305), CK5 (EP1601Y) (Abcam #ab52635), c-MYC (Y69) (Abcam #ab32072), CyclinD1 (EPR2241) (Abcam #ab134175), E-cadherin (M168) (Abcam ab76055), GAPDH (D16H11) (Cell Signaling Technology #5174), Goat-anti mouse IgG (H&L Alex 488) (Abcam #ab150117), Goat-anti mouse IgG (H&L Alex 568) (Abcam #ab175473), IKKα (Cell Signaling Technology #2682), IKKγ (Cell Signaling Technology #2685), IRAK1 (Abcam #ab238), Ki67 (Abcam #ab15580), Lamin A/C (E-1) (Santa Cruz Biotechnology #sc-376248), MYH9 (Proteintech Rosemont #14844-1-AP), N-cadherin (Abcam #ab18203), NF-κB p65 (A-12) (Santa Cruz Biotechnology #sc-514451), NF-κB p-p65 (S536) (Abcam #ab86299), NF-κB p-p65 (S536) (Sigma-Aldrich #SAB5700363), p38 MAPK (Cell Signaling Technology #9212), p44/42 MAPK (137F5) (Cell Signaling Technology #4695), p-IKKα/β (Ser176/180) (Cell Signaling Technology #2697), p-IKKγ (Ser376) (Cell Signaling Technology #2689), p-p38 MAPK (Thr180/Tyr182) (Cell Signaling Technology #9211), p-p44/42 MAPK (Thr202/Tyr204) (Cell Signaling Technology #4376), TRAF6 (D21G3) (Cell Signaling Technology #8028), TUBB4A (EPR16776) (Abcam #ab179509), TUBB4A (ONS.1A6) (Abcam #ab11315), Vimentin (EPR3776) (Abcam #ab92547), γH2AX (20E3 Rb) Cell Signaling Technology #9718), ATM (G12) (Santa Cruz Biotechnology #sc-377293), p-ATM (Ser1981) [EP1890Y] (Abcam #ab81292), BCL-2 (C-2) (Santa Cruz Biotechnology #sc-7382), BCL-XL (54H6) (Cell Signaling Technology # 2764P), Caspase3 (31A1067) (Santa Cruz Biotechnology #sc-56053), MEK1/2 (Proteintech Rosemont #11049-1-AP), p-MEK1/2 (Ser217/221) (Cell Signaling Technology #91218), TAK1 (Proteintech Rosemont #12330-2-AP), p-TAK1 (Proteintech Rosemont # 28958-1-AP), GSK3 beta (27C10) (Cell Signaling Technology # 9315), p-GSK3 beta (Ser9) (Cell Signaling Technology # 5558S), β-catenin (BD Biotechnology # 610154), Lamin B (B10) (Santa Cruz Biotechnology #sc-374015), TUNEL (ABP Biosciences #A050), and Alexa Fluor® 568 phalloidin (for F-actin) (Thermo Fisher Scientific #A12380).

Validation

Antibodies validated through gene knockout and cellular localization using Western blot, immunofluorescence, and immunohistochemistry: HumanTUBB4A (EPR16776) (Abcam #ab179509) and HumanTUBB4A (ONS.1A6) (Abcam #ab11315).

Antibodies validated through siRNA knockdown using Western blot: Human MYH9 (Proteintech Rosemont #14844-1-AP), Human c-MYC (Y69) (Abcam #ab32072), and Human CyclinD1 (EPR2241) (Abcam #ab134175).

Antibodies validated using DNA damage reagents and cellular localization using immunofluorescence: Human γH2AX (20E3 Rb) (Cell Signaling Technology #9718) and Human 53BP1 (Cell Signaling Technology #4937).

Antibodies validated using Western blots (band size and siRNA knockdown): Human γH2AX (20E3 Rb) (Cell Signaling Technology #9718), Human alpha-tubulin (DM1A) (Sigma-Aldrich #T6199), Human GAPDH (D16H11) (Cell Signaling Technology #5174), Human IKKα (Cell Signaling Technology #2682), Human IKKγ (Cell Signaling Technology #2685), Human IRAK1 (Abcam #ab238), Human p38 MAPK (Cell Signaling Technology #9212), Human p44/42 MAPK (137F5) (Cell Signaling Technology #4695), Human E-cadherin (M168) (Abcam ab76055), Human N-cadherin (Abcam #ab18203), Human p-IKKα/β (Ser176/180) (Cell Signaling Technology #2697), Human p-IKKγ (Ser376) (Cell Signaling Technology #2689), Human p-p38 MAPK (Thr180/Tyr182) (Cell Signaling Technology #9211), Human p-p44/42 MAPK (Thr202/Tyr204) (Cell Signaling Technology #4376), Human NF-κB p65 (A-12) (Santa Cruz Biotechnology #sc-514451), Human TRAF6 (D21G3) (Cell Signaling Technology #8028), Human vimentin (EPR3776) (Abcam #ab92547), Human ATM (G12) (Santa Cruz Biotechnology #sc-377293), Human p-ATM (Ser1981) [EP1890Y] (Abcam #ab81292), Human BCL-2 (C-2) (Santa Cruz Biotechnology #sc-7382), Human BCL-XL (54H6) (Cell Signaling Technology # 2764P), Human Caspase3 (31A1067) (Santa Cruz Biotechnology #sc-56053), Human MEK1/2 (Proteintech Rosemont #11049-1-AP), Human p-MEK1/2 (Ser217/221) (Cell Signaling Technology #9121), Human TAK1 (Proteintech Rosemont #12330-2-AP), Human p-TAK1 (Proteintech Rosemont # 28958-1-AP), Human GSK3 beta (27C10) (Cell Signaling Technology # 9315), Human p-GSK3 beta (Ser9) (Cell Signaling Technology # 5558S), Human β-catenin (BD Biotechnology # 610154), Human Lamin B (B10) (Santa Cruz Biotechnology #sc-374015).

Antibodies validated using Western blots (band size) and immunohistochemistry (cellular localization): Mouse Androgen Receptor (441) (Santa Cruz Biotechnology #sc-7305), Mouse CK5 (EP1601Y) (Abcam #ab52635), Human E-cadherin (M168) (Abcam ab76055), Mouse and Human Ki67 (Abcam #ab15580), Human NF-κB p-p65 (S536) (Abcam #ab86299), Mouse NF-κB p-p65 (S536) (Sigma-Aldrich #SAB5700363), Mouse and Human p-IKKα/β (Ser176/180) (Cell Signaling Technology #2697), and Mouse and Human TUBB4A (EPR16776) (Abcam #ab179509).

Antibodies validated using immunohistochemistry (cellular localization) with positive and negative controls: Mouse and Human Ki67 (Abcam #ab15580).

Antibodies validated using immunofluorescence with positive and negative controls: TUNEL (ABP Biosciences #A050) and Alexa Fluor® 568 phalloidin (for F-actin) (Thermo Fisher Scientific #A12380).

Eukaryotic cell lines

Policy information about cell lines

Cell line source(s)	Prostate cancer cell lines PC3, DU145 and LNCaP were purchased from the ATCC. DU145-luc cells were acquired from the JCRB Cell Bank (Japan).
Authentication	PC3, DU145 and LNCaP were validated using RNA-seq after ordering. DU145-luc was authenticated by JCRB Cell Bank using DNA Profile (STR) analysis.
Mycoplasma contamination	All cell lines are tested for mycoplasma both upon arrival at UAB and after a new stock of cells is made, and all of them resulted to be negative for mycoplasma contamination.
Commonly misidentified lines (See ICLAC register)	No commonly-misidentified cell lines were used in this study.

Animals and other organisms

Policy information about studies involving animals; ARRIVE guidelines recommended for reporting animal research

Laboratory animals	NOD-scid IL2rgnull (NSG) immunodeficient male mice (The Jackson Laboratory) at 6 weeks of age were used to generate xenograft animal models. Xenograft tumor growth and metastasis were monitored up to 50 or 90 days.<br><br>Tubb4a and Pten floxed mice (The Jackson Laboratory) were crossed to Nkx3-1CreERT2 knock-in mice (National Cancer Institute Mouse Model Deposit) expressing Cre cDNA under inducible control of the tamoxifen on a C57BL/6 background. We generated prostate Tubb4a, Pten, or both conditional knockout (cKO, Nkx3-1CreERT2/- × Tubb4aflox/flox, Nkx3-1CreERT2/- × Ptenflox/flox, Nkx3-1CreERT2/- × Tubb4aflox/flox × Ptenflox/flox) male mice on on a C57BL/6 background. Also, Nkx3-1CreERT2/- × Tubb4aflox/flox mice were crossed to TRAMP mice (The Jackson Laboratory) on a C57BL/6 background. We generated prostate Tubb4a cKO TRAMP male mice (Nkx3-1CreERT2/- × Tubb4aflox/flox × TRAMP) male mice on a C57BL/6 background. Mice at 8 weeks of age were used for tumor development and metastasis of spontaneous prostate cancers up to one year.<br><br>Mice were housed in the University of Alabama at Birmingham Animal Resources Program with 12 hour light/dark cycles, 65-75 degrees Fahrenheit, 40-60% humidity, and on site veterinarian care.
Wild animals	The study did not involve the use of wild animals.
Field-collected samples	The study did not involve the use of collected from the field.
Ethics oversight	The protocol and procedures were approved by the Institutional Animal Care and Use Committee (IACUC) of University of Alabama at Birmingham. The studies were in compliance with all ethical regulations. Because prostate cancer occurs only in men, male mice were used in this study.

Note that full information on the approval of the study protocol must also be provided in the manuscript.
