# Supplementary material for: TUBB4A interacts with MYH9 to protect the nucleus during cell migration and promotes prostate cancer via GSK3β/β-catenin signalling
Source: Nat Commun. 2022 May 19;13:2792. doi: 10.1038/s41467-022-30409-1 (PMC9120517; doi:10.1038/s41467-022-30409-1)
Supplement: Supplementary file 11 — Source Data [file 41467_2022_30409_MOESM11_ESM.zip › source-data/Figure 1/Fig. 1F.pdf]

PATHOLOGY

PROSTATE CANCER

GENE/PROTEIN

ANTIBODIES AND VALIDATION

Dictionary

Prostate cancer

Human pathology

Prostate cancer

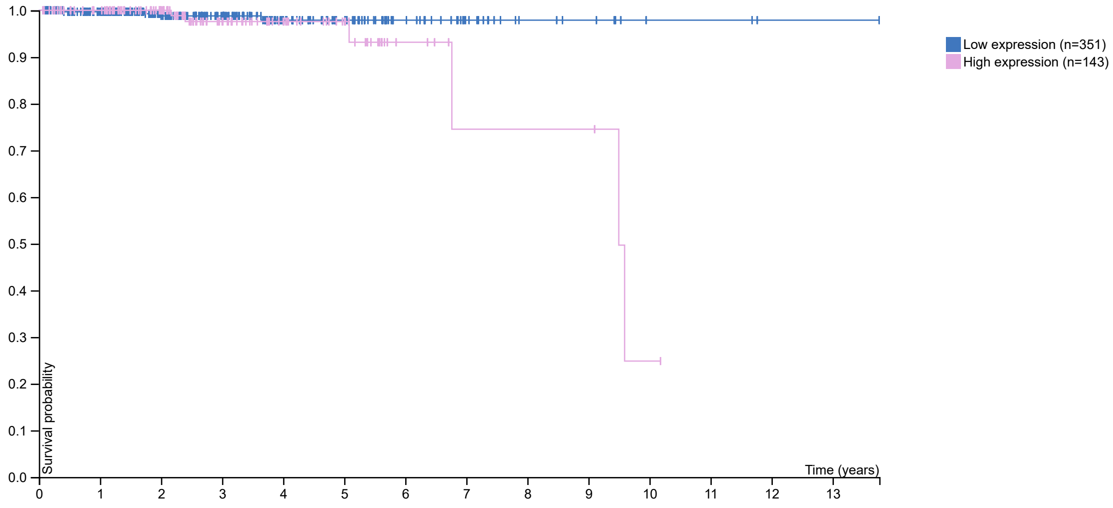

TCGA RNA samples

**Average FPKM**	1.2		
**Number of samples**	494		
	**Sample**	**Description**	**FPKM**
**Samples**	TCGA-V1-A9Z7-01A	55 years, male, alive, 874 days	28.7
	TCGA-EJ-5494-01A	50 years, male, white, alive, 1477 days	26.2
	TCGA-M7-A71Z-01A	62 years, male, alive, 643 days	20.7
	TCGA-G9-6498-01A	53 years, male, alive, 1952 days	20.2
	TCGA-YJ-A8SW-01A	male, alive, 148 days	18.8
	TCGA-CH-5752-01A	66 years, male, white, alive, 943 days	16.5
	TCGA-J4-A6M7-01A	53 years, male, alive, 512 days	10.8
	TCGA-YL-A8SP-01B	58 years, male, alive, 2366 days	10.7
	TCGA-G9-A9S4-01A	62 years, male, alive, 906 days	9.9

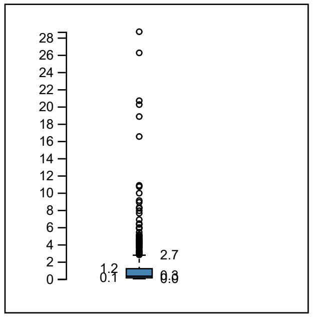

Show all

PROSTATE CANCER - Protein expression

Antibody staining	Antibody HPA043640	Antibody HPA046280	Antibody CAB010768
▲ Staining			
High			
Medium			
Low			
Not detected			
Intensity ▼			

We use cookies to enhance the usability of our website. If you continue, we'll assume that you are happy to receive all cookies. More information. Don't show this again.
