# Supplementary figures and images for: TUBB4A interacts with MYH9 to protect the nucleus during cell migration and promotes prostate cancer via GSK3β/β-catenin signalling
Source: Nat Commun. 2022 May 19;13:2792. doi: 10.1038/s41467-022-30409-1 (PMC9120517; doi:10.1038/s41467-022-30409-1)

Fig. 2A, 2E, 2I

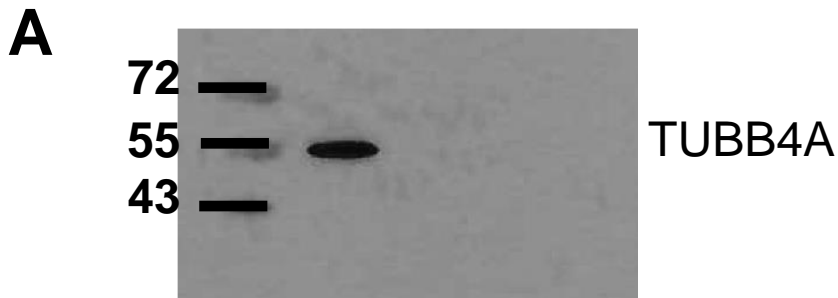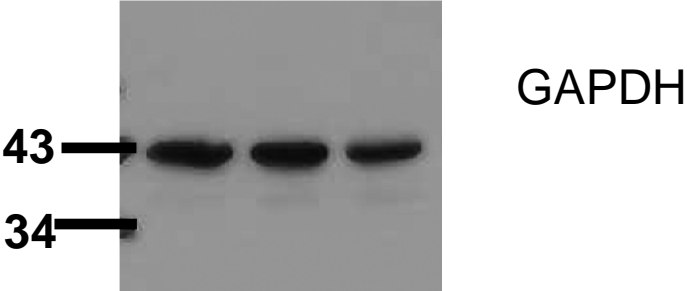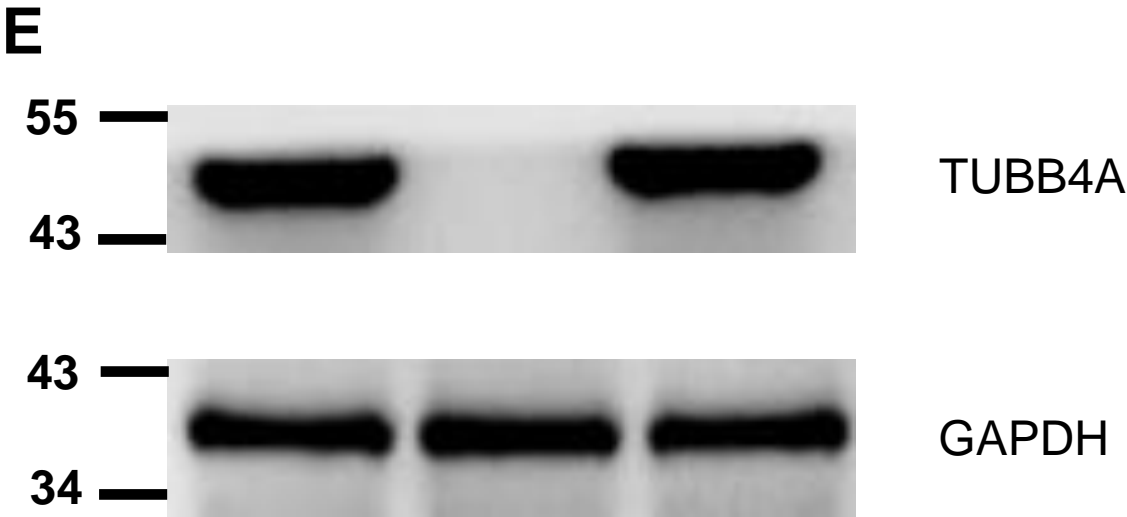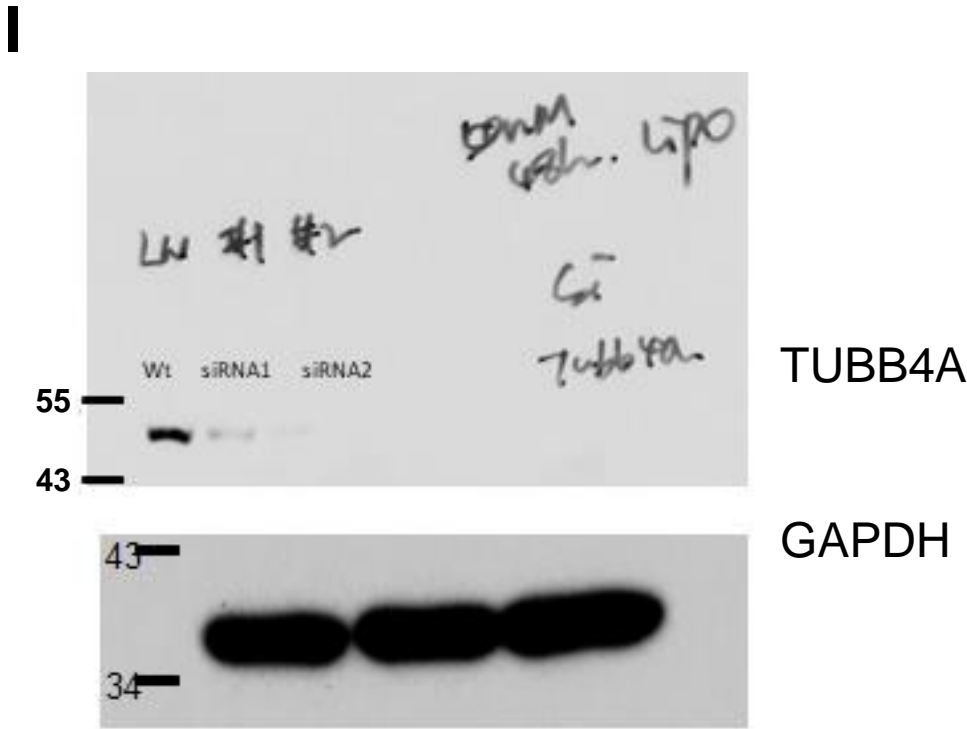

Supplement: Supplementary file 11 — Source Data [file 41467_2022_30409_MOESM11_ESM.zip › source-data/Figure 2/Fig. 2A, 2E, 2I.pdf]

**Fig. 4C**

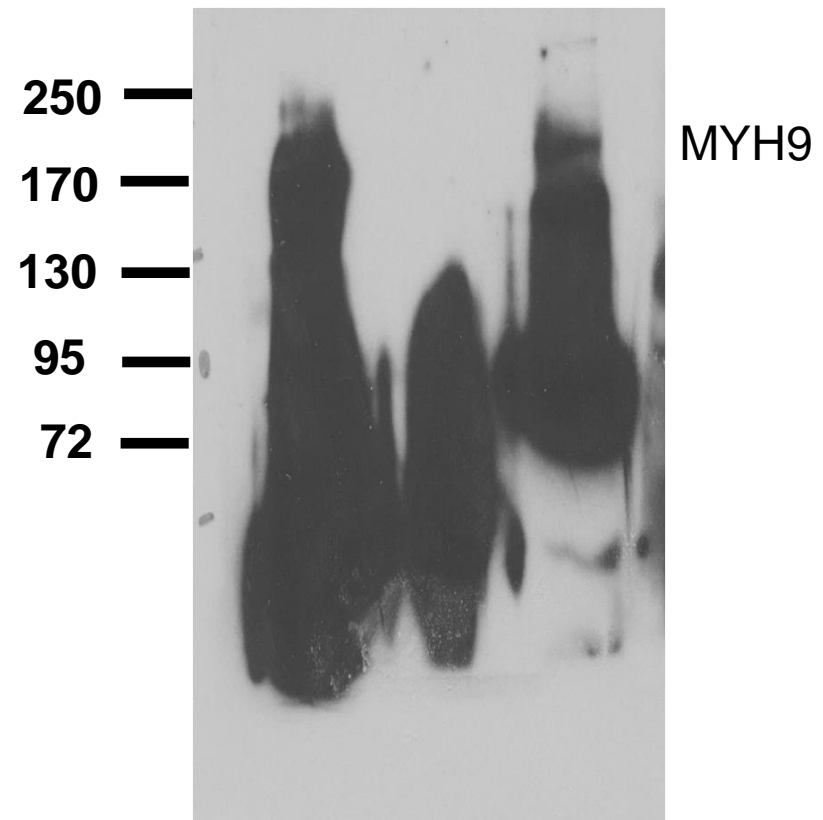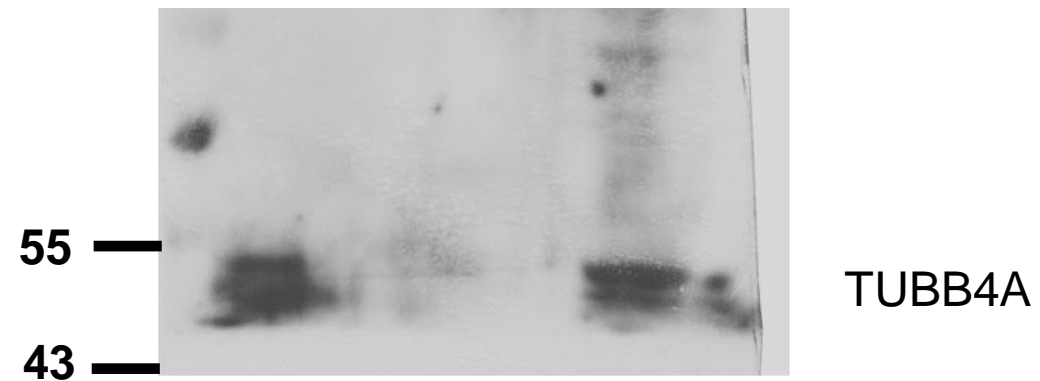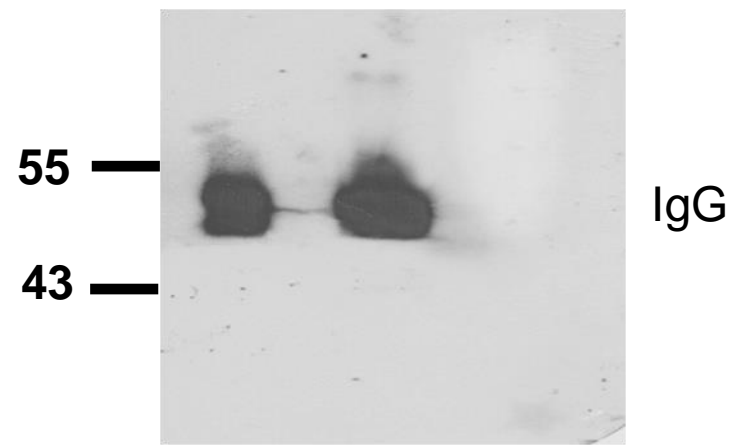

**Fig. 4D**

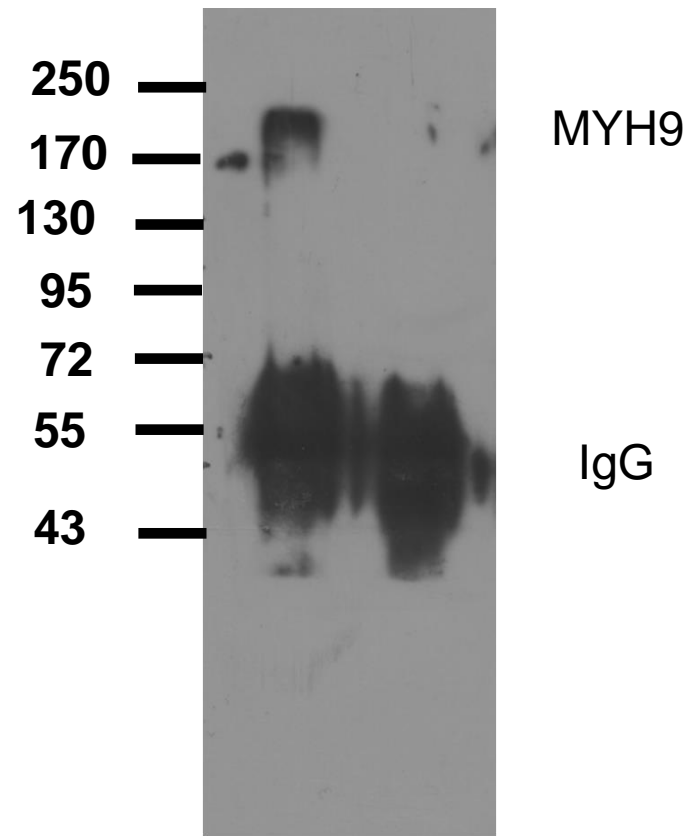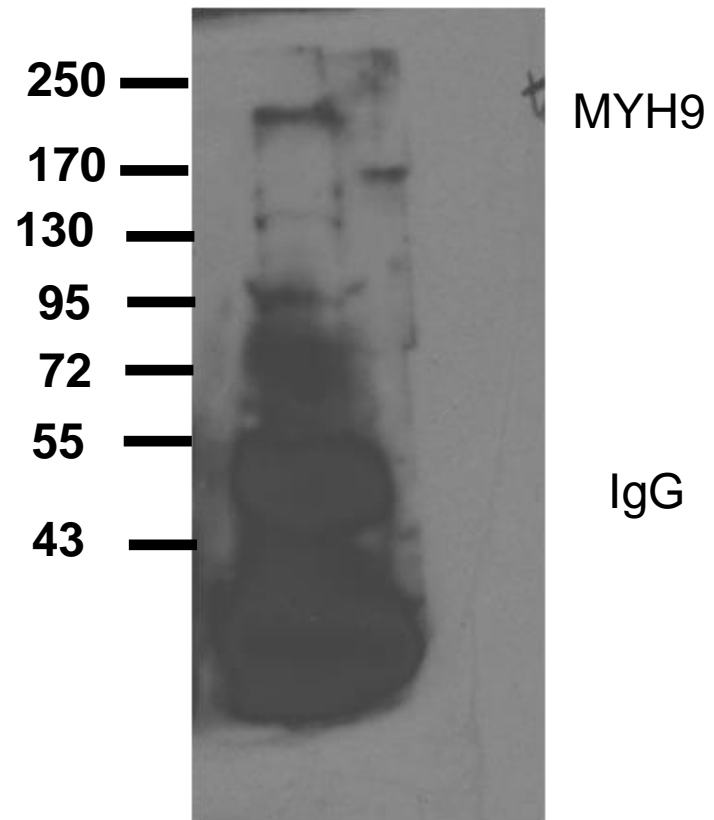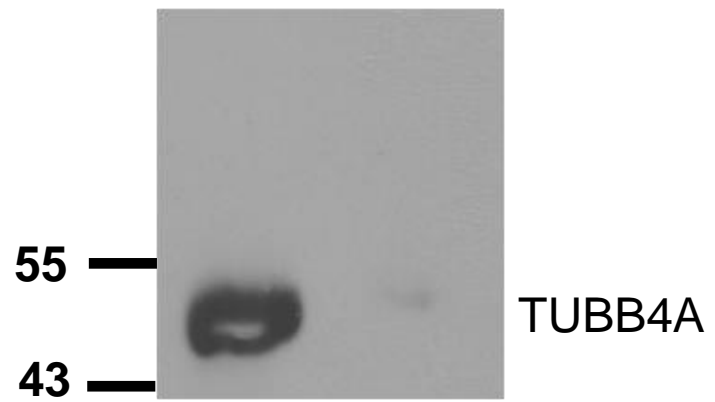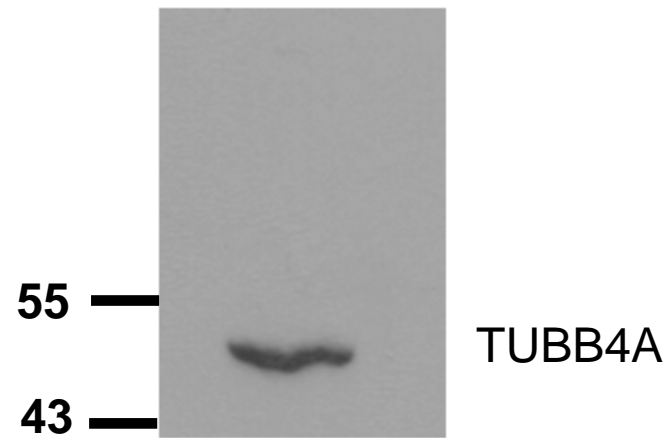

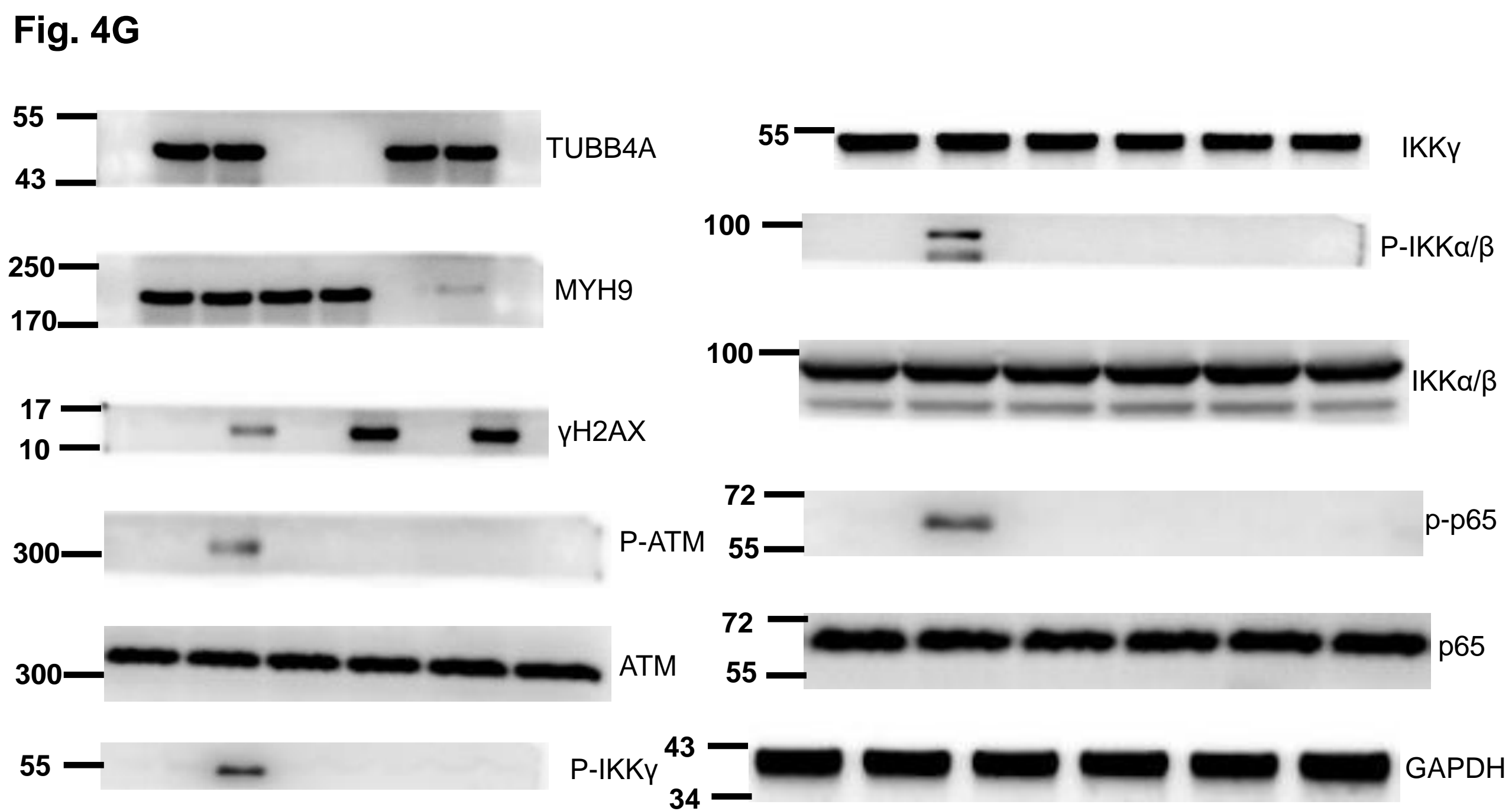

Fig. 4H

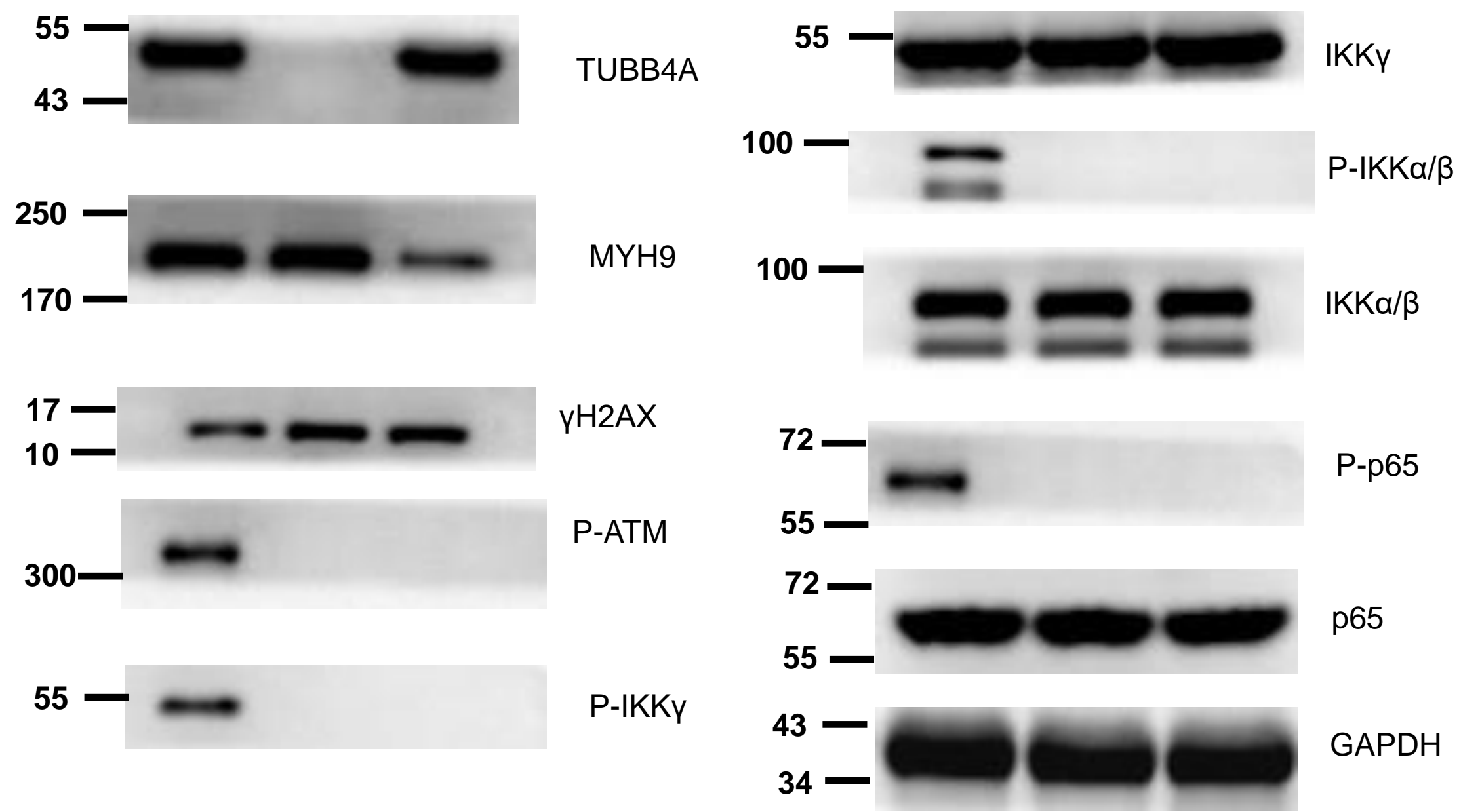

**Fig. 4I**

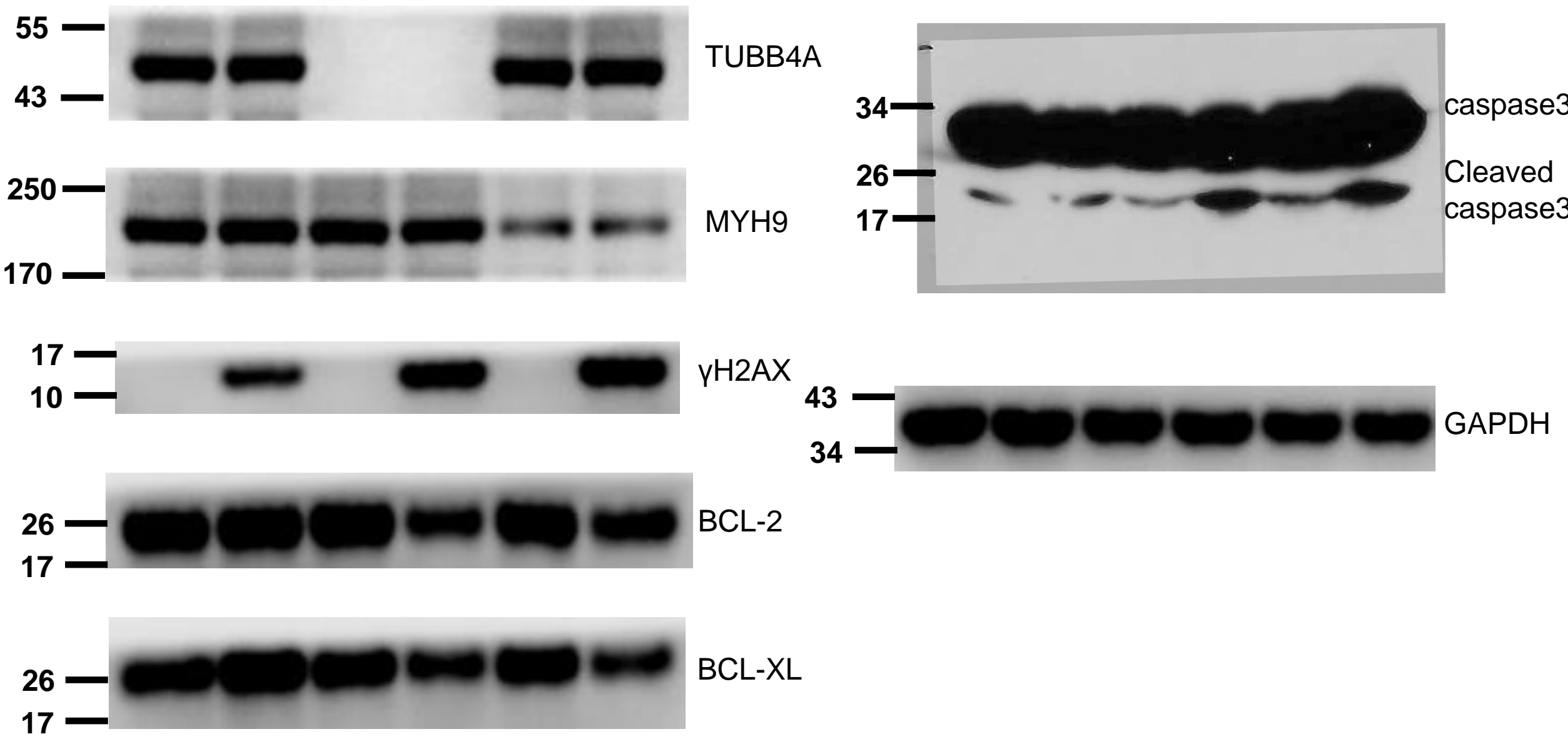

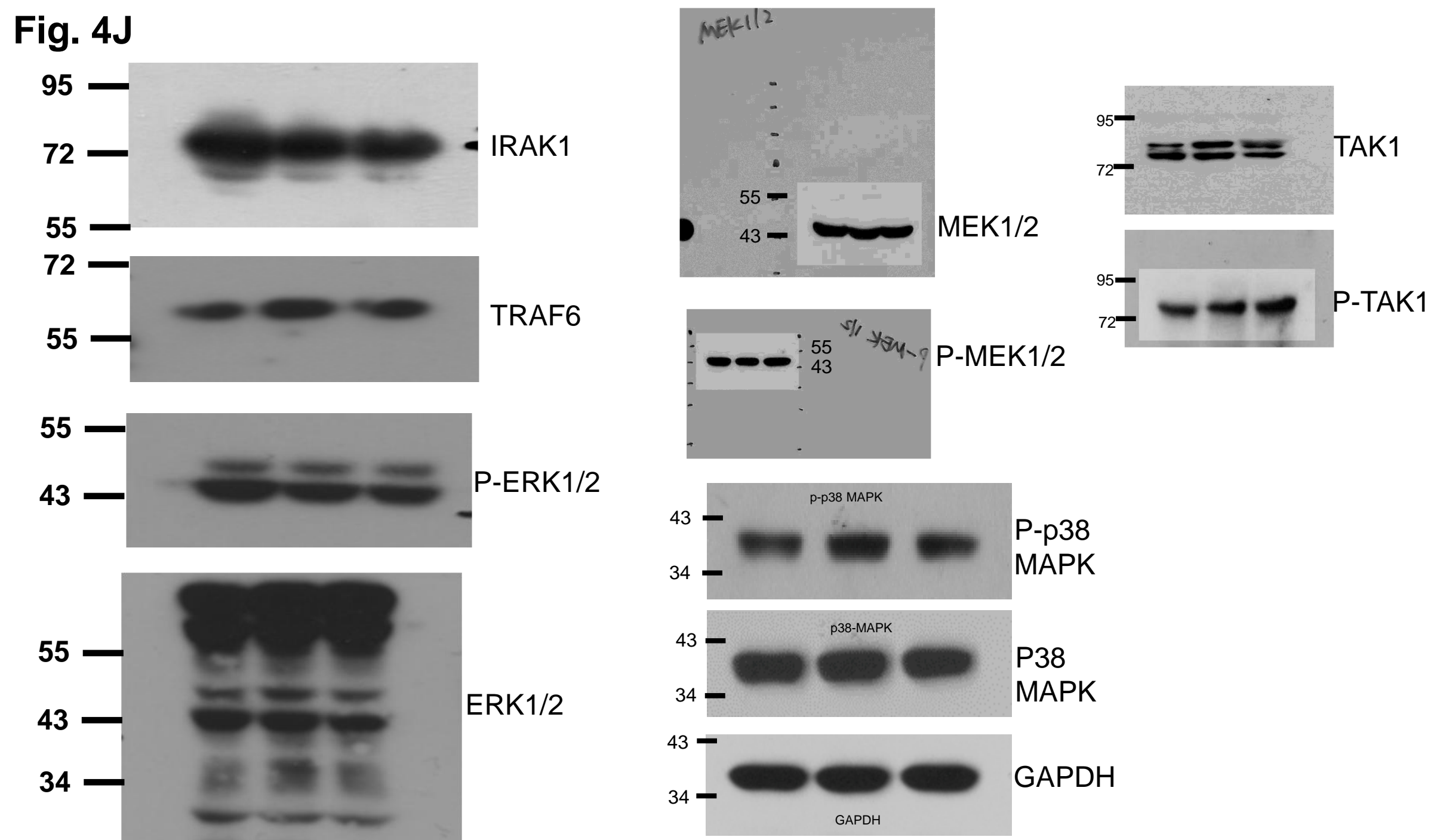

Supplement: Supplementary file 11 — Source Data [file 41467_2022_30409_MOESM11_ESM.zip › source-data/Figure 4/Fig. 4C, 4D, 4G, 4H, 4I, 4J.pdf]

**Fig. 5A**

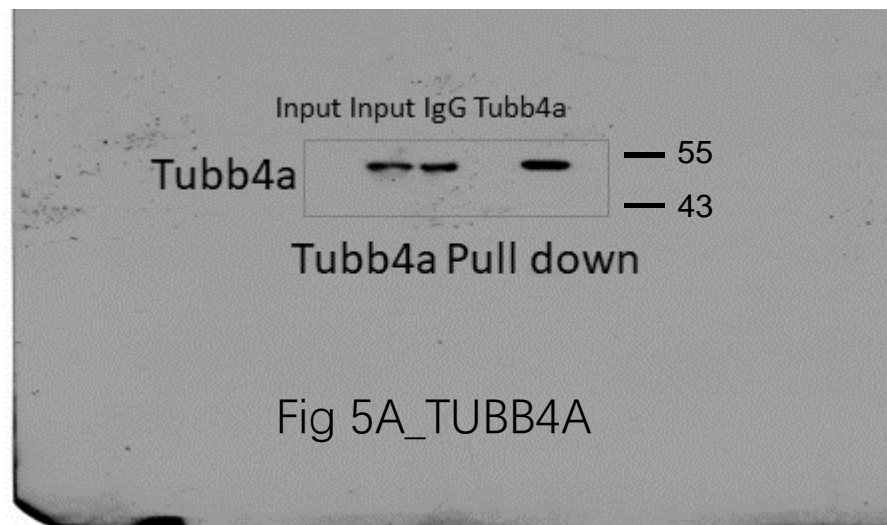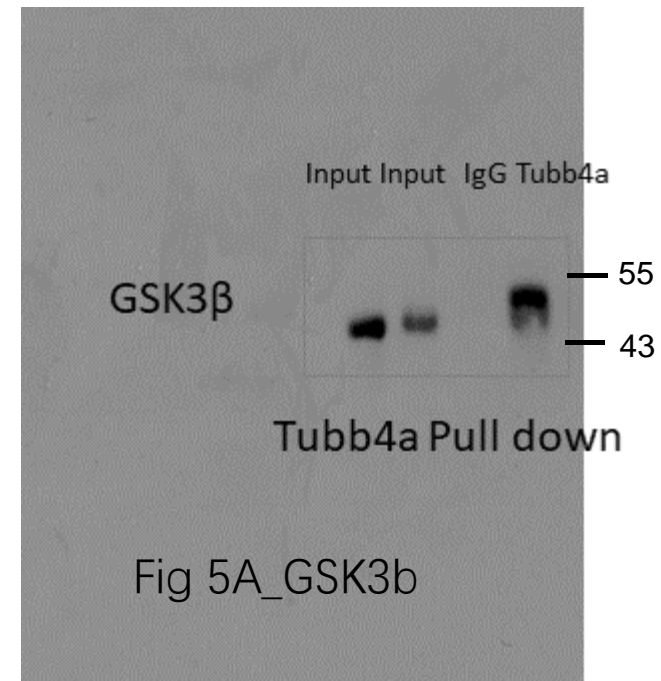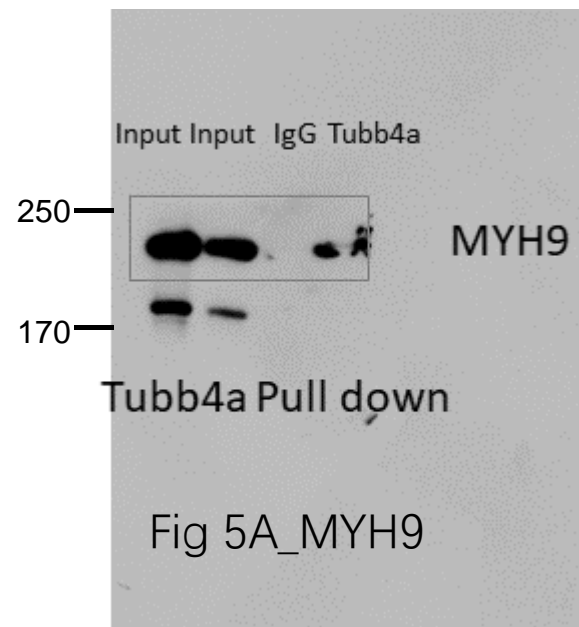

**Fig. 5B**

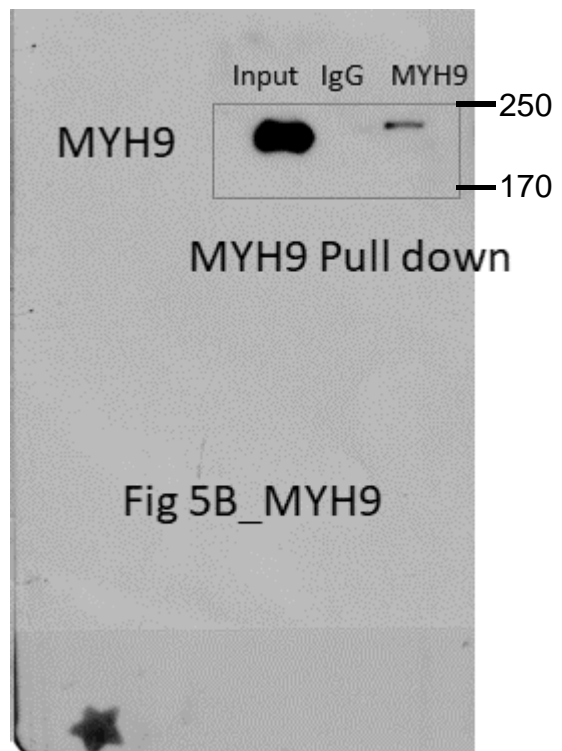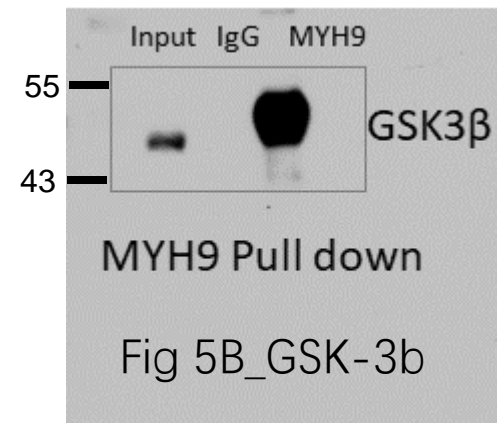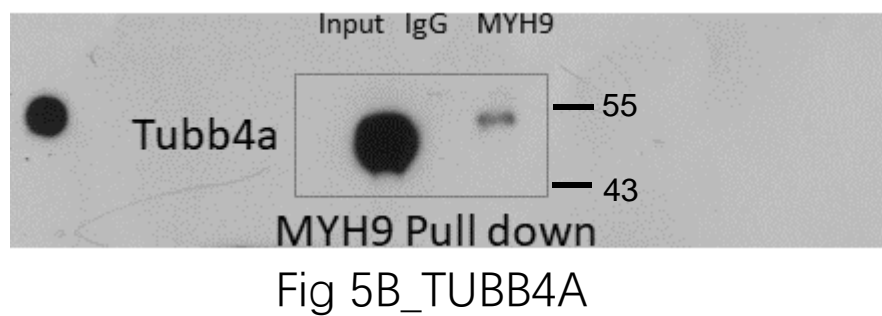

Fig. 5C-D

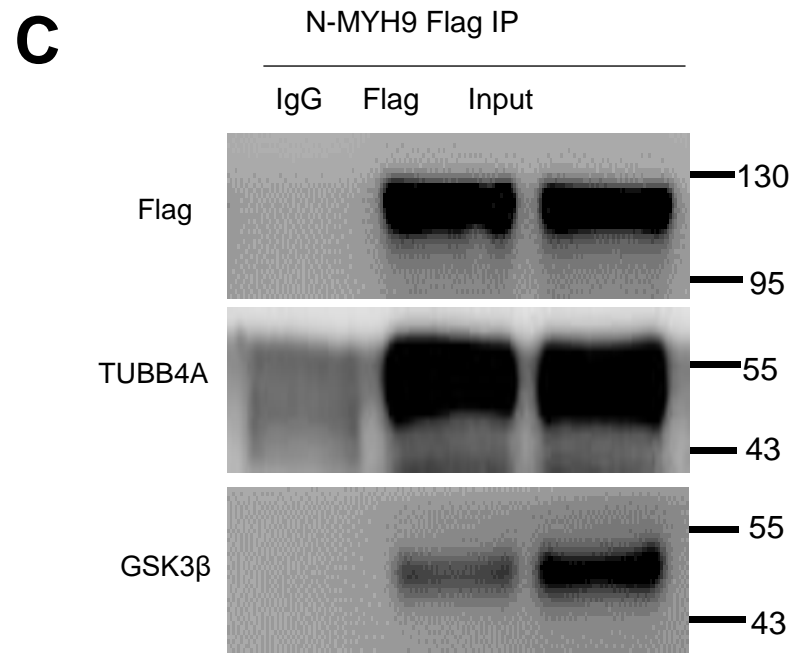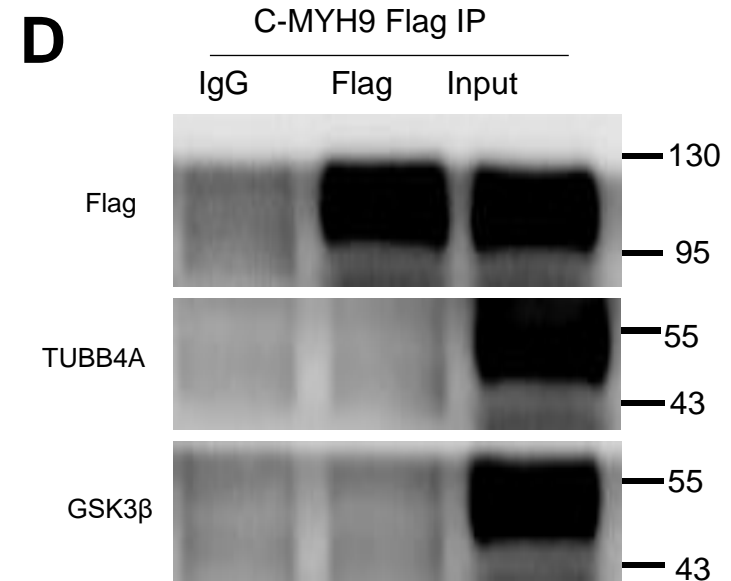

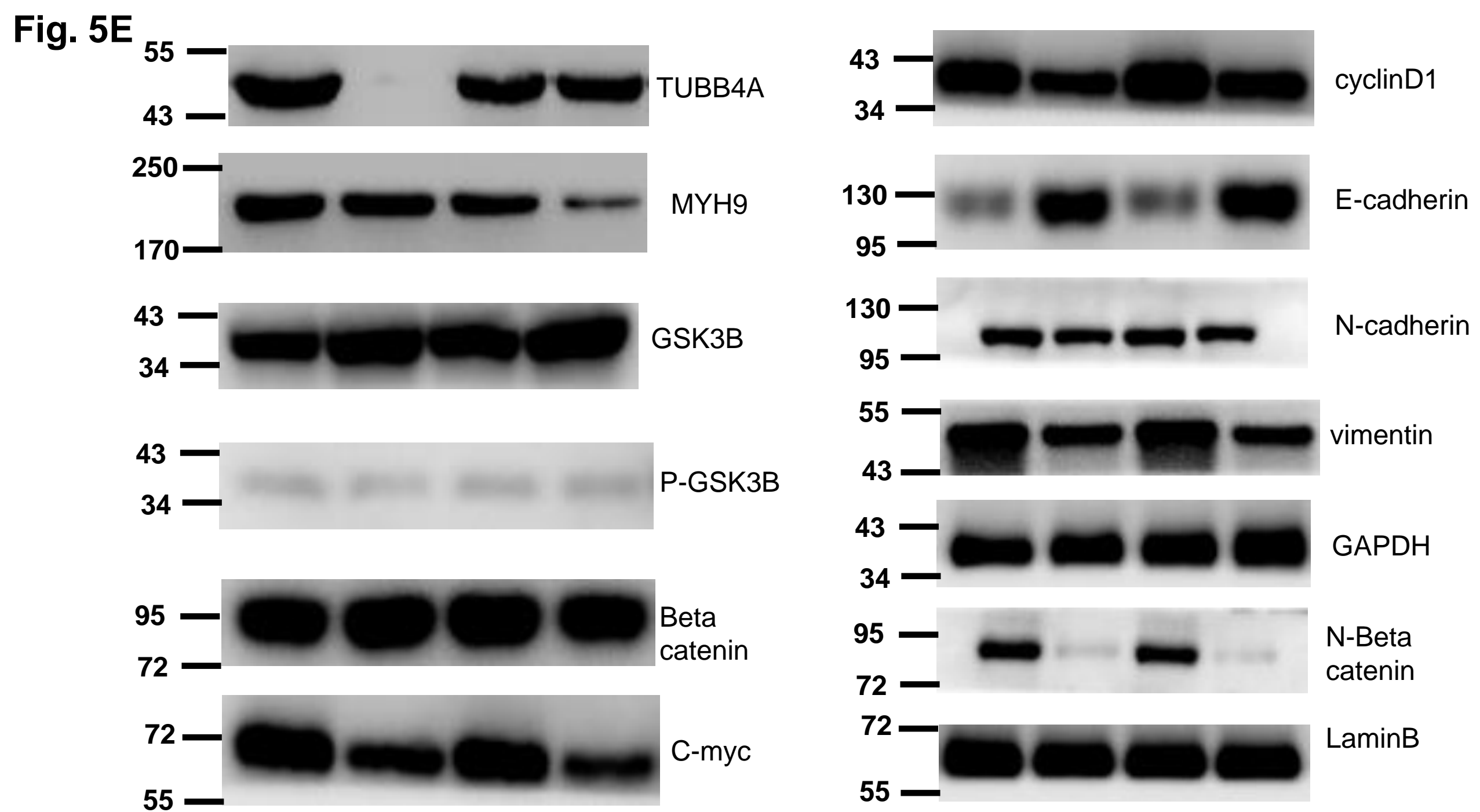

### Fig. 5F

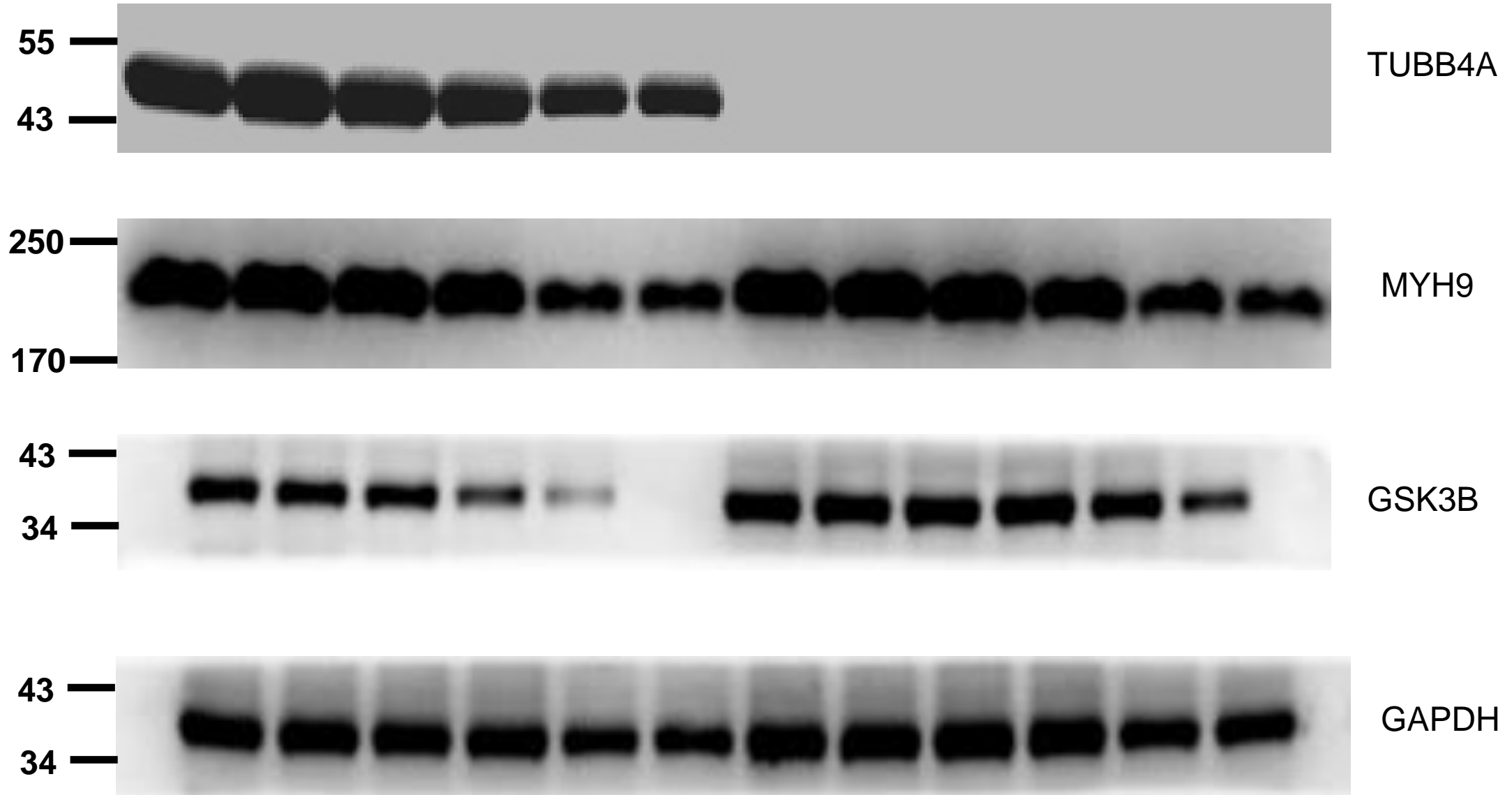

Fig. 5G

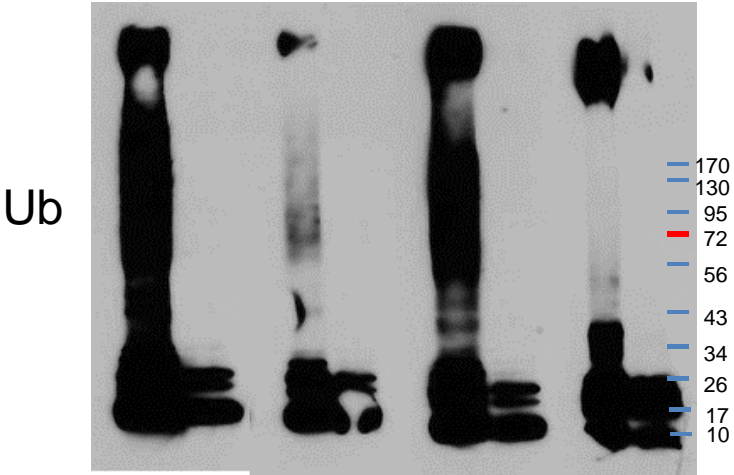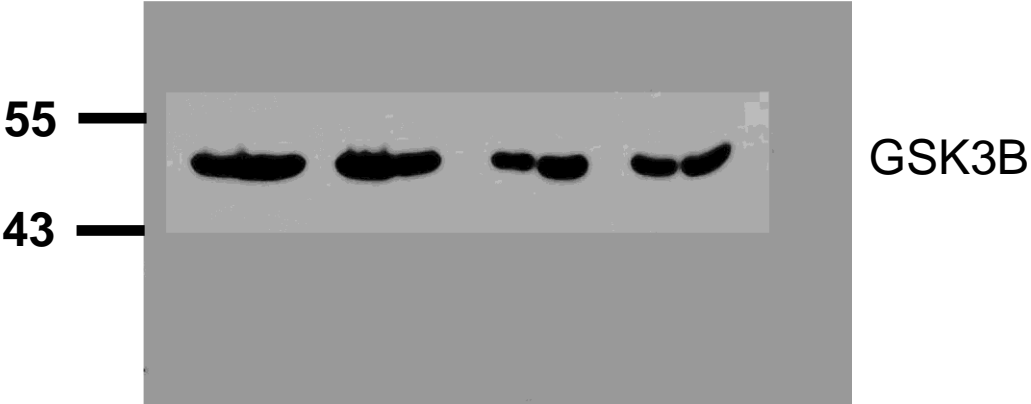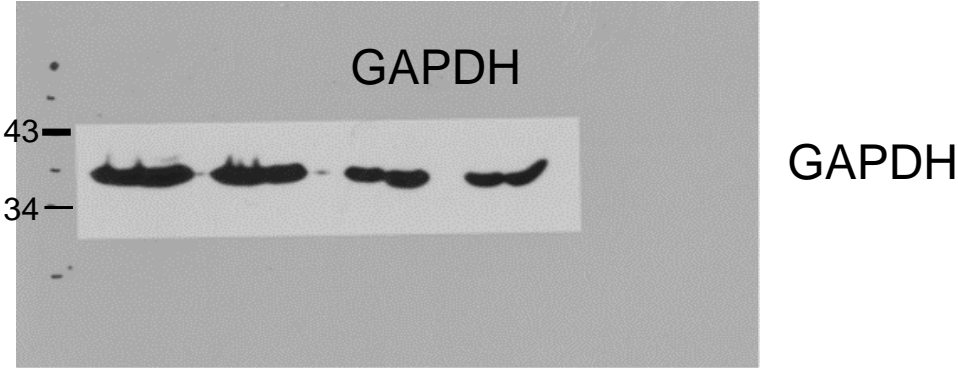

Supplement: Supplementary file 11 — Source Data [file 41467_2022_30409_MOESM11_ESM.zip › source-data/Figure 5/Fig. 5A-G.pdf]

**Fig. S2A, 2E**

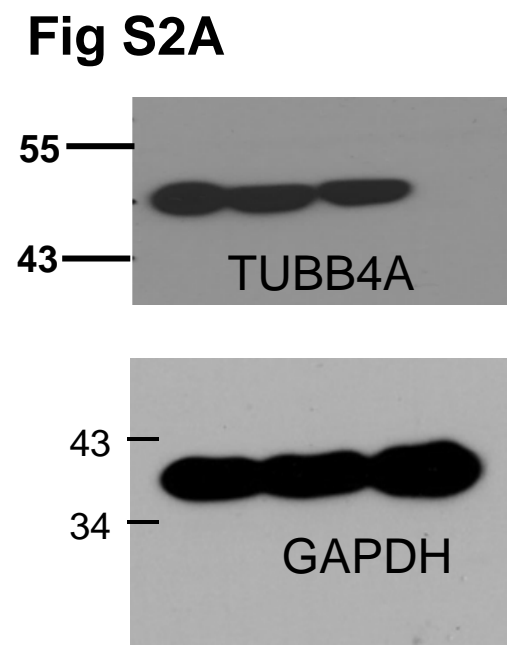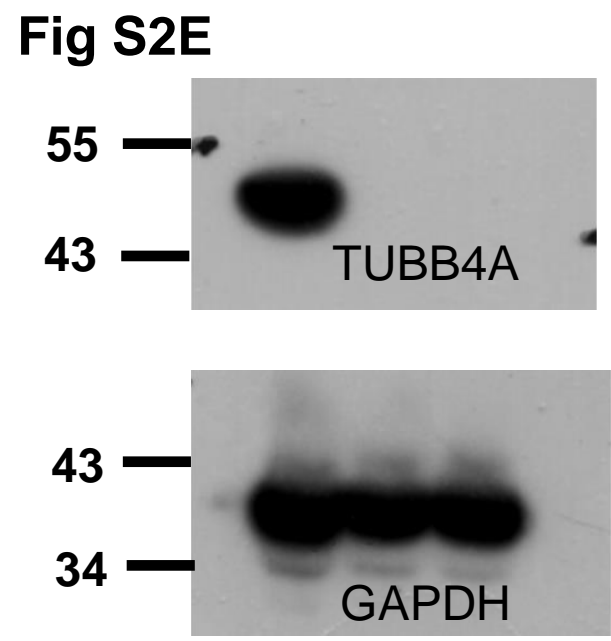

Supplement: Supplementary file 11 — Source Data [file 41467_2022_30409_MOESM11_ESM.zip › source-data/Supplementary figure 2/Fig. S2A, 2E.pdf]

**Fig. S2G**

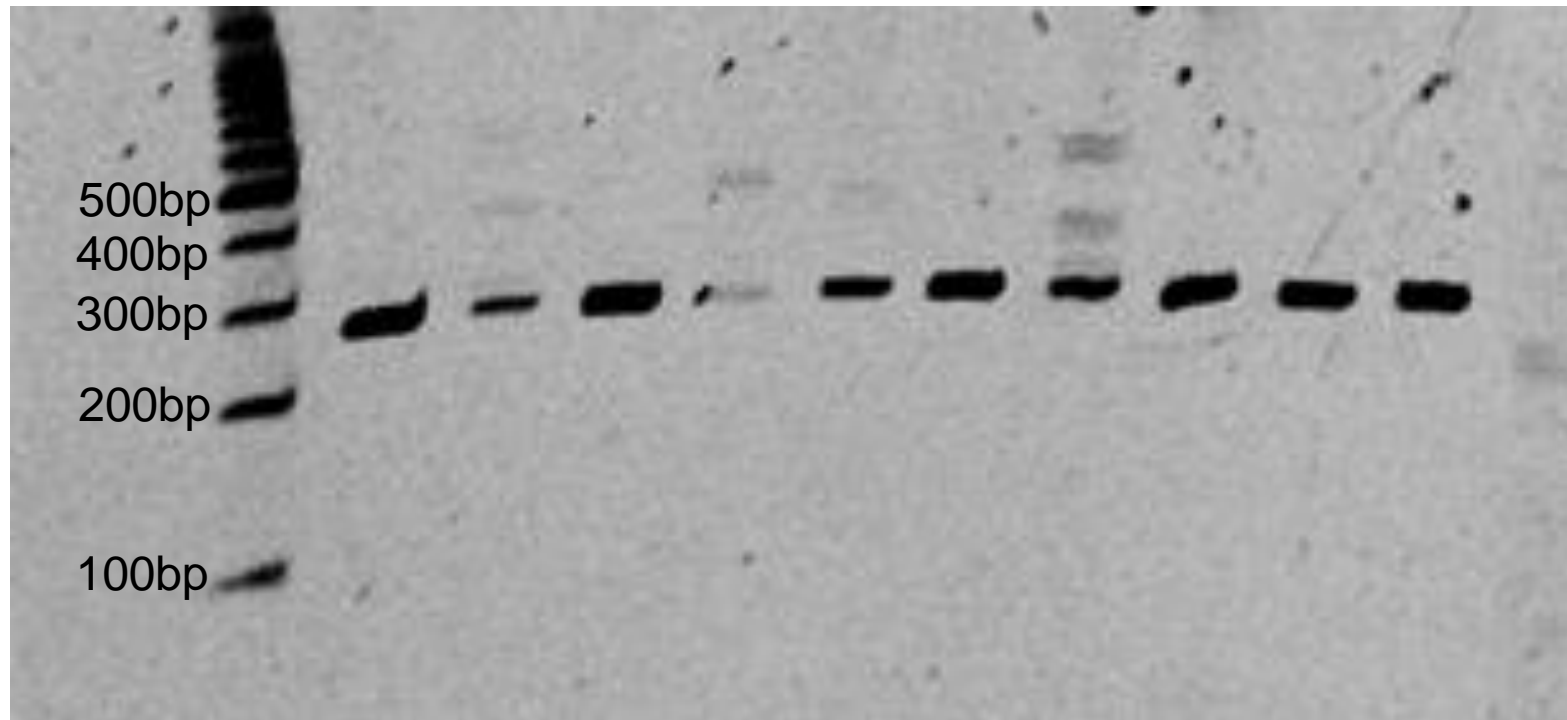

Supplement: Supplementary file 11 — Source Data [file 41467_2022_30409_MOESM11_ESM.zip › source-data/Supplementary figure 2/Fig. S2G.pdf]

**Fig. S4A**

**Fig S4A**

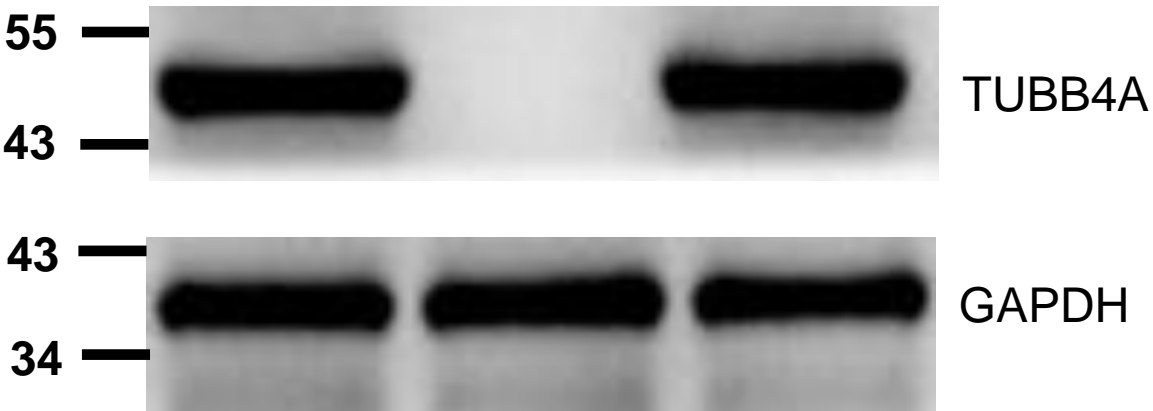

Supplement: Supplementary file 11 — Source Data [file 41467_2022_30409_MOESM11_ESM.zip › source-data/Supplementary figure 4/Fig. S4A.pdf]

**Fig. S8G, 8I**

**Fig S8G**

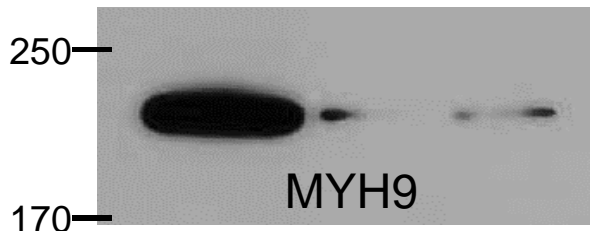

**Fig S8I**

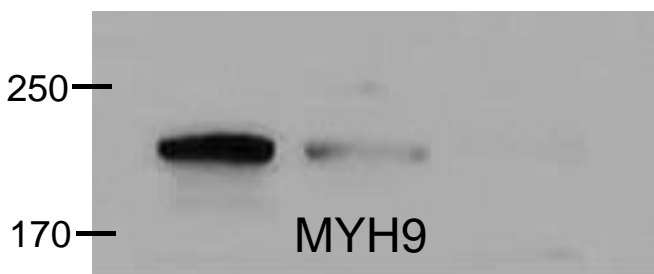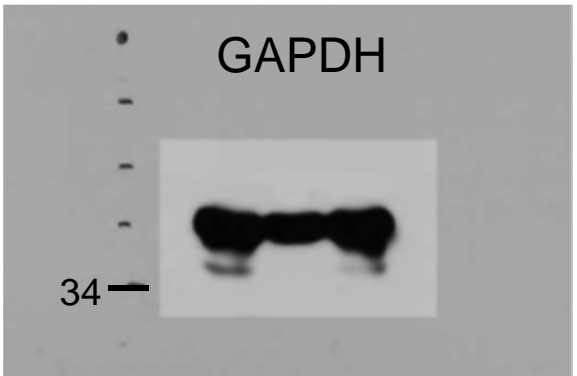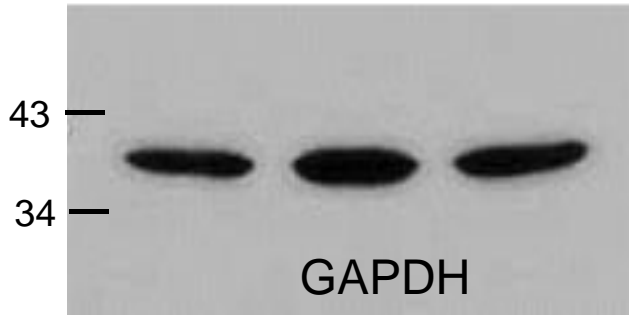

Supplement: Supplementary file 11 — Source Data [file 41467_2022_30409_MOESM11_ESM.zip › source-data/Supplementary figure 8/Fig. S8G, 8I.pdf]
